# Synthesis, fluorescence properties and the promising cytotoxicity of pyrene–derived aminophosphonates

**DOI:** 10.3762/bjoc.12.117

**Published:** 2016-06-16

**Authors:** Jarosław Lewkowski, Maria Rodriguez Moya, Anna Wrona-Piotrowicz, Janusz Zakrzewski, Renata Kontek, Gabriela Gajek

**Affiliations:** 1 Department of Organic Chemistry, Faculty of Chemistry, University of Łódź, Tamka 12, 91-403 Łódź, Poland; 2 Laboratory of Cytogenetics, Faculty of Biology and Environmental Protection, University of Łódź, Banacha 12/16, 90-237 Łódź, Poland

**Keywords:** aminophosphonic derivatives, cytotoxicity, fluorescence properties, MTT test, pyrene-1-carboxaldehyde

## Abstract

A large series of variously substituted amino(pyren-1-yl)methylphosphonic acid derivatives was synthesized using a modified aza-Pudovik reaction in 20–97% yields. The fluorescence properties of the obtained compounds were investigated revealing that *N*-alkylamino(pyren-1-yl)methylphosphonic derivatives are stronger emissive compounds than the corresponding *N*-aryl derivatives. *N*-Benzylamino(pyren-1-yl)methylphosphonic acid displayed strong fluorescence (Φ_F_ = 0.68) in phosphate-buffered saline (PBS). The influence of a series of derivatives on two colon cancer cell lines HT29 and HCT116 was also investigated. The most promising results were obtained for *N*-(4-methoxyphenyl)amino(pyren-1-yl)methylphosphonate, which was found to be cytotoxic for the HCT116 cancer cell line (IC_50_ = 20.8 μM), simultaneously showing weak toxicity towards normal lymphocytes (IC_50_ = 230.8 µM).

## Introduction

The biological activity of aminophosphonic systems is very well known and described, in aspect of their crop protective [1], antibacterial [2] or anticancer properties [3]. Various *C*-aryl substituted derivatives of phosphonoglycine have been synthesized including those of benzene and several polycyclic aromatic hydrocarbons [4], five- [5] and six-membered [6] heteroaromatic compounds and even ferrocene [7]. In contrast, pyrene-based derivatives, e.g., amino(pyrene-1-yl)methyl phosphonic acid derivatives have been described only twice in the chemical literature. Firstly, Harry Hudson’s team [8] published the synthesis of diethyl *N*-benzhydrylamino(pyrene-1-yl)methylphosphonate in a series of *N*-benzhydryl substituted aminomethyl phosphonates. Recently, an Indian group [9] reported the synthesis of a number of *N*-aryl substituted, diethyl amino(pyrene-1-yl)methyl phosphonates and some preliminary studies on their fluorescence properties.

Such a poor knowledge about these compounds is astonishing in the light of the fact that the phosphonic analogues of phenylglycine, exhibited their herbicidal activity [10], and are used as plant growth regulators [10], agrochemical fungicides [11–12] and glutamate receptor modulators [13]. It is therefore expectable that *C*-pyrene derivatives may have similar or even more promising biological properties.

This expectation is supported by the biological activity of azomethine derivatives of pyrene-1-carboxaldehyde. They show antimicrobial action, e.g., 1-phenytoinylacetic acid hydrazone of pyrene-1-carboxaldehyde demonstrated a moderate antimicrobial activity towards *Escherichia coli* and *Staphylococcus aureus* [14]. Azomethine derivatives of pyrene-1-carboxaldehyde have shown enzyme inhibitory activity, as, e.g., its thiosemicarbazone, which is able to inhibit the action of urease [15].

Although fluorescence properties of pyrene-based aminophosphonic derivatives have been mentioned only once in a preliminary form [9], fluorescence emission of their azomethine precursors was reported for pyrene-1-carboxaldehyde thiosemicarbazone and Schiff bases as well as their metal complexes [16–21]. Such properties were described for, e.g., ruthenium(II) complexes of (5-chloropyridin-2-yl)-(pyren-1-yl)methyleneamine [17], a *N*-(1-pyrene)methylideneglucosamine mercury complex [18], a *N*-(pyren-1-ylidene)-2-hydroxyaniline-copper(II) and -zinc(II) complexes [19] or *N*-(pyren-1-ylidene)-4-carboxyaniline-Fe(II) and -Cr(III) complexes [20]. Several phosphorus-supported ligands containing a pyrene-1-carbimino moiety [21] were found to be fluorescence-based sensors of Cu(II) and Mg(II) cations.

Considering all above, we decided to synthesize a series of *N*-substituted derivatives of *C*-(pyren-1-yl)phosphonoglycine and to investigate their cytotoxic and fluorescence properties.

## Results and Discussion

### Synthesis of aminophosphonic acid derivatives **3**, **4** and **5**

Schiff bases **1a**–**h** were prepared by refluxing pyrene-1-carboxaldehyde with an amine in methanol, hexane or dichloromethane for 24 hours and were used for further conversions as obtained. This harsh method to prepare imines had to be used, because the simple mixing of reagents in methanol at room temperature, which is the common *mode d’emploi* in such cases [5,7], did not provide satisfactory results. The reaction completion was monitored by ^1^H NMR and obtained imines **1a**–**h** were isolated by simple evaporation of the solvent and were used for the further reaction without purification.

**Scheme 1:**
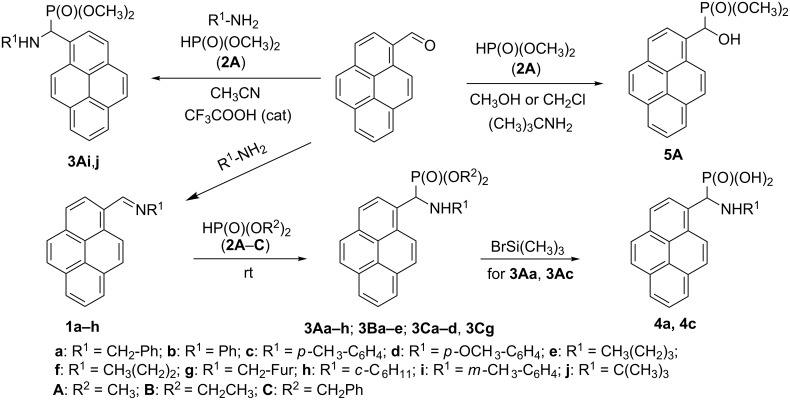
Synthesis of aminophosphonates **3Aa**–**j**, **3Ba**–**e**, **3Ca**–**d**, **3Cg**, aminophosphonic acids **4a**, **4c** and hydroxyphosphonate **5A**.

Aminophosphonates **3Aa**–**h**, **3Ba**–**e** and **3Ca**–**d**, **3Cg** have been synthesized via an aza-Pudovik reaction [22], i.e., the addition of the appropriate phosphite **2A**–**C** to the azomethine bond of the Schiff bases **1a**–**h**. Nevertheless, the important modifications had to be introduced to the described procedures [22–23]. The 1-pyrene moiety is a troublesome substituent due to its spatial volume, which tends to disturb in the course of the reactions. Attempts to use any of common solvents failed as no or only a weak progress of the reaction was noticed (Scheme 1). The best solution was to apply phosphites simultaneously as solvents and reactants, which however forced to use them in a high excess. This necessity resulted in troubles with purification, implicating a particular approach to each case.

Dimethyl aminophosphonates **3Aa**–**Ah** were purified by washing their dichloromethane solutions with a saturated aqueous sodium bicarbonate solution, which allowed to remove dimethyl phosphite. This procedure was followed by column chromatography, and, in a case of *N*-benzyl derivative **3Aa**, triturating the product with diethyl ether until the precipitation of a solid was additionally carried out.

The isolation and purification of diethyl aminophosphonates **3Ba**–**e** required a different method. To isolate them, the crude reaction mixtures were dissolved in a minimum amount of diethyl ether and triturated until a yellow precipitate formed. This operation was repeated twice. Aminophosphonates **3Ba**–**e** were purified by column chromatography.

The most complex procedure was used to isolate and to purify the dibenzyl aminophosphonates **3Ca**–**d** and **3Cg**. The crude reaction mixtures were dissolved in small amounts of pyridine and then, treated with elemental iodine to decompose dibenzyl phosphite. This was followed by washing the mixture dissolved in dichloromethane with saturated aqueous solutions of sodium thiosulfate and then sodium bicarbonate. The purification with column chromatography allowed to afford pure products in satisfactory yields.

Due to the fact that we were not able find proper conditions to prepare *N*-(pyren-1-ylidene)-*tert*-butylamine (**1j**) and dimethyl *N*-(*tert*-butyl)amino(pyren-1-yl)methylphosphonate (**3Aj**) via the aza-Pudovik reaction, the Kabachnik–Fields reaction was used as an alternative method [23]. The three-component reaction without any catalyst was carried out first in dichloromethane then, in methanol. In both cases, to our surprise, no traces of the desired aminophosphonate **3Aj** have been found in the reaction mixture. Instead, dimethyl hydroxy(pyren-1-yl)methylphosphonate (**5A**) was obtained in quite satisfactory yield. Dimethyl *N*-(*tert*-butyl)amino(pyren-1-yl)methylphosphonate (**3Ai**) was obtained, when the Kabachnik–Fields reaction was carried out in the presence of a catalytic amount of trifluoroacetic acid in refluxing acetonitrile. The same procedure, i.e., the Kabachnik–Fields reaction in the presence of trifluoroacetic acid, but in refluxing methanol has been successfully used for the preparation of dimethyl *N*-(*m*-methylphenyl)amino(pyren-1-yl)methylphosphonate (**3Ai**) (Scheme 1).

Interestingly, attempts to obtain the hydroxyphosphonate **5A** in conditions typical for the Pudovik (not aza) reaction failed – the product could be obtained only when *tert*-butylamine (or isopropylamine) was present in the reaction mixture (Scheme 1).

All obtained aminophosphonates **3Aa**–**j**, **3Ba**–**e**, **3Ca**–**d** and **3Cg** as well as the hydroxyphosphonate **5A** gave satisfactory results of in the elemental analysis and were characterized by means of ^1^H, ^13^C and ^31^P NMR and IR spectroscopy with all signals attributed to each nucleus. Compounds were also characterized by melting point measurements.

Jayaprakash et al. [9] reported the synthesis of several pyrene-derived aminophosphonates via a silica-catalyzed Kabachnik–Fields reaction. Among others, they synthesized three amino-phosphonates **3Bb**–**d**, reporting their melting point values and their spectral characterizations. Melting point values quoted by the authors differed from values measured by us. The reason of such a discrepancy remains unclear, it might be caused by the different eluents used – we eluted products using chloroform, while Jayaprakash et al*.* [9] used hexane and ethyl acetate (2:3).

The other discrepancy concerns the ^1^H NMR spectra, and particularly their aromatic regions. In all three cases, Jayaprakash et al*.* [9] quoted all aromatic signals as broad multiplets, whereas we were capable to distinguish clearly signals of pyrene protons as a series of distinct doublets and approximate triplets. Phenyl ring signals were also visible, e.g., the non-substituted phenyl ring gave a triplet, doublet of doublets and a doublet, the 4-methyl substituted phenyl moiety demonstrated a distinct AA’XX’ system, whereas the 4-methoxy derivative shows an AA’BB’ system. All these phenomena are clearly visible in the ^1^H NMR spectra and scans of them are collected in Supporting Information File 1.

Aminophosphonic acids **4a** and **4c** were prepared using the classical cleavage of dimethyl aminophosphonates **2Aa** and **2Ac** with trimethylsilyl bromide in dry dichloromethane. After 24-hours reaction, the reaction was quenched by methanol to give, after routine work-up the desired acids **4a** and **4c** (Scheme 1). They were characterized by NMR spectroscopy (^1^H, ^13^C and ^31^P), IR spectroscopy, melting point measurements and elemental analysis.

The obtained phosphonic derivatives were then selected and their biological and photophysical properties were studied.

### Luminescent properties of pyrenyl aminophosphonates

**Figure 1:**
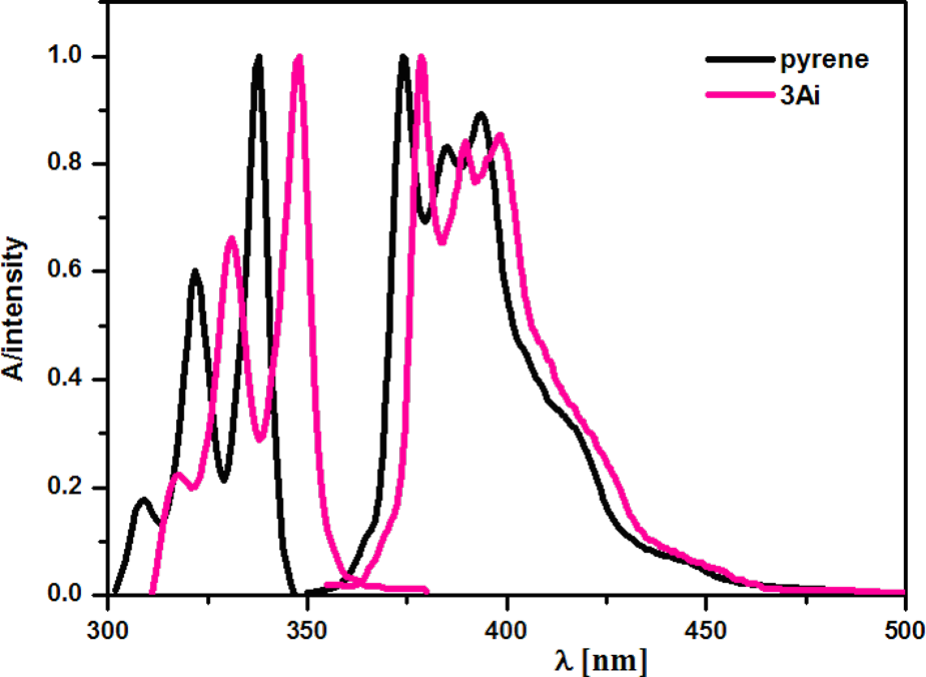
Normalized electronic absorption and emission spectra of **3Aj** and pyrene in chloroform (*c* = 1 μM).

The widespread use of pyrene derivatives as luminescent probes in biological research [24–27] prompted us to study emissive properties of the synthesized aminophosphonates. We have measured their electronic absorption and emission spectra in chloroform solution. Although photodecomposition of pyrene in this solvent has been reported [28], we found that this is not the case of the compounds under study. To avoid excimer formation, spectra were recorded at low sample concentrations (1 μM). We have found that all compounds under study were emissive albeit emission quantum yields varied from ≈0.01 to ≈0.7 (Table 1). The typical absorption and emission spectrum (those of compound **3Aj**), compared with the spectra of pyrene are shown in Figure 1. The spectra are closely similar and show vibronic structure characteristic for monomeric fluorophores. The spectra of **5** are slightly shifted bathochromically (9 nm and 4 nm for the absorption and emission spectrum, respectively) in comparison to the spectra of pyrene. In this context, it should be noticed that spectra reported in [9] were measured at significantly higher concentrations (0.5 mM) and are broad and structureless. Therefore, they should rather be assigned to fluorophore aggregates (excimers).

**Table 1:** Absorption and emission maxima and fluorescence quantum yields (Φ_F_) for the synthesized compounds in chloroform solution (*c* = 1 μM).

Compd.	Absorption λ_max_ (nm)	Emission^a^ λ_max_ (nm)	Φ_F_^b^

**3Aa**	304, 317, 330, 347	378, 388, 399	0.06
**3Ba**	304, 316, 331, 347	378, 388, 398	0.06
**3Ca**	304, 316, 331, 348	378, 389, 399	0.06
**3Ab**	304, 317, 331, 348	382, 397, 424, 444	<0.01
**3Bb**	304, 318, 330, 348	382, 397, 443	<0.01
**3Cb**	305, 318, 332, 349	382, 397, 455	<0.01
**3Ac**	304, 317, 331, 348	380, 395, 485	<0.01
**3Bc**	304, 316, 330, 448	380, 395, 484	<0.01
**3Cc**	304, 317, 330, 448	380, 396, 485	<0.01
**3Ad**	304, 317, 331, 348	380, 398,	<0.01
**3Bd**	304, 317, 330, 348	380, 398	<0.01
**3Cd**	304, 317, 331, 348	381, 398	<0.01
**3Ae**	303, 316, 330, 347	378, 390, 398	0.10
**3Be**	303, 316, 330, 346	377, 389, 397	0.10
**3Af**	303, 317, 330, 347	378, 390, 398	0.09
**3Ag**	316, 330, 347	378, 390, 398	0.05
**3Ai**	303, 316, 330, 347	380, 395	<0.01
**3Aj**	318, 331, 347	378, 389, 397	0.11
**4a^c^**	303, 316, 329, 345	378, 397, 419	0.68
**5A**	317, 330, 347	377, 388, 396	0.13

^a^Excitation was set at the maximum of the lowest energy absorption band. ^b^Measured for aerated solutions. Quenching by dissolved dioxygen was not observed. ^c^Measured for an aqueous 0.01 PBS (phosphate-buffered saline) solution (pH 7.4)

**Figure 2:**
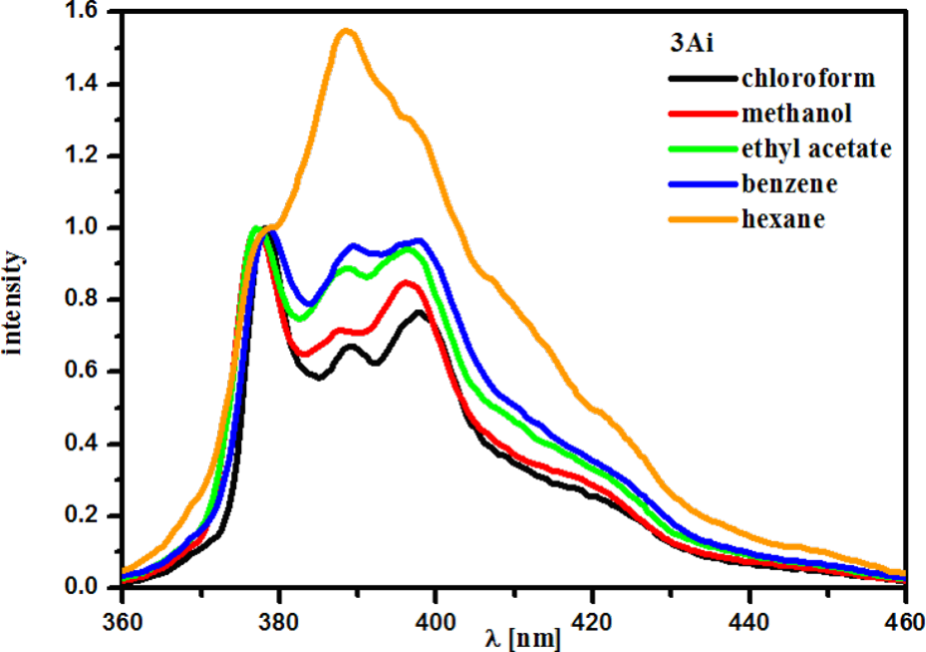
Emission spectra of compound **3Aj** in various solvents. The spectra are normalized at ≈378 nm (pyrene band I).

The spectra of **3Aj** were run in solvents of varied polarity (Figure 2) and display the well-known Ham effect (dependence of the ratio of intensities of III/I vibronic bands on the medium polarity) [26].

The highest emission quantum yield (0.68) was found for aminophosphonic acid **4a** in aqueous buffer (pH 7.4). A similar phenomenon of higher emission efficiency of 1-(pyrene-1-carboxamido)methylphosphonic acid in comparison to a corresponding aminophosphonate was earlier reported [29].

The aforementioned data suggest possible application of compound **3Aj** (and other exhibiting similar emission quantum yield) as molecular probes monitoring micropolarity of the fluorophore environment. On the other hand, water-soluble **4a** might be used for biological imaging.

### Investigation of cytotoxic effects of studied compounds

The cytotoxic effects of dimethyl aminophosphonates **3Aa**, **3Ab**, **3Ac**, **3Ad**, **3Ai** and dimethyl [hydroxy(pyren-1-yl)methyl]phosphonate (**5A**) were investigated with two human colorectal carcinoma cell lines: HT29 and HCT116 and also on the normal human lymphocytes. For this a standard MTT (3-(4,5-dimethylthiazol-2-yl)-2,5-diphenyltetrazolium bromide) test was used and results presented as IC_50_ values (i.e., the concentration of the compound that inhibits 50% growth). The activity of the tested compounds was studied in the concentration range from 5 to 600 µM. The obtained IC_50_ values are summarized in Table 2.

**Table 2:** IC_50_ values on studied compounds.

Compound	R^1^	lymphocytes	HT29	HCT116

**3Aa**	CH_2_Ph	IC_50_ = 89.2 ± 2.9 µM	IC_50_ = 29.2 ± 3.84 µM	IC_50_ = 29 ± 2.37 µM
**3Ab**	Ph	IC_50_ = 334.2 ± 2.59 µM	IC_50_ = 105.8 ± 3.37 µM	IC_50_ = 70.8 ± 2.99 µM
**3Ac**	4-C_6_H_4_-CH_3_	IC_50_ = 17.5 ± 4.15 µM	IC_50_ = 15.8 ± 3.44 µM	IC_50_ = 15.9 ± 4.85 µM
**3Ad**	4-C_6_H_4_-OCH_3_	IC_50_ = 230.8 ± 3.43 µM	IC_50_ = 24.2 ± 5.12 µM	IC_50_ = 20.8 ± 3.48 µM
**3Ai**	3-C_6_H_4_-CH_3_	IC_50_ = 75.8 ± 4.02 µM	IC_50_ = 57.5 ± 5.19 µM	IC_50_ = 20.21 ± 5.63 µM
**5A**	–	IC_50_ = 135.8 ± 1.73 µM	IC_50_ = 25.8 ± 4.99 µM	IC_50_ = 40.4 ± 4.53 µM

**Figure 3:**
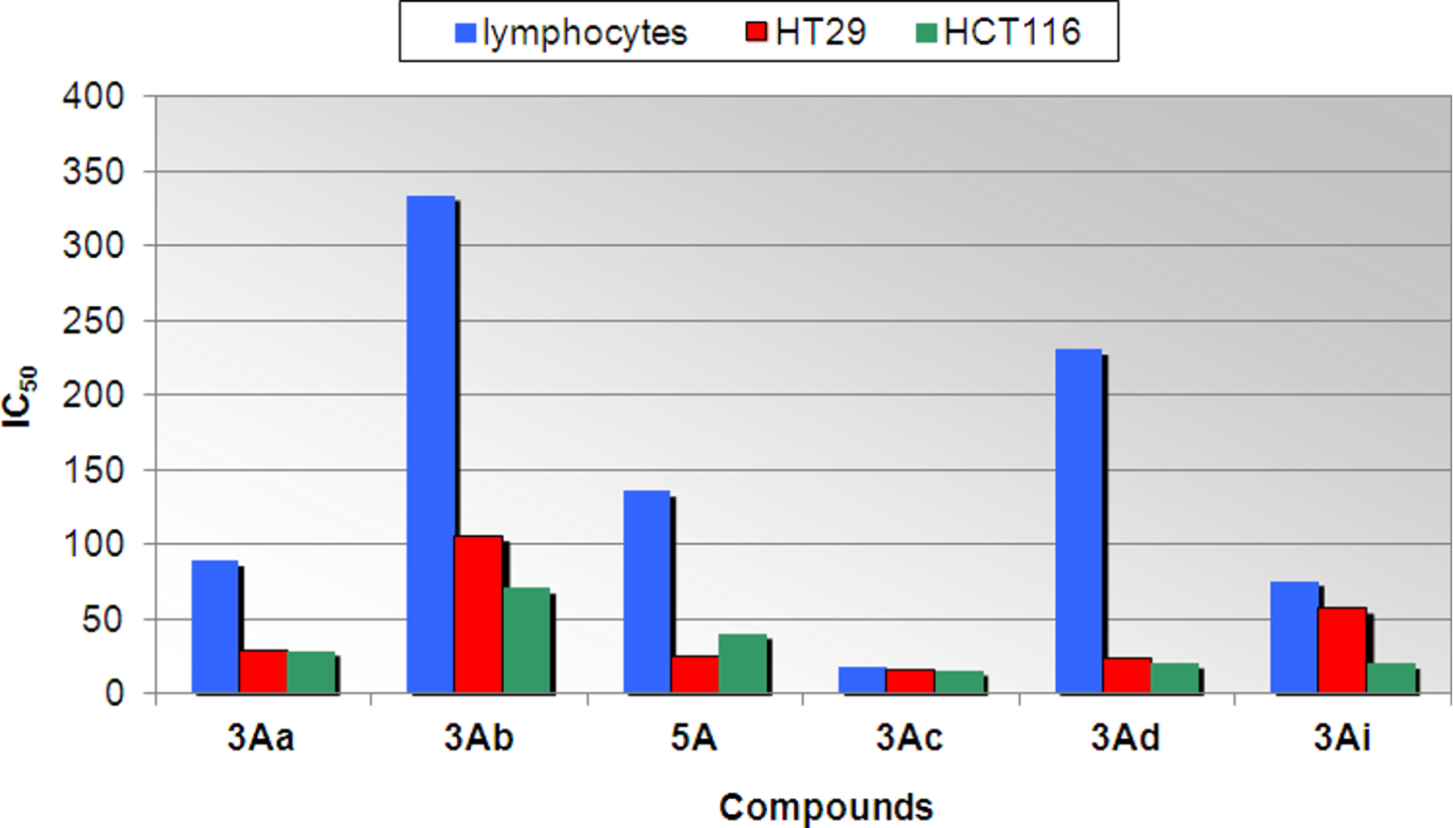
IC_50_ values of studied compounds.

The activity of tested compounds varied from IC_50_ = 15.8 µM to 105.8 µM. As it can be seen, the most interesting results were obtained for compound **3Ad**, bearing the *p*-methoxyphenyl moiety (Figure 3). It has exhibited significant cytotoxicity against both tested cancer cell lines HT29 (IC_50_ = 24.2 µM) as well as HCT116 (IC_50_ = 20.8 µM) cells, and it simultaneously was not strongly toxic for normal human lymphocytes (IC_50_ = 230.8 µM). However, HT29 cells were more resistant to **3Ad** than HCT116 cells. Therefore, the aminophosphonate **3Ad** showed a carcinoma-specific cytotoxicity against human colon cancer cells. A similar correlation was observed for the aminophosphonic derivative **3Ac**, which was strongly cytotoxic against both colon cancer cell lines, but in contrast to **3Ad**, was also toxic for lymphocytes. Paradoxally, compound **3Ac**, bearing a 4-C_6_H_4_-CH_3_ moiety, exhibited the most potent cytotoxicity from all tested compounds: IC_50_ (HT29) = 15.8 µM and IC_50_ (HCT116) = 15.9 µM. On the other hand, **3Ac** had the highest IC_50_ value against normal cells from all tested compounds. The compound **3Ab** was the less toxic for normal cells (IC_50_ = 334.2 µM) from among all tested compounds. However, **3Ab** was also the least toxic against both tested cancer cell lines HT29 (IC_50_ = 105.8 µM) and HCT116 (IC_50_ = 70.8 µM). Compound **5A** has exhibited a significant cytotoxicity against HT29 (IC_50_ = 25.8 µM). For compounds **3Aa** and **3Ai**, the received results were comparable, except for the results against HT29, the level of the cytotoxicity was slightly higher for **3Ai**. Simultaneously, cytotoxicities of both aminophosphonates (**3Aa** and **3Ai**) against normal lymphocytes were rather high (IC_50_ = 89.2 and 75.8 µM, respectively).

On the basis of the results presented in Table 1, it can be concluded that the type of functional group has a large influence on the biological properties of the tested compounds. These results indicate that the presence of a methylphenyl group causes a compound to be toxic for normal lymphocytes (**3Ac** and **3Ai**); a similar phenomenon was observed for *N*-benzyl substitution (**3Aa**). Results obtained for the α-hydroxyphosphonate **5A** (IC_50_ = 25.8 µM against HT29) give the impact to look for potential anticancer agents among the α-hydroxyphosphonic derivatives.

## Conclusion

A large series of various amino(pyren-1-yl)methylphosphonates was prepared using the aza-Pudovik reaction (**3Aa**–**h**, **3Ba**–**e**, **3Ca**–**d**, **3Cg**) or in the course of the Kabachnik-Fields reaction (**3Ai** and **j**). Amino(pyren-1-yl)methylphosphonic acids **4a** and **4c** were prepared using Boduszek’s methodology [6] and dimethyl hydroxy(pyren-1-yl)methylphosphonate (the Pudovik reaction). The fluorescence properties of the obtained compounds were investigated and *N*-benzylamino(pyren-1-yl)methylphosphonic acid showed a quantum yield of 68%, while dimethyl *tert*-butylamino(pyren-1-yl)methylphosphonate (**3Aj**) gave 11% quantum yield.

The influence of a series of derivatives on two colon cancer cell lines HT29 and HCT116 was also investigated. The most interesting results were obtained for dimethyl *N*-(4-methoxyphenyl)amino(pyren-1-yl)methylphosphonate **4d**, which was found to be cytotoxic for these two colon cancer cell lines (IC_50_ ≈ 20 μM), but showed nearly no toxicity towards lymphocytes (IC_50_ ≈ 230 μM). Although, dimethyl *N*-(4-methylphenyl)amino(pyren-1-yl)methylphosphonate **4c** demonstrated important cytotoxicity (IC_50_ ≈ 15 μM) for both cancer cell lines, it was unfortunately found to be toxic towards normal lymphocytes (IC_50_ ≈ 17 μM).

More profound studies on biological properties of selected compounds (necrosis vs apoptosis, mechanism of action) are being developed and will be published in the near future.

## Supporting Information

File 1 Experimental procedures, characterization of novel compounds, and details of the biological and photophysical study. Scans of ^1^H, ^13^C and ^31^P NMR spectra of all new synthesized compounds.
