# Supplementary material for: Synthesis, fluorescence properties and the promising cytotoxicity of pyrene–derived aminophosphonates
Source: Beilstein J Org Chem. 2016 Jun 16;12:1229–35. doi: 10.3762/bjoc.12.117 (PMC4979872; doi:10.3762/bjoc.12.117)

Supporting Information

for

Synthesis, fluorescence properties and the promising cytotoxicity of pyrene–derived aminophosphonates

Jarosław Lewkowski*[1], Maria Rodriguez Moya[1], Anna Wrona-Piotrowicz[1], Janusz Zakrzewski*[1], Renata Kontek*[2], Gabriela Gajek[2]

Address: [1]Department of Organic Chemistry, Faculty of Chemistry, University of Łódź, Tamka 12, 91-403 Łódź, Poland and [2]Laboratory of Cytogenetics, Faculty of Biology and Environmental Protection, University of Łódź, Banacha 12/16, 90-237 Łódź, Poland

Email: Jarosław Lewkowski - jlewkow@uni.lodz.pl; Renata Kontek - renkon@biol.uni.lodz.pl; Janusz Zakrzewski - janzak@uni.lodz.pl

*Corresponding author

Experimental procedures, characterization of novel compounds, and details of the biological and photophysical study. Scans of ^{1}H, ^{13}C and ^{31}P NMR spectra of all new synthesized compounds

Experimental section **Page s2**

1. Synthesis of studied compounds **Page s2**
2. Biological studies **Page s13**
3. Fluorescence studies **Page s14**
4. References to the experimental part **Page s14**

Scans of ^{1}H NMR spectra of studied compounds **3**, **4** and **5A** **Page s15**

Scans of ^{13}C NMR spectra of studied compounds **3**, **4** and **5A** **Page s27**

Scans of ^{31}P NMR spectra of studied compounds **3**, **4** and **5A** **Page s39**

Experimental section

1. Synthesis of studied compounds

1.1. General information

All solvents were applied routinely and dried prior to use. Commercial reagents were generally used as received. NMR spectra were recorded on a Bruker Avance III 600 MHz apparatus operating at 600 MHz (^{1}H NMR), 150 MHz (^{13}C NMR) and 243 MHz (^{31}P NMR). IR spectra were recorded on a Thermo Nicolet Nexus FT-IR spectrometer. Melting points were measured using a MelTemp II apparatus, in a capillary. Elemental analyses were performed in the Laboratory of Microanalysis, the Center of Molecular and Macromolecular Studies PAS in Łódź.

1.2. Preparation of phosphonates 3Aa–h, 3Ba–e and Ca–d via the modified aza-Pudovik reaction

Equimolar quantities (1–2 mmol) of pyrene-1-carboxaldehyde and amine were dissolved in methanol (**1a, 1e, 1f, 1h**) or hexane (**1b–d**) dichloromethane (**1g**) and stirred at reflux for 24 h (**1a–c, 1g, 1h**) or 48 h (**1d, 1e, 1f**). The reaction was monitored by ^{1}H NMR and after the completion, the solvent was removed under reduced pressure, and obtained products were used for the further conversion.

1 mmol of imine and 3 mmol (or 4–6 mmol in a case of dimethyl phosphite due to the low solubility of the imine in it) of the appropriate phosphite **2A–C** were placed into a 25 ml flask. It was heated with stirring on a water bath for 5 min until all of the imine was dissolved in phosphite. Then the mixture was stirred at room temperature for 24 h. Purification, depending on the particular product was as follows:

*1.2.1. Dimethyl N-benzylamino(pyren-1-yl)methylphosphonate (**3Aa**)*

The crude reaction mixture was dissolved in dichloromethane and washed three times with a saturated aqueous solution of sodium bicarbonate. The organic layer was dried and solvent was removed in vacuo and the residual oil was purified by column chromatography on silica gel with chloroform as eluent. The oil product was triturated with diethyl ether until the precipitation of a product. The procedure gave 163 mg of **2Aa** (38% based on imine) as a white solid, m.p. 104-105°C. ^{1}H NMR ($CDCl_3$, 600 MHz): δ 8.43-8.41 (m, H_{pyr}, 1H); 8.28 (d, $^3J_{HH}$ = 8.0 Hz, H_{pyr}, 1H); 8.22-8.20 (m, H_{pyr}, 3H); 8.12-8.09 (m, H_{pyr}, 3H); 8.03 (t, $^3J_{HH}$ = 7.6 Hz, H_{pyr}, 1H); 7.30-7.25 (m, PhH, 3H); 7.21-7.19 (m, PhH, 2H); 5.24 (d, $^2J_{PH}$ = 20.6 Hz, CHP, 1H); 3.83 (d, $^2J_{HH}$ = 12.7 Hz, CH_2Ph, 1H); 3.76 (d, $^3J_{PH}$ = 10.6 Hz, $POCH_3$, 3H); 3.57 (d, $^2J_{HH}$ = 12.7 Hz, CH_2Ph, 1H); 3.36 (d, $^3J_{PH}$ = 10.4 Hz, $POCH_3$, 3H). ^{13}C NMR (150 MHz, $CDCl_3$): δ 131.6 (C_{pyr}); 131.2 (d, $^4J_{CP}$ = 2.8 Hz, C_{pyr}); 130.1 (d, $^3J_{CP}$ = 7.1 Hz, C_{pyr}); 128.8 (C_{Ph}); 128.6 (C_{Ph}); 128.0 (C_{pyr}); 127.9 (C_{pyr}); 127.7 (C_{pyr}); 127.5 (C_{Ph}); 126.3 (C_{pyr}); 126.0 (C_{pyr}); 125.6 (C_{pyr}); 125.5 (d, $^4J_{CP}$ = 2.9 Hz, C_{pyr}); 125.3 (C_{pyr}); 125.2 (d, $^4J_{CP}$ = 1.6 Hz, C_{pyr}); 125.0 (C_{pyr}); 122.7 (C_{pyr}); 54.0 (d, $^2J_{CP}$ = 7.1 Hz, PO*C*); 53.7 (d, $^2J_{CP}$ = 7.2 Hz, PO*C*); 52.1 (d, $^3J_{CP}$ = 17.1 Hz, PCN*C*). ^{31}P NMR (243 MHz, $CDCl_3$): δ 25.88. IR **(**KBr**)**: 3433 (νNH); 3308 (νCH); 2953 (CH_2); 1603, 1480, 1449 (C=C); 1240 (P=O);1065; 1039 (P-O); 829; 742; 616.

Anal. Calcd for $C_{26}H_{24}NO_3P x ^1/_{20} CHCl_3$: C, 71.86; H, 5.57; N, 3.22. Found: C, 72.04, H, 5.54, N, 3.18.

*1.2.2. General procedure purification of phosphonates **3Ab–Ah***

The crude reaction mixture was dissolved in dichloromethane and washed three times with a sodium bicarbonate saturated water solution. Organic layer was dried and solvent was evaporated in vacuo yielding crude aminophosphonate as a yellow solid or yellow oil which was further purified by column chromatography on silica gel with chloroform as eluent.

*1.2.2.1. Dimethyl N-phenylamino(pyren-1-yl)methylphosphonate (**3Ab**)*

Y = (430 mg, 97%), white solid, m.p. 203-204°C. ^{1}H NMR ($CDCl_3$, 600 MHz): δ 8.49 (d, $^3J_{HH}$ = 9.4 Hz, H_{pyr}, 1H); 8.30 (dd, $^3J_{HH}$ = 8.1 and $^4J_{HH}$ = 2.5 Hz, H_{pyr}, 1H); 8.26-8.24 (m, H_{pyr}, 2H); 8.22 (d, $^3J_{HH}$ = 7.4 Hz, H_{pyr}, 1H); 8.17 (d, $^3J_{HH}$ = 8.1 Hz, H_{pyr}, 1H); 8.08 (d, $^3J_{HH}$ = 8.8 Hz, H_{pyr}, 1H); 8.06-8.03 (m, H_{pyr}, 2H); 7.00 (dd, $^3J_{HH}$ = 7.4 and 8.6 Hz, PhH$_m$, 2H); 6.62 (t, $^3J_{HH}$ = 7.4 Hz, PhH$_p$, 1H); 6.59 (dd, $^3J_{HH}$ = 8.6 Hz and $^4J_{HH}$ = 0.8 Hz, PhH$_o$, 2H); 5.96 (d, $^2J_{PH}$ = 23.9 Hz, CHP, 1H); 5.11 (broad s, NH, 1H); 3.85 (d, $^3J_{PH}$ = 10.7 Hz, $POCH_3$, 3H); 3.12 (d, $^3J_{PH}$ = 10.6 Hz, $POCH_3$, 3H). ^{13}C NMR (150 MHz, $CDCl_3$): δ 131.6 (C_{pyr}); 131.3 (d, $^4J_{CP}$ = 2.9 Hz, C_{pyr}); 130.9 (C_{pyr}); 129.4 (C_{Ph}); 129.2 (d, $^3J_{CP}$ = 6.5 Hz, C_{pyr}); 128.5 (C_{pyr}); 127.8 (C_{pyr}); 127.7 (C_{pyr}); 126.3 (C_{pyr}); 125.8 (C_{pyr}); 125.6 (d, $^4J_{CP}$ = 3.1 Hz, C_{pyr}); 125.5 (C_{pyr}); 125.4 (C_{pyr}); 125.2 (d, $^4J_{CP}$ = 1.8 Hz, C_{pyr}); 125.0 (C_{pyr}); 122.1 (C_{pyr}); 118.8 (C_{Ph}); 114.1 (C_{Ph}); 54.0 (d, $^2J_{CP}$ = 6.9 Hz, PO*C*); 52.1 (d, $^1J_{CP}$ = 151.1 Hz, P*C*). ^{31}P NMR (243 MHz, $CDCl_3$): δ 25.14. IR (KBr): 3424 (νNH); 3292 (νCH); 2949 (CH); 1604, 1497 (C=C); 1238 (P=O);1061; 1027 (P-O); 847; 757; 616. Anal. Calcd for $C_{25}H_{22}NO_3P x ^1/_8 CHCl_3$: C, 70.12; H, 5.18; N, 3.25. Found: C, 70.17, H, 5.24, N, 3.18.

*1.2.2.2. Dimethyl N-(4-methylphenyl)amino(pyren-1-yl)methylphosphonate (**3Ac**)*

Y = (343 mg, 80%), white solid, m.p: 163-164°C. ^{1}H NMR ($CDCl_3$, 600 MHz): δ 8.49 (d, $^3J_{HH}$ = 9.2 Hz, H_{pyr}, 1H); 8.30 (dd, $^3J_{HH}$ = 8.0 and $^4J_{HH}$ = 2.5 Hz, H_{pyr}, 1H); 8.25-8.21 (m, H_{pyr}, 3H); 8.16 (d, $^3J_{HH}$ = 7.4 Hz, H_{pyr}, 1H); 8.08 (d, $^3J_{HH}$ = 8.9 Hz, H_{pyr}, 1H); 8.04-8.02 (m, H_{pyr}, 2H); 6.80 (app. d, $^3J_{HH}$ = 8.4 Hz, *p*-C_6H_4, 2H); 6.50 (app. d, $^3J_{HH}$ = 8.9 Hz, *p*-C_6H_4, 2H); 5.93 (d, $^2J_{PH}$ = 24.0 Hz, CHP, 1H); 3.84 (d, $^3J_{PH}$ = 10.6 Hz, $POCH_3$, 3H); 3.13 (d, $^3J_{PH}$ = 10.5 Hz, $POCH_3$, 3H); 2.01 (s, CH_3, 3H). ^{13}C NMR (150 MHz, $CDCl_3$): δ 131.6 (C_{pyr}); 131.3 (d, $^4J_{CP}$ = 2.0 Hz, C_{pyr}); 130.9 (C_{pyr}); 129.9 (C_{Ph}); 129.3 (d, $^3J_{CP}$ = 6.5 Hz, C_{pyr}); 129.2 (C_{pyr}); 128.4 (C_{Ph}); 127.8 (C_{pyr}); 127.7 (C_{pyr}); 126.5 (C_{pyr}); 126.3 (C_{pyr}); 125.9 (C_{pyr}); 125.8 (C_{pyr}); 125.6 (d, $^4J_{CP}$ = 3.1 Hz, C_{pyr}); 125.5 (C_{pyr}); 125.4 (C_{pyr}); 125.2 (d, $^4J_{CP}$ = 1.8 Hz, C_{pyr}); 125.0 (C_{pyr}); 124.7 (C_{pyr}); 124.6 (C_{pyrr}); 124.4 (C_{pyr}); 122.2 (C_{pyr}); 121.5 (C_{pyr}); 114.2 (C_{Ph}); 54.0 (d, $^2J_{CP}$ = 15.6 Hz, PO*C*); 53.9 (d, $^2J_{CP}$ = 15.4 Hz, PO*C*); 52.3 (d, $^1J_{CP}$ = 152.0 Hz, P*C*); 20.5 (C_{Ar}-C). ^{31}P NMR (243 MHz, $CDCl_3$): δ 25.25. IR (KBr): 3432 (νNH); 3310 (νCH); 2948 (CH_3); 1616, 1522, 1458 (C=C); 1236 (P=O);1063; 1036 (P-O); 850; 826; 615. Anal. Calcd for $C_{26}H_{24}NO_3P$: C, 72.72; H, 5.63; N, 3.26. Found: C, 72.46, H, 5.61, N, 3.15.

*1.2.2.3. Dimethyl N-(4-methoxyphenyl)amino(pyren-1-yl)methylphosphonate (**3Ad**)*

Y = (227 mg, 51%), white solid, m.p.184°C. ^{1}H NMR ($CDCl_3$, 600 MHz): δ 8.48 (d, $^3J_{HH}$ = 9.4 Hz, H_{pyr}, 1H); 8.30-8.29 (m, H_{pyr}, 1H); 8.25-8.21 (m, H_{pyr}, 3H); 8.17 (d, $^3J_{HH}$ = 8.0 Hz, H_{pyr}, 1H); 8.08 (d, $^3J_{HH}$ = 8.8 Hz, H_{pyr}, 1H); 8.05-8.03 (m, H_{pyr}, 2H); 6.58 (part of AA'BB' system, $^3J_{HH}$ = 8.9 Hz, *p*-C_6H_4, 2H); 6.54 (part of AA'BB' system, $^3J_{HH}$ = 8.9 Hz, *p*-C_6H_4, 2H); 5.89 (d, $^2J_{PH}$ = 24.3 Hz, CHP, 1H); 4.87 (broad s, NH, 1H); 3.84 (d, $^3J_{PH}$ = 10.6 Hz, $POCH_3$, 3H); 3.60 (s, OCH_3, 3H); 3.15 (d, $^3J_{PH}$ = 10.6 Hz, $POCH_3$, 3H). ^{13}C NMR (150 MHz, $CDCl_3$): δ 153.0 (C_{Ph}); 140.3 (d, $^2J_{CP}$ = 15.4 Hz, C_{Ph}); 131.6 (C_{pyr}); 131.3 (d, $^4J_{CP}$ = 3.0 Hz, C_{pyr}); 130.8 (C_{pyr}); 129.6 (d, $^4J_{CP}$ = 3.0 Hz, C_{pyr}); 129.3 (d, $^3J_{CP}$ = 6.5 Hz, C_{pyr}); 128.4 (C_{pyr}); 127.8 (d, $^4J_{CP}$ = 1.3 Hz, C_{pyr}); 127.7 (C_{pyr}); 126.3 (C_{pyr}); 125.8 (C_{pyr}); 125.6 (d, $^4J_{CP}$ = 3.4 Hz, C_{pyr}); 125.5 (C_{pyr}); 125.4 (d, $^4J_{CP}$ = 4.6 Hz, C_{pyr}); 125.2 (d, $^4J_{CP}$ = 1.7 Hz, C_{pyr}); 125.0 (C_{pyr}); 124.2 (C_{pyr}); 122.2 (C_{pyr}); 115.3 (C_{Ph}); 115.0 (C_{Ph}); 113.8 (C_{Ph}); 55.7 (O*C*); 54.0 (d, $^2J_{CP}$ = 17.6 Hz, PO*C*); 53.9 (d, $^2J_{CP}$ = 16.9 Hz, PO*C*); 52.8 (d, $^1J_{CP}$ = 152.0 Hz, P*C*). ^{31}P NMR (243 MHz, $CDCl_3$): δ 25.26. IR (KBr): 3416 (νNH); 3292 (νCH); 2950 (CH_3); 1509, 1469 (C=C); 1239 (P=O);1053; 1023 (P-O); 820; 763; 617. Anal. Calcd for $C_{26}H_{24}NO_4P$x$^2/_9$ $CHCl_3$: C, 66.73; H, 5.17; N, 2.97. Found: C, 66.87, H, 5.15, N, 3.02.

*1.2.2.4. Dimethyl N-(1-butyl)amino(pyren-1-yl)methylphosphonate (**3Ae**)*

Y = (43mg, 11%), yellow oil. ^{1}H NMR ($CDCl_3$, 600 MHz): δ 8.42-8.37 (m, H_{pyr}, 2H); 8.25 (d, $^3J_{HH}$ = 8.0 Hz, H_{pyr}, 1H); 8.21 (d, $^3J_{HH}$ = 7.6 Hz, H_{pyr}, 2H); 8.16 (d, $^3J_{HH}$ = 9.3 Hz, H_{pyr}, 1H); 8.10-8.07 (m, H_{pyr}, 2H); 8.02 (dd, $^3J_{HH}$ = 7.5 and 7.0 Hz, H_{pyr}, 1H); 5.30 (d, $^2J_{PH}$ = 20.7 Hz, CHP, 1H); 3.77 (d, $^3J_{PH}$ = 10.6 Hz, $POCH_3$, 3H); 3.37 (d, $^3J_{PH}$ = 10.4 Hz, $POCH_3$, 3H); 2.54 (t, $^3J_{HH}$ = 7.1 Hz, N-CH_2, 2H); 1.49-1.46 (m, CH_2, 2H); 1.14-1.12 (m, CH_2, 2H); 0.83 (t, $^3J_{HH}$ = 7.4 Hz, CH_3, 3H). ^{13}C NMR (150 MHz, $CDCl_3$): δ 131.6 (C_{pyr}); 131.1 (d, $^4J_{CP}$ = 2.9 Hz, C_{pyr}); 130.9 (C_{pyr}); 130.0 (C_{pyr}); 129.9 (C_{pyr}); 128.1 (C_{pyr}); 127.8 (C_{pyr}); 127.7 (C_{pyr}); 126.2 (C_{pyr}); 125.8 (C_{pyr}); 125.6 (C_{pyr}); 125.4 (d, $^4J_{CP}$ = 3.1 Hz, C_{pyr}); 125.3 (C_{pyr}); 125.2 (d, $^4J_{CP}$ = 1.7 Hz, C_{pyr}); 125.0 (C_{pyr}); 122.7 (C_{pyr}); 53.9 (d, $^2J_{CP}$ = 6.8 Hz, PO*C*); 53.6 (d, $^2J_{CP}$ = 7.3 Hz, PO*C*); 48.0 (d, $^3J_{CP}$ = 14.6 Hz, PCN*C*); 32.3 (C_{aliph}); 20.5 (C_{aliph}); 14.1 (C_{aliph}). ^{31}P NMR (243 MHz, $CDCl_3$): δ 25.63. IR (KBr): 3442 (νNH); 2953 (νCH); 2928 (CH_2); 1603, 1458 (C=C); 1248 (P=O);1058; 1032 (P-O); 819; 764; 616. Anal. Calcd for $C_{23}H_{26}NO_3P$x$^1/_4$ $CHCl_3$: C, 65.66; H, 6.22; N, 3.29. Found: C, 65.41, H, 5.18, N, 3.27.

*1.2.2.5. Dimethyl N-(1-propyl)amino(pyren-1-yl)methylphosphonate (**3Af**)*

Y = (183 mg, 48%), yellow oil. ^{1}H NMR ($CDCl_3$, 600 MHz): δ 8.42-8.41 (m, H_{pyr}, 1H); 8.36 (dd, $^3J_{HH}$ = 8.0 and $^4J_{HH}$ = 2.0 Hz, H_{pyr}, 1H); 8.25 (d, $^3J_{HH}$ = 8.0 Hz, H_{pyr}, 2H); 8.20 (d, $^3J_{HH}$ = 7.6 Hz, H_{pyr}, 2H); 8.15 (d, $^3J_{HH}$ = 8.1 Hz, H_{pyr}, 1H); 8.10-8.06 (m, H_{pyr}, 2H); 8.02 (t, $^3J_{HH}$ = 7.6 Hz, H_{pyr}, 1H); 5.28 (d, $^2J_{PH}$ = 20.8 Hz, CHP, 1H); 3.77 (d, $^3J_{PH}$ = 10.6 Hz, $POCH_3$, 3H); 3.36 (d, $^3J_{PH}$ = 10.4 Hz, $POCH_3$, 3H); 2.52-2.48 (m, N-CH_2, 2H); 1.51-1.49 (m, CH_2, 2H); 0.85 (t, $^3J_{HH}$ = 7.4 Hz, CH_3, 3H). ^{13}C NMR (150 MHz, $CDCl_3$): δ 131.6 (C_{pyr}); 131.1 (d, $^4J_{CP}$ = 3.1 Hz, C_{pyr}); 130.9 (C_{pyr}); 130.0 (C_{pyr}); 129.9 (C_{pyr}); 128.1 (C_{pyr}); 127.8 (C_{pyr}); 127.7 (d, $^3J_{CP}$ = 1.5 Hz, C_{pyr}); 126.2 (C_{pyr}); 125.9 (C_{pyr}); 125.6 (C_{pyr}); 125.4 (d, $^4J_{CP}$ = 3.0 Hz, C_{pyr}); 125.3 (C_{pyr}); 125.1 (d, $^4J_{CP}$ = 1.7 Hz, C_{pyr}); 125.0 (C_{pyr}); 122.7 (C_{pyr}); 53.9 (d, $^2J_{CP}$ = 7.4 Hz, PO*C*);

53.6 (d, $^2J_{CP}$ = 6.7 Hz, PO*C*); 52.2 (d, $^3J_{CP}$ = 16.0 Hz, PCN*C*); 23.2 (C_{aliph}); 11.8 (C_{aliph}). ^{31}P NMR (243 MHz, $CDCl_3$): δ 25.90. IR (KBr): 3448 (νNH); 3305, 2949, 2922 (νCH); 2848 (CH_2); 1602, 1558, 1457 (C=C); 1251 (P=O);1061; 1034 (P-O); 853; 828; 752; 617. Anal. Calcd for $C_{22}H_{24}NO_3P$: C, 69.28; H, 6.34; N, 3.67. Found: C, 69.00, H, 6.52, N, 3.40.

*1.2.2.6. Dimethyl N-furfurylamino(pyren-1-yl)methylphosphonate (**3Ag**)*

Y = (256 mg ,61%), yellow oil. ^{1}H NMR ($CDCl_3$, 600 MHz): δ 8.44-8.43 (m, H_{pyr}, 1H); 8.27 (d, $^3J_{HH}$ = 8.0 Hz, H_{pyr}, 2H); 8.22-8.20 (m, H_{pyr}, 2H); 8.13 (d, $^3J_{HH}$ = 9.4 Hz, H_{pyr}, 1H); 8.11-8.08 (m, H_{pyr}, 2H); 8.03 (t, $^3J_{HH}$ = 7.6 Hz, H_{pyr}, 1H); 7.34 (m, H_{fur}, 1H); 6.27-6.26 (m, H_{fur}, 1H); 5.99-5.98 (m, H_{fur}, 1H); 5.28 (d, $^2J_{PH}$ = 19.6 Hz, CHP, 1H); 3.87 (d, $^2J_{HH}$ = 14.7 Hz, CH_2Fur, 1H); 3.70 (d, $^3J_{PH}$ = 10.6 Hz, $POCH_3$, 3H); 3.59 (d, $^2J_{HH}$ = 14.7 Hz, CH_2Fur, 1H); 3.42 (d, $^3J_{PH}$ = 10.4 Hz, $POCH_3$, 3H). ^{13}C NMR (150 MHz, $CDCl_3$): δ 153.2 (C_{fur}); 142.8 (C_{fur}); 132.0 (C_{pyr}); 131.7 (d, $^4J_{CP}$ = 2.6 Hz, C_{pyr}); 131.2 (C_{pyr}); 130.5 (d, $^3J_{CP}$ = 7.5 Hz, C_{pyr}); 129.2 (C_{pyr}); 128.4 (C_{pyr}); 128.3 (C_{pyr}); 128.1 (C_{pyr}); 126.7 (C_{pyr}); 126.6 (C_{pyr}); 126.0 (C_{pyr}); 125.8 (d, $^4J_{CP}$ = 3.0 Hz, C_{pyr}); 125.7 (C_{pyr}); 125.6 (d, $^4J_{CP}$ = 1.7 Hz, C_{pyr}); 125.4 (C_{pyr}); 123.2 (C_{pyr}); 110.7 (C_{fur}); 108.7 (C_{fur}); 54.3 (d, $^2J_{CP}$ = 7.4 Hz, PO*C*); 54.1 (d, $^2J_{CP}$ = 7.0 Hz, PO*C*); 44.3 (d, $^3J_{CP}$ = 17.3 Hz, PCN*C*). ^{31}P NMR (243 MHz, $CDCl_3$): δ 25.88. IR (KBr): 3442 (νNH); 3308 (νCH); 2957 (CH_2); 1604, 1587, 1510, 1446 (C=C); 1240 (P=O);1064; 1038 (P-O); 834; 741; 614. Anal. Calcd for $C_{24}H_{22}NO_4P$: C, 68.73; H, 5.29; N, 3.34. Found: C, 68.49, H, 5.34, N, 3.27.

*1.2.2.7. Dimethyl N-cyklohexylamino(pyren-1-yl)methylphosphonate (**3Ah**)*

Y = (193 mg, 46%) yellow oil. ^{1}H NMR ($CDCl_3$, 600 MHz): δ 8.39-8.34 (m, H_{pyr}, 2H); 8.25 (d, $^3J_{HH}$ = 7.9 Hz, H_{pyr}, 1H); 8.20 (d, $^3J_{HH}$ = 7.6 Hz, H_{pyr}, 2H); 8.16 (d, $^3J_{HH}$ = 9.2 Hz, H_{pyr}, 1H); 8.10-8.07 (m, H_{pyr}, 2H); 8.02 (t, $^3J_{HH}$ = 7.6 Hz, H_{pyr}, 1H); 5.45 (d, $^2J_{PH}$ = 23.3 Hz, CHP, 1H); 3.82 (d, $^3J_{PH}$ = 10.5 Hz, $POCH_3$, 3H); 3.29 (d, $^3J_{PH}$ = 10.4 Hz, $POCH_3$, 3H); 2.39-2.32 (m, *c*-Hex, 1H); 1.94-1.92 (m, *c*-Hex, 1H); 1.66-1.60 (m, *c*-Hex, 3H); 1.48-1.45 (m, *c*-Hex, 1H); 1.18-0.94 (m, *c*-Hex, 5H). ^{13}C NMR (150 MHz, $CDCl_3$): δ 131.6 (C_{pyr}); 131.0 (d, $^4J_{CP}$ = 2.5 Hz, C_{pyr}); 130.9 (C_{pyr}); 130.6 (C_{pyr}); 129.7 (d, $^3J_{CP}$ = 7.5 Hz, C_{pyr}); 128.1 (C_{pyr}); 127.7 (C_{pyr}); 126.2 (C_{pyr}); 126.0 (C_{pyr}); 125.6 (C_{pyr}); 125.4 (d, $^4J_{CP}$ = 3.0 Hz, C_{pyr}); 125.2 (C_{pyr}); 125.1 (C_{pyr}); 125.0 (C_{pyr}); 122.6 (C_{pyr}); 54.2 (d, $^2J_{CP}$ = 6.8 Hz, PO*C*); 54.0 (d, $^3J_{CP}$ = 15.5 Hz, PCN*C*); 53.5 (d, $^2J_{CP}$ = 6.9 Hz, PO*C*); 34.5 (C_{aliph}); 32.6 (C_{aliph}); 26.2 (C_{aliph}); 25.0 (C_{aliph}); 24.5 (C_{aliph}). ^{31}P NMR (243 MHz, $CDCl_3$): δ 26.44. IR (KBr): 3443 (νNH); 3300 (νCH); 2927 (CH_2); 1597, 1591, 1511, 1445 (C=C); 1245 (P=O);1062; 1021 (P-O); 846; 818; 764; 724; 612. Anal. Calctd for $C_{25}H_{28}NO_3P$: C, 71.24; H, 6.70; N, 3.32. Found: C, 70.97, H, 6.88, N, 3.31.

*1.2.3. General procedure purification of phosphonates **3Ba–Be***

The crude reaction mixture was dissolved in a minimum amount of diethyl ether and triturated until formed a yellow precipitate. This operation was repeated several times. Crude aminophosphonate was purified by column chromatography on silica gel with chloroform as eluent.

*1.2.3.1. Diethyl N-benzylamino(pyren-1-yl)methylphosphonate (**3Ba**)*

Y = (115 mg, 25%), white solid, m.p.124°C. ^{1}H NMR ($CDCl_3$, 600 MHz): δ 8.43-8.41 (m, H_{pyr}, 1H); 8.27 (d, $^3J_{HH}$ = 7.9 Hz, H_{pyr}, 1H); 8.22-8.19 (m, H_{pyr}, 3H); 8.10-8.07 (m, H_{pyr}, 3H); 8.02 (t, $^3J_{HH}$ = 7.6 Hz, H_{pyr}, 1H); 7.29-7.25 (m, PhH, 3H); 7.20-7.19 (m, PhH, 2H); 5.21 (d, $^2J_{PH}$ = 20.8 Hz, CHP, 1H); 4.18-4.11 (m, $PO\underline{CH_2}CH_3$, 1H); 4.09-4.03 (m, $PO\underline{CH_2}CH_3$, NH, 2H); 3.87-3.82 (m, $PO\underline{CH_2}CH_3$, 1H); 3.83 (d, $^2J_{HH}$ = 13.2 Hz, CH_2Ph, 1H); 3.63-3.67 (m, $PO\underline{CH_2}CH_3$, 1H); 3.56 (d, $^2J_{HH}$ = 13.2 Hz, CH_2Ph, 1H); 1.27 (t, $^3J_{HH}$ = 7.0 Hz, $POCH_2\underline{CH_3}$, 3H); 0.91 (t, $^3J_{HH}$ = 7.0 Hz, $POCH_2\underline{CH_3}$, 3H). ^{13}C NMR (150 MHz, $CDCl_3$): δ 131.6 (C_{pyr}); 131.2 (C_{pyr}); 130.9 (C_{pyr}); 130.2 (d, $^3J_{CP}$ = 7.1 Hz, C_{pyr}); 128.8 (C_{pyr}); 128.6 (C_{pyr}); 127.8 (C_{pyr}); 127.7 (C_{pyr}); 127.4 (C_{pyr}); 126.2 (C_{pyr}); 125.5 (C_{pyr}); 125.4 (d, $^4J_{CP}$ = 2.8 Hz, C_{pyr}); 125.3 (C_{pyr}); 125.1 (C_{pyr}); 125.0 (C_{pyr}); 123.0 (C_{Ph}); 63.3 (d, $^2J_{CP}$ = 7.0 Hz, PO*C*); 63.1 (d, $^2J_{CP}$ = 6.8 Hz, PO*C*); 51.5 (d, $^3J_{CP}$ = 16.6 Hz, PCN*C*); 16.7 (d, $^2J_{CP}$ = 5.6 Hz, POC*C*); 16.3 (d, $^2J_{CP}$ = 5.4 Hz, POC*C*). ^{31}P NMR (243 MHz, $CDCl_3$): δ 23.49. IR (KBr): 3432 (νNH); 3273 (νCH); 2975 (CH_2); 1605, 1496, 1453 (C=C); 1244 (P=O);1059; 1024 (P-O); 971; 841; 738. Anal. Calcd for $C_{28}H_{28}NO_3P$: C, 73.51; H, 6.17; N, 3.06. Found: C, 73.18, H, 6.05, N, 3.02.

*1.2.3.2. Diethyl N-phenylamino(pyren-1-yl)methylphosphonate (**3Bb**)*

Y = (221mg, 50%), white solid, m.p. 200°C; Lit [1] 155–157°C. ^{1}H NMR ($CDCl_3$, 600 MHz): δ 8.52 (d, $^3J_{HH}$ = 9.3 Hz, H_{pyr}, 1H); 8.29 (dd, $^3J_{HH}$ = 8.0 and $^4J_{HH}$ = 2.6 Hz, H_{pyr}, 1H); 8.25-8.21 (m, H_{pyr}, 3H); 8.16 (d, $^3J_{HH}$ = 8.0 Hz, H_{pyr}, 1H); 8.07 (d, $^3J_{HH}$ = 8.9 Hz, H_{pyr}, 1H); 8.05-8.02 (m, H_{pyr}, 2H); 7.00 (ddd, $^3J_{HH}$ = 7.4 and 8.6 Hz and $^4J_{HH}$ = 1.9 Hz, PhH_m, 2H); 6.62 (tt, $^3J_{HH}$ = 7.4 Hz and $^4J_{HH}$ = 1.0 Hz, PhH_p, 1H); 6.58 (dd, $^3J_{HH}$ = 8.6 Hz and $^4J_{HH}$ = 0.8 Hz, PhH_o, 2H); 5.92 (dd, $^2J_{PH}$ = 24.2 Hz and $^3J_{HH}$ = 6.2 Hz, CHP, 1H); 5.20-5.25 (m, NH, 1H); 4.20 (dq, $^3J_{HH}$ = 7.1 Hz and $^3J_{PH}$ = 7.8 Hz, $PO\underline{CH_2}CH_3$, 2H); 3.74-3.67 (m, $PO\underline{CH_2}CH_3$, 1H); 3.27-3.20 (m, $PO\underline{CH_2}CH_3$, 1H); 1.35 (t, $^3J_{HH}$ = 7.1 Hz, $POCH_2\underline{CH_3}$, 3H); 0.74 (t, $^3J_{HH}$ = 7.1 Hz, $POCH_2\underline{CH_3}$, 3H). ^{13}C NMR (150 MHz, $CDCl_3$): δ 146.5 (C_{Ar}); 146.4 (C_{Ar}); 131.6 (C_{pyr}); 131.2 (d, $^4J_{CP}$ = 3.1 Hz, C_{pyr}); 130.9 (C_{pyr}); 129.9 (d, $^4J_{CP}$ = 3.2 Hz, C_{pyr}); 129.4 (C_{pyr}); 129.3 (C_{Ar}); 128.2 (C_{pyr}); 127.8 (d, $^3J_{CP}$ = 6.2 Hz, C_{pyr}); 126.3 (C_{pyr}); 125.5 (d, $^4J_{CP}$ = 3.2 Hz, C_{pyr}); 125.4 (C_{pyr}); 125.3 (d, $^3J_{CP}$ = 4.6 Hz, C_{pyr}); 125.2 (d, $^4J_{CP}$ = 1.7 Hz, C_{pyr}); 125.0 (C_{pyr}); 122.5 (d, $^4J_{CP}$ = 1.4 Hz, C_{Ph}); 118.6 (C_{Ph}); 114.0 (C_{Ph}); 63.6 (d, $^2J_{CP}$ = 6.8 Hz, PO*C*); 63.5 (d, $^2J_{CP}$ = 7.0 Hz, PO*C*); 52.4 (d, $^1J_{CP}$ = 151.0 Hz, P*C*); 16.7 (d, $^2J_{CP}$ = 5.6 Hz, POC*C*); 16.1 (d, $^2J_{CP}$ = 5.5 Hz, POC*C*). ^{31}P NMR (243 MHz, $CDCl_3$): δ 22.75. IR (KBr): 3423 (νNH); 3297 (νCH); 2980 (CH); 1604, 1498 (C=C); 1233 (P=O);1059; 1028 (P-O); 969; 845; 617. Anal. Calcd for $C_{27}H_{26}NO_3P$: C, 73.12; H, 5.91; N, 3.16. Found: C, 72.86, H, 5.82, N, 3.11.

*1.2.3.3. Diethyl N-(4-methylphenyl)amino(pyren-1-yl)methylphosphonate (**3Bc**)*

Y = (178 mg, 39%), white solid, m.p. 189-190°C (186°C); Lit [1] 159–161°C. ^{1}H NMR ($CDCl_3$, 600 MHz): δ 8.51 (d, $^3J_{HH}$ = 9.3 Hz, H_{pyr}, 1H); 8.29 (dd, $^3J_{HH}$ = 8.1 and $^4J_{HH}$ = 2.6 Hz, H_{pyr}, 1H); 8.24-8.20 (m, H_{pyr}, 3H); 8.15 (d, $^3J_{HH}$ = 8.0 Hz, H_{pyr}, 1H); 8.07 (d, $^3J_{HH}$ = 8.8 Hz, H_{pyr}, 1H); 8.04-8.02 (m, H_{pyr}, 2H); 6.80 (app. d, $^3J_{HH}$ = 8.3 Hz, *p*-C_6H_4, 2H); 6.50 (app. d, $^3J_{HH}$ = 8.6 Hz, *p*-C_6H_4, 2H); 5.90 (d, $^2J_{PH}$ = 24.1 Hz, CHP, 1H); 5.17 (broad s, NH, 1H); 4.20 (dq,

$^3J_{HH}$ = 7.1 Hz and $^3J_{PH}$ = 7.6 Hz, $PO\underline{CH_2}CH_3$, 2H); 3.74-3.68 (m, $PO\underline{CH_2}CH_3$, 1H); 3.28-3.22 (m, $PO\underline{CH_2}CH_3$, 1H); 2.11 (s, CH_3, 3H); 1.34 (t, $^3J_{HH}$ = 7.1 Hz, $POCH_2\underline{CH_3}$, 3H); 0.75 (t, $^3J_{HH}$ = 7.1 Hz, $POCH_2\underline{CH_3}$, 3H). ^{13}C NMR (150 MHz, $CDCl_3$): δ 144.2 (d, $^4J_{CP}$ = 15.2 Hz, C_{pyr}); 131.6 (C_{pyr}); 131.2 (d, $^4J_{CP}$ = 3.0 Hz, C_{pyr}); 130.9 (C_{pyr}); 130.1 (C_{pyr}); 129.9 (C_{pyr}); 129.4 (d, $^3J_{CP}$ = 6.2 Hz, C_{pyr}); 128.1 (C_{pyr}); 127.8 (C_{pyr}); 127.8 (C_{pyr}); 127.7 (C_{pyr}); 126.2 (C_{pyr}); 125.7 (C_{pyr}); 125.6 (d, $^4J_{CP}$ = 2.8 Hz, C_{pyr}); 125.4 (C_{pyr}); 125.3 (C_{pyr}); 125.2 (C_{pyr}); 125.0 (C_{pyr}); 122.6 (C_{Ph}); 114.1 (C_{Ph}); 63.5 (d, $^2J_{CP}$ = 6.9 Hz, PO*C*); 52.7 (d, $^1J_{CP}$ = 151.4 Hz, P*C*); 20.5 (C_{Ar}-C); 16.7 (d, $^2J_{CP}$ = 5.6 Hz, POC*C*); 16.2 (d, $^2J_{CP}$ = 5.4 Hz, POC*C*). ^{31}P NMR (243 MHz, $CDCl_3$): δ 22.85. IR (KBr): 3432 (νNH); 3304 (νCH); 2978 (CH_3); 1618, 1523 (C=C); 1229 (P=O);1057; 1026 (P-O); 978; 849; 616. Anal. Calcd for $C_{28}H_{28}NO_3P$ $^1/_6$ $CHCl_3$: C, 70.86; H, 5.95; N, 2.93. Found: C, 70.74, H, 5.88, N, 2.84.

*1.2.3.4. Diethyl N-(4-methoxyphenyl)amino(pyren-1-yl)methylphosphonate (**3Bd**)*

Y = (184 mg, 39%), white solid, m.p. 158°C; Lit [1] 168–170°C. ^{1}H NMR ($CDCl_3$, 600 MHz): δ 8.51 (d, $^3J_{HH}$ = 9.3 Hz, H_{pyr}, 1H); 8.31-8.29 (m, H_{pyr}, 1H); 8.24-8.21 (m, H_{pyr}, 3H); 8.16 (d, $^3J_{HH}$ = 8.0 Hz, H_{pyr}, 1H); 8.07 (d, $^3J_{HH}$ = 8.8 Hz, H_{pyr}, 1H); 8.05-8.02 (m, H_{pyr}, 2H); 6.59-6.57 (m, part of AA'BB' system, *p*-C_6H_4, 2H); 6.55-6.53 (m, part of AA'BB' system, *p*-C_6H_4, 2H); 5.86 (d, $^2J_{PH}$ = 23.8 Hz, CHP, 1H); 4.23-4.18 (m, $PO\underline{CH_2}CH_3$, 2H); 3.75-3.71 (m, $PO\underline{CH_2}CH_3$, 1H); 3.59 (s, OCH_3, 3H); 3.31-3.27 (m, $PO\underline{CH_2}CH_3$, 1H); 1.34 (t, $^3J_{HH}$ = 7.0 Hz, $POCH_2\underline{CH_3}$, 3H); 0.76 (t, $^3J_{HH}$ = 7.8 Hz, $POCH_2\underline{CH_3}$, 3H). ^{13}C NMR (150 MHz, $CDCl_3$): δ 152.9 (C_{pyr}); 140.5 (d, $^2J_{CP}$ = 15.4 Hz, C_{pyr}); 131.6 (C_{pyr}); 131.2 (d, $^4J_{CP}$ = 3.0 Hz, C_{pyr}); 130.9 (C_{pyr}); 130.0 (C_{pyr}); 129.4 (d, $^3J_{CP}$ = 6.4 Hz, C_{pyr}); 128.2 (C_{pyr}); 127.8 (C_{pyr}); 127.7 (C_{pyr}); 126.2 (C_{pyr}); 125.7 (C_{pyr}); 125.5 (d, $^4J_{CP}$ = 3.2 Hz, C_{pyr}); 125.4 (C_{pyr}); 125.2 (d, $^4J_{CP}$ = 1.9 Hz, C_{pyr}); 125.0 (C_{pyr}); 122.6 (C_{Ph}); 115.3 (C_{Ph}); 115.0 (C_{Ph}); 63.5 (d, $^2J_{CP}$ = 6.8 Hz, PO*C*); 63.5 (d, $^2J_{CP}$ = 6.7 Hz, PO*C*); 55.8 (O*C*); 53.2 (d, $^1J_{CP}$ = 151.5 Hz, P*C*); 16.7 (d, $^2J_{CP}$ = 5.8 Hz, POC*C*); 16.2 (d, $^2J_{CP}$ = 5.4 Hz, POC*C*). ^{31}P NMR (243 MHz, $CDCl_3$): δ 22.78. IR (KBr): 3432 (νNH); 3300 (νCH); 2979 (CH_3); 1511, 1440 (C=C); 1231 (P=O);1057; 1027 (P-O); 973; 818; 620. Anal. Calcd for $C_{28}H_{28}NO_4P$: C, 71.02; H, 5.96; N, 2.96. Found: C, 71.25, H, 5.86, N, 2.88.

*1.2.3.5. Diethyl N-(1-butyl)amino(pyren-1-yl)methylphosphonate (**3Be**)*

Y = (237 mg, 56%), yellow solid, m.p.82-83°C. ^{1}H NMR ($CDCl_3$, 600 MHz): δ 8.44 (d, $^3J_{HH}$ = 8.2 Hz, H_{pyr}, 1H); 8.38 (d, $^3J_{HH}$ = 7.7 Hz, H_{pyr}, 1H); 8.24 (d, $^3J_{HH}$ = 8.0 Hz, H_{pyr}, 1H); 8.20 (d, $^3J_{HH}$ = 7.6 Hz, H_{pyr}, 2H); 8.14 (d, $^3J_{HH}$ = 9.3 Hz, H_{pyr}, 1H); 8.10-8.06 (m, H_{pyr}, 2H); 8.02 (t, $^3J_{HH}$ = 7.6 Hz, H_{pyr}, 1H); 5.26 (d, $^2J_{PH}$ = 20.5 Hz, CHP, 1H); 4.19-4.12 (m, $POCH_2$, 1H); 4.11-4.04 (m, $POCH_2$, 1H); 3.89-3.83 (m, $POCH_2$, 1H); 3.66-3.59 (m, $POCH_2$, 1H); 2.54 (t, $^3J_{HH}$ = 7.1 Hz, N-CH_2, 2H); 1.51-1.46 (m, CH_2, 2H); 1.34-1.23 (m, CH_2, 2H); 1.27 (t, $^3J_{HH}$ = 7.1 Hz, $POCH_2\mathit{CH_3}$, 3H); 0.93 (t, $^3J_{HH}$ = 7.1 Hz, $POCH_2\mathit{CH_3}$, 3H); 0.83 (t, $^3J_{HH}$ = 7.4 Hz, CH_3, 3H). ^{13}C NMR (150 MHz, $CDCl_3$): δ 131.6 (C_{pyr}); 131.0 (d, $^4J_{CP}$ = 2.6 Hz, C_{pyr}); 130.9 (C_{pyr}); 130.4 (C_{pyr}); 130.0 (d, $^4J_{CP}$ = 4.5 Hz, C_{pyr}); 127.8 (C_{pyr}); 127.7 (C_{pyr}); 127.6 (C_{pyr}); 126.1 (C_{pyr}); 126.0 (C_{pyr}); 125.5 (C_{pyr}); 125.3 (d, $^4J_{CP}$ = 3.1 Hz, C_{pyr}); 125.2 (C_{pyr}); 125.1 (d, $^4J_{CP}$ = 1.7 Hz, C_{pyr}); 125.0 (C_{pyr}); 123.1 (C_{pyr}); 63.2 (d, $^2J_{CP}$ = 6.9 Hz, PO*C*); 63.0 (d, $^2J_{CP}$ = 7.0 Hz, PO*C*);

56.8 (d, $^1J_{CP}$ = 155.0 Hz, P*C*); 48.1 (d, $^3J_{CP}$ = 16.3 Hz, PCN*C*); 32.3 (C_{aliph}); 20.5 (C_{aliph}); 16.7 (d, $^2J_{CP}$ = 5.9 Hz, POC*C*); 16.3 (d, $^2J_{CP}$ = 5.5 Hz, POC*C*); 14.1 (C_{aliph}). ^{31}P NMR (243 MHz, $CDCl_3$): δ 23.34. IR (KBr): 3432 (νNH); 3311; 2956 (νCH); 2928 (CH_2); 1588, 1472, 1458 (C=C); 1237 (P=O);1062; 1027 (P-O); 969; 847; 787; 617. Anal. Calcd for $C_{25}H_{30}NO_3P$: C, 70.90; H, 7.14; N, 3.31. Found: C, 70.89; H, 7.23; N, 3.34.

*1.2.4. General procedure for the purification of phosphonates **3Ca–Cd and 3Cg***

The crude reaction mixture was dissolved in a small amount of pyridine. Next, elemental iodine was added in small portions and if the solution was discolored within 10 min, further portions of iodine were added. If after the addition, the mixture remained brown, it was stirred for another 10 min with heating on a water bath. Next the mixture was dissolved in methylene chloride and washed once with 50 ml of saturated aqueous sodium thiosulfate and three times with 50 ml of saturated aqueous sodium bicarbonate. The organic layer was separated and dried over sodium sulfate. The solvent was removed in vacuo. The obtained crude product was purified by chromatography on silica gel using chloroform as eluent.

*1.2.4.1. Dibenzyl N-benzylamino(pyren-1-yl)methylphosphonate (**3Ca**)*

Y = (308 mg, 53%), white solid, m.p. 100-101°C. ^{1}H NMR ($CDCl_3$, 600 MHz): δ 8.49-8.42 (m, H_{pyr}, 1H); 8.24 (d, $^3J_{HH}$ = 8.0 Hz, H_{pyr}, 1H); 8.22 (d, $^3J_{HH}$ = 7.5 Hz, H_{pyr}, 1H); 8.17 (d, H_{pyr}, $^3J_{HH}$ = 7.4 Hz, 1H); 8.12-8.08 (m, H_{pyr}, 3H); 8.03 (t, $^3J_{HH}$ = 7.5 Hz, H_{pyr}, 1H); 7.97 (d, $^3J_{HH}$ = 9.2 Hz, H_{pyr}, 1H); 7.37-7.36 (m, H_{Ph}, 1H); 7.27-7.25 (m, H_{Ph}, 4H); 7.25-7.23 (m, H_{Ph}, 3H); 7.17-7.15 (m, H_{Ph}, 2H); 6.99 (app. t, $^3J_{HH}$ = 7.4 Hz, H_{Ph}, 1H); 6.92 (ddd, $^3J_{HH}^{(1)}$ = $^3J_{HH}^{(2)}$ = 7.8 and $^4J_{HH}$ = 1.6 Hz, H_{Ph}, 2H); 6.81 (app. d, $^3J_{HH}$ = 7.4 Hz, H_{Ph}, 2H); 5.28 (d, $^2J_{PH}$ = 20.8 Hz, CHP, 1H); 5.13 and 5.00 (Part of AMX system, $^2J_{HH}$ = 11.6 Hz and $^3J_{PH}$ = 8.8 and 7.0 Hz, $POCH_2$, 2H); 4.65 and 4.22 (Part of AMX system, $^2J_{HH}$ = 11.6 Hz and $^3J_{PH}$ = 7.4 and 8.5 Hz, $POCH_2$, 2H); 3.87 (d, $^3J_{HH}$ = 13.2 Hz, NCH_2, 1H); 3.57 (d, $^3J_{HH}$ = 13.2 Hz, NCH_2, 1H). ^{13}C NMR (150 MHz, $CDCl_3$): δ 139.3 (C_{Ar}); 136.6 (d, $^3J_{CP}$ = 6.3 Hz, C_{pyr}); 136.0 (d, $^3J_{CP}$ = 5.7 Hz, C_{pyr}); 131.6 (C_{pyr}); 131.2 (d, $^4J_{CP}$ = 3.0 Hz, C_{pyr}); 130.9 (C_{pyr}); 130.2 (d, $^3J_{CP}$ = 7.6 Hz, C_{pyr}); 128.7 (C_{Ar}); 128.6 (d, $^3J_{CP}$ = 4.7 Hz, C_{Ar}); 128.4 (C_{pyr}); 128.2 (d, $^3J_{CP}$ = 6.6 Hz, C_{Ar}); 128.0 (C_{Ar}); 127.9 (C_{Ar}); 127.8 (C_{Ayr}); 127.7 (C_{Ar}); 127.6 (C_{Ar}); 127.4 (C_{Ar}); 126.2 (C_{Ar}); 125.5 (C_{Ar}); 125.4 (d, $^4J_{CP}$ = 2.8 Hz, C_{Ar}); 125.3 (C_{pyr}); 125.2 (C_{pyr}); 125.0 (C_{pyr}); 123.0 (C_{Ph}); 68.7 (d, $^2J_{CP}$ = 7.0 Hz, PO*C*); 68.4 (d, $^2J_{CP}$ = 7.0 Hz, PO*C*); 51.6 (d, $^3J_{CP}$ = 17.3 Hz, PCC*C*). ^{31}P NMR (243 MHz, $CDCl_3$): δ 24.03. IR (KBr): 3432 (νNH); 3288 (νCH); 3038, 2936 (CH_3); 1604, 1496, 1453 (C=C); 1236 (P=O);1058; 1028 (P-O); 852; 820; 622. Anal. Calcd for $C_{38}H_{32}NO_3P$: C, 78.47; H, 5.55; N, 2.41. Found: C, 78.56; H, 5.67; N, 2.44.

*1.2.4.2. Dibenzyl N-phenylamino(pyren-1-yl)methylphosphonate (**3Cb**)*

Y = (346 mg, 61%), white solid, m.p.179-180°C. ^{1}H NMR ($CDCl_3$, 600 MHz): δ 8.38 (d, $^3J_{HH}$ = 9.3 Hz, H_{pyr}, 1H); 8.28 (dd, $^3J_{HH}$ = 8.0 and $^4J_{HH}$ = 2.6 Hz, H_{pyr}, 1H); 8.20 (t, H_{pyr}, $^3J_{HH}$ = 8.0 Hz, 2H); 8.12 (d, $^3J_{HH}$ = 8.0 Hz, H_{pyr}, 1H); 8.10 (d, $^3J_{HH}$ = 9.2 Hz, H_{pyr}, 1H); 8.07-8.06 (m, H_{pyr}, 1H); 8.04-8.01 (m, H_{pyr}, 2H); 7.30-7.29 (m, H_{Ph}, 5H); 6.94 (t, $^3J_{HH}$ = 7.4 Hz, H_{Ph}, 1H); 6.84 (dd, $^3J_{HH}^{(1)}$ = $^3J_{HH}^{(2)}$ = 7.8 Hz, H_{Ph}, 2H); 6.79 (app. d, $^3J_{HH}$ = 8.5 Hz, *p*-C_6H_4, 2H); 6.67

(d, $^3J_{HH}$ = 7.4 Hz, H_{Ph}, 2H); 6.46 (app. d, $^3J_{HH}$ = 8.5 Hz, *p*-C_6H_4, 2H); 5.92 (d, $^2J_{PH}$ = 24.1 Hz, CHP, 1H); 5.10-5.08 (m, $POCH_2$, 2H); 4.65 and 4.22 (Part of AMX system, $^2J_{HH}$ = 11.6 Hz and $^3J_{PH}$ = 7.8 and 8.5 Hz, $POCH_2$, 2H). ^{13}C NMR (150 MHz, $CDCl_3$): δ 136.2 (d, $^3J_{CP}$ = 5.5 Hz, C_{pyr}); 135.4 (d, $^3J_{CP}$ = 5.7 Hz, C_{pyr}); 131.6 (C_{pyr}); 131.3 (d, $^4J_{CP}$ = 3.1 Hz, C_{pyr}); 130.9 (C_{pyr}); 129.4 (C_{pyr}); 129.3 (C_{pyr}); 128.8 (C_{pyr}); 128.7 (C_{pyr}); 128.4 (C_{pyr}); 128.3 (C_{pyr}); 128.2 (C_{pyr}); 127.8 (C_{pyr}); 127.7 (C_{pyr}); 127.7 (d, $^4J_{CP}$ = 1.6 Hz, C_{pyr}); 126.2 (C_{pyr}); 125.6 (C_{pyr}); 125.5 (d, $^4J_{CP}$ = 3.4 Hz, C_{pyr}); 125.4 (C_{pyr}); 125.4 (C_{pyr}); 125.3 (C_{pyr}); 125.2 (d, $^4J_{CP}$ = 1.9 Hz, C_{pyr}); 125.0 (C_{Ar}); 122.4 (C_{Ph}); 118.7 (C_{Ph}); 114.1 (C_{Ph}); 68.9 (d, $^2J_{CP}$ = 6.6 Hz, PO*C*); 68.8 (d, $^2J_{CP}$ = 6.7 Hz, PO*C*); 52.7 (d, $^1J_{CP}$ = 151.2 Hz, P*C*). ^{31}P NMR (243 MHz, $CDCl_3$): δ 23.48. IR (KBr): 3311 (νCH); 3034; 2948 (CH_3); 1602, 1526, 1467 (C=C); 1242 (P=O);1081; 1019 (P-O); 846; 824; 624. Anal. Calcd for $C_{37}H_{30}NO_3P$ $^1/_4$ $CHCl_3$: C, 74.88; H, 5.10; N, 2.34. Found: C, 75.08, H, 5.45, N, 2.15.

*1.2.4.3. Dibenzyl N-(4-methylphenyl)amino(pyren-1-yl)methylphosphonate (**3Cc**)*

Y = (396 mg, 68%) white solid, m.p.180°C. ^{1}H NMR ($CDCl_3$, 600 MHz): δ 8.38 (d, $^3J_{HH}$ = 9.3 Hz, H_{pyr}, 1H); 8.28 (dd, $^3J_{HH}$ = 8.0 and $^4J_{HH}$ = 2.6 Hz, H_{pyr}, 1H); 8.20 (t, H_{pyr}, $^3J_{HH}$ = 7.6 Hz, 2H); 8.14-8.10 (m, H_{pyr}, 2H); 8.07 (d, $^3J_{HH}$ = 8.8 Hz, H_{pyr}, 1H); 8.04-8.01 (m, H_{pyr}, 2H); 7.30-7.29 (m, H_{Ph}, 5H); 6.98 (ddd, $^3J_{HH}$ = 8.6 and 7.4 and $^4J_{HH}$ = 1.9 Hz, H_{Ph}, 2H); 6.94 (dt, $^3J_{HH}$ = 7.4 and $^4J_{HH}$ = 1.2 Hz, H_{Ph}, 1H); 6.84 (ddd, $^3J_{HH}{}^{(1)}$ = $^3J_{HH}{}^{(2)}$ = 7.9 and $^4J_{HH}$ = 1.7 Hz, H_{Ph}, 2H); 6.67 (app. d, $^3J_{HH}$ = 7.4 Hz, H_{Ph}, 2H); 6.62 (dt, $^3J_{HH}$ = 7.4 and $^4J_{HH}$ = 0.9 Hz, H_{Ph}, 1H); 6.54 (dd, $^3J_{HH}$ = 8.5 $^4J_{HH}$ = 0.7 Hz,, H_{Ph}, 2H); 5.93 (d, $^2J_{PH}$ = 24.2 Hz, CHP, 1H); 5.10-5.08 (m, $POCH_2$, 2H); 4.65 and 4.23 (Part of AMX system, $^2J_{HH}$ = 11.6 Hz and $^3J_{PH}$ = 7.7 and 8.4 Hz, $POCH_2$, 1H); 2.10 (s, CH_3, 3H). ^{13}C NMR (150 MHz, $CDCl_3$): δ 144.0 (d, $^2J_{CP}$ = 15.0 Hz, C_{pyr}); 136.3 (d, $^3J_{CP}$ = 5.6 Hz, C_{pyr}); 135.5 (d, $^3J_{CP}$ = 6.0 Hz, C_{pyr}); 131.6 (C_{pyr}); 131.2 (d, $^4J_{CP}$ = 3.1 Hz, C_{pyr}); 130.9 (C_{pyr}); 129.9 (C_{pyr}); 129.6 (C_{Ar}); 129.4 (d, $^3J_{CP}$ = 6.5 Hz, C_{pyr}); 129.2 (C_{Ar}); 128.8 (C_{Ar}); 128.7 (C_{Ar}); 128.3 (C_{pyr}); 128.2 (C_{Ar}); 128.1 (C_{Ar}); 127.9 (C_{Ar}); 127.8 (C_{pyr}); 127.7 (C_{pyr}); 126.2 (C_{pyr}); 125.6 (C_{pyr}); 125.5 (d, $^4J_{CP}$ = 3.2 Hz, C_{pyr}); 125.4 (C_{pyr}); 125.3 (C_{pyr}); 125.2 (d, $^4J_{CP}$ = 1.7 Hz, C_{Ar}); 125.0 (C_{Ar}); 122.5 (C_{Ph}); 114.2 (C_{Ph}); 68.9 (d, $^2J_{CP}$ = 13.1 Hz, PO*C*); 68.8 (d, $^2J_{CP}$ = 13.0 Hz, PO*C*); 52.9 (d, $^1J_{CP}$ = 151.1 Hz, P*C*); 20.5 (C_{Ar}-C). ^{31}P NMR (243 MHz, $CDCl_3$): δ 23.63. IR (KBr): 3447 (νNH); 3319 (νCH); 2957 (CH_3); 1614, 1523, 1456 (C=C); 1229 (P=O); 1020 (P-O); 848; 822; 620. Anal. Calcd for $C_{38}H_{32}NO_3P$ $^1/_{20}$ $CHCl_3$: C, 77.77; H, 5.50; N, 2.38. Found: C, 77.85; H, 5.75; N, 2.09.

*1.2.4.4. Dibenzyl N-(4-methoxyphenyl)amino(pyren-1-yl)methylphosphonate (**3Cd**)*

Y = (341 mg, 57%), white solid, m.p. 156-157°C. ^{1}H NMR ($CDCl_3$, 600 MHz): δ 8.37 (d, $^3J_{HH}$ = 9.4 Hz, H_{pyr}, 1H); 8.28 (dd, $^3J_{HH}$ = 8.0 and $^4J_{HH}$ = 2.6 Hz, H_{pyr}, 1H); 8.21 (d, H_{pyr}, $^3J_{HH}$ = 7.7 Hz, 1H); 8.19 (d, H_{pyr}, $^3J_{HH}$ = 7.6 Hz, 1H); 8.13 (d, $^3J_{HH}$ = 7.9 Hz, H_{pyr}, 1H); 8.09 (d, $^3J_{HH}$ = 9.2 Hz, H_{pyr}, 1H); 8.07 (d, $^3J_{HH}$ = 8.5 Hz, H_{pyr}, 1H); 8.04-8.01 (m, H_{pyr}, 2H); 7.37-7.36 (m, H_{Ph}, 1H); 7.30-7.29 (m, H_{Ph}, 4H); 6.94 (dt, $^3J_{HH}$ = 7.5 and $^4J_{HH}$ = 1.3 Hz, H_{Ph}, 1H); 6.84 (ddd, $^3J_{HH}{}^{(1)}$ = $^3J_{HH}{}^{(2)}$ = 7.8 and $^4J_{HH}$ = 1.7 Hz, H_{Ph}, 1H); 6.68 (dd, $^3J_{HH}$ = 7.1 and $^4J_{HH}$ = 1.1 Hz, H_{Ph}, 1H); 6.79 (app. d, $^3J_{HH}$ = 9.1 Hz, *p*-C_6H_4, 2H); 6.46 (app. d, $^3J_{HH}$ = 9.1 Hz, *p*-C_6H_4, 2H); 5.88 (d, $^2J_{PH}$ = 24.0 Hz, CHP, 1H); 5.10-5.08 (Part of ABX system, $^2J_{HH}$ = 11.7 and $^3J_{PH}$

= 9.1 and 5.9 Hz, $POCH_2$, 2H); 4.66 and 4.25 (Part of AMX system, $^2J_{HH}$ = 11.7 Hz and $^3J_{PH}$ = 7.8 and 8.4 Hz, $POCH_2$, 2H); 3.59 (s, CH_3, 3H). ^{13}C NMR (150 MHz, $CDCl_3$): δ 152.9 (C_{pyr}); 140.4 (d, $^2J_{CP}$ = 15.5 Hz, C_{pyr}); 136.3 (d, $^3J_{CP}$ = 5.5 Hz, C_{pyr}); 135.5 (d, $^4J_{CP}$ = 2.6 Hz, C_{pyr}); 131.6 (C_{pyr}); 131.2 (d, $^4J_{CP}$ = 3.2 Hz, C_{pyr}); 130.8 (C_{pyr}); 129.7 (d, $^4J_{CP}$ = 3.3 Hz, C_{pyr}); 129.5 (C_{pyr}); 129.4 (d, $^3J_{CP}$ = 6.5 Hz, C_{pyr}); 128.7 (C_{pyr}); 128.6 (C_{pyr}); 128.3 (C_{pyr}); 128.2 (C_{pyr}); 128.1 (C_{pyr}); 127.8 (C_{pyr}); 127.6 (d, $^3J_{CP}$ = 6.6 Hz, C_{pyr}); 127.1 (C_{ar}); 126.2 (C_{pyr}); 125.6 (C_{pyr}); 125.5 (d, $^4J_{CP}$ = 3.2 Hz, C_{pyr}); 125.4 (C_{pyr}); 125.3 (C_{pyr}); 125.2 (C_{pyr}); 125.0 (C_{pyr}); 124.0 (C_{Ar}); 122.2 (C_{Ph}); 115.4 (C_{Ph}); 115.0 (C_{Ph}); 68.9 (d, $^2J_{CP}$ = 17.5 Hz, PO*C*); 68.8 (d, $^2J_{CP}$ = 17.5 Hz, PO*C*); 55.8 (C_{Ar}-O*C*); 53.4 (d, $^1J_{CP}$ = 151.2 Hz, P*C*). ^{31}P NMR (243 MHz, $CDCl_3$): δ 23.62. IR (KBr): 3432 (νNH); 3294 (νCH); 3026; 2951 (CH_3); 1588, 1510, 1456 (C=C); 1235 (P=O);1049; 1031 (P-O); 844; 820; 615. Anal. Calcd for $C_{38}H_{32}NO_4P$: C, 76.37; H, 5.40; N, 2.34. Found: C, 76.28, H, 5.53, N, 2.46.

*1.2.4.5. Dibenzyl N-furfurylamino(pyren-1-yl)methylphosphonate (**3Cg**)*

Y = (114 mg, 20%) white solid, m.p. 65°C. ^{1}H NMR ($CDCl_3$, 600 MHz): δ 8.45-8.41 (m, H_{pyr}, 1H); 8.21 (d, $^3J_{HH}$ = 7.9 Hz, H_{pyr}, 3H); 8.17 (d, $^3J_{HH}$ = 7.5 Hz, H_{pyr}, 1H); 8.10 (d, $^3J_{HH}$ = 8.8 Hz, H_{pyr}, 1H); 8.07 (d, $^3J_{HH}$ = 8.9 Hz, H_{pyr}, 2H); 8.02 (t, $^3J_{HH}$ = 7.5 Hz, H_{pyr}, 1H); 8.01 (d, $^3J_{HH}$ = 9.2 Hz, H_{pyr}, 1H); 7.37-7.36 (m, PhH, 1H); 7.32-7.30 (m, PhH, H_{fur}, 2H); 7.24-7.21 (m, PhH, 3H); 7.03-7.01 (m, PhH, 1H); 6.97-6.95 (m, PhH, 2H); 6.87-6.85 (m, PhH, 2H); 6.24 (dd, $^3J_{HH}$ = 1.9 and 3.1 Hz, H_{fur}, 1H); 5.97-5.96 (m, H_{fur}, 1H); 5.31 (d, $^2J_{PH}$ = 19.6 Hz, CHP, 1H); 5.06 and 4.94 (the AM part of AMX system, $^2J_{HH}$ = 11.8 Hz and $^3J_{PH}$ = 8.9 and 7.1 Hz, OCH_2Ph, 2H); 4.77 and 4.57 (the AM part of AMX system, $^2J_{HH}$ = 11.8 Hz and $^3J_{PH}$ = 8.7 and 7.5 Hz, OCH_2Ph, 2H); 3.87 (d, $^2J_{HH}$ = 14.5 Hz, CH_2Fur, 1H); 3.59 (d, $^2J_{HH}$ = 14.57 Hz, CH_2Fur, 1H). ^{13}C NMR (150 MHz, $CDCl_3$): δ 142.3 (C_{fur}); 136.5 (d, $^3J_{CP}$ = 6.1 Hz, C_{Ar}); 136.0 (d, $^3J_{CP}$ = 6.1 Hz, C_{Ar}); 131.6 (C_{pyr}); 131.2 (d, $^4J_{CP}$ = 3.1 Hz, C_{pyr}); 130.9 (C_{pyr}); 130.2 (d, $^3J_{CP}$ = 7.5 Hz, C_{pyr}); 128.7 (C_{Ar}); 128.6 (C_{Ar}); 128.4 (C_{Ar}); 128.2 (d, $^3J_{CP}$ = 8.7 Hz, C_{Ar}); 128.1 (C_{Ar}); 128.0 (C_{Ar}); 127.9 (C_{pyr}); 127.8 (C_{pyr}); 127.6 (d, $^3J_{CP}$ = 1.5 Hz, C_{pyr}); 127.2 (C_{pyr}); 126.2 (C_{pyr}); 125.5 (C_{pyr}); 125.3 (d, $^4J_{CP}$ = 3.0 Hz, C_{pyr}); 125.2 (C_{pyr}); 125.1 (C_{pyr}); 125.0 (C_{pyr}); 123.0 (C_{pyr}); 110.3 (C_{fur}); 108.2 (C_{fur}); 68.7 (d, $^2J_{CP}$ = 6.9 Hz, PO*C*); 68.5 (d, $^2J_{CP}$ = 6.9 Hz, PO*C*); 44.0 (d, $^3J_{CP}$ = 17.5 Hz, PCN*C*). ^{31}P NMR (243 MHz, $CDCl_3$): δ 23.81. IR (KBr): 3433(νNH); 3291 (νCH); 2958 (CH_2); 1609, 1584, 1515, 1458 (C=C); 1242 (P=O);1055; 1024 (P-O); 992; 821; 739; 622. Anal. Calctd for $C_{36}H_{30}NO_4P$: C, 75.64; H, 5.29; N, 2.45. Found: C, 75.37, H, 5.39, N, 2.39.

1.3. Preparation of aminophosphonates 3Ai,j via the Kabachnik–Fields reaction

Equimolar quantities (1mmol) of pyrene-1-carboxaldehyde, *tert*-butylamine or 3-methylphenylamine and dimethyl phosphite were dissolved in a small amount of acetonitrile or methanol (for 3-methylphenylamine), then a catalytic amount of trifluoroacetic acid was added. The mixture was stirred and heated for 24 h and then, the solvent was removed under reduced pressure.

The crude reaction mixture was dissolved in dichloromethane and washed three times with a saturated aqueous solution of sodium bicarbonate. The organic layer was dried and the solvent

was evaporated in vacuo yielding crude aminophosphonate as a yellow solid or yellow oil which was further purified by column chromatography on silica gel with chloroform as eluent.

*1.3.1. Dimethyl N-(3-methylphenyl)amino(pyren-1-yl)methylphosphonate (**3Ai**)*

Y = (120 mg, 28%), white solid, m.p: 209-210°C. ^{1}H NMR ($CDCl_3$, 600 MHz): δ 8.49 (d, $^{3}J_{HH}$ = 9.3 Hz, H_{pyr}, 1H); 8.31 (dd, $^{3}J_{HH}$ = 8.0 and $^{4}J_{HH}$ = 2.6 Hz, H_{pyr}, 1H); 8.25-8.21 (m, H_{pyr}, 3H); 8.17 (d, $^{3}J_{HH}$ = 7.6 Hz, H_{pyr}, 1H); 8.08 (d, $^{3}J_{HH}$ = 8.8 Hz, H_{pyr}, 1H); 8.04-8.03 (m, H_{pyr}, 2H); 6.87 (app. t, $^{3}J_{HH}$ = 7.8 Hz, *m*-C_6H_4, 1H); 6.50 (m, *m*-C_6H_4, 1H); 6.46 (app. d, $^{3}J_{HH}$ = 7.6 Hz, *m*-C_6H_4, 1H); 6.38-6.37 (m, *m*-C_6H_4, 1H); 5.95 (d, $^{2}J_{PH}$ = 24.0 Hz, CHP, 1H); 3.84 (d, $^{3}J_{PH}$ = 10.7 Hz, $POCH_3$, 3H); 3.12 (d, $^{3}J_{PH}$ = 10.5 Hz, $POCH_3$, 3H); 2.12 (s, CH_3, 3H). ^{13}C NMR (150 MHz, $CDCl_3$): δ 146.4 (C_{ar}); 146.3 (C_{ar}); 139.2 (C_{ar}); 131.6 (C_{pyr}); 131.3 (d, $^{4}J_{CP}$ = 2.9 Hz, C_{pyr}); 130.9 (C_{pyr}); 129.6 (d, $^{4}J_{CP}$ = 3.1 Hz, C_{pyr}); 129.3 (C_{pyr}); 129.2 (d, $^{3}J_{CP}$ = 6.5 Hz, C_{pyr}); 128.4 (C_{pyr}); 127.8 (C_{pyr}); 127.7 (d, $^{4}J_{CP}$ = 1.6 Hz, C_{pyr}); 126.3 (C_{pyr}); 125.7 (C_{pyr}); 125.6 (d, $^{4}J_{CP}$ = 3.4 Hz, C_{pyr}); 125.5 (C_{pyr}); 125.4 (d, $^{3}J_{CP}$ = 4.7 Hz, C_{pyr}); 125.2 (d, $^{4}J_{CP}$ = 1.9 Hz, C_{pyr}); 125.0 (C_{pyr}); 122.2 (d, $^{4}J_{CP}$ = 1.6 Hz, C_{Ar}); 119.8 (C_{Ar}); 115.0 (C_{Ph}); 110.8 (C_{Ph}); 54.0 (d, $^{2}J_{CP}$ = 7.0 Hz, PO*C*); 53.9 (d, $^{2}J_{CP}$ = 7.3 Hz, PO*C*); 52.1 (d, $^{1}J_{CP}$ = 151.7 Hz, P*C*); 21.7 (C_{Ar}-C). ^{31}P NMR (243 MHz, $CDCl_3$): δ 25.08. IR (KBr): 3416 (νNH); 3307 (νCH); 2948 (CH_3); 1608, 1491 (C=C); 1239 (P=O);1059; 1027 (P-O); 841; 775; 617. Anal. Calcd for $C_{26}H_{24}NO_3P$x$^{1}/_{7}$ $CHCl_3$:: C, 70.32; H, 5.45; N, 3.14. Found: C, 70.37, H, 5.47, N, 3.10.

*1.3.2. Dimethyl N-(tert-butyl)amino(pyren-1-yl)methylphosphonate (**3Aj**)*

Y = (374 mg, 95%), white solid, m.p. 186°C. ^{1}H NMR ($CDCl_3$, 600 MHz): δ 8.42-8.40 (m, H_{pyr}, 1H); 8.38 (d, $^{3}J_{HH}$ = 9.3 Hz, H_{pyr}, 1H); 8.23 (d, $^{3}J_{HH}$ = 8.0 Hz, H_{pyr}, 1H); 8.20 (d, $^{3}J_{HH}$ = 9.1 Hz, H_{pyr}, 2H); 8.17 (d, $^{3}J_{HH}$ = 9.2 Hz, H_{pyr}, 1H); 8.09-8.05 (m, H_{pyr}, 2H); 8.01 (t, $^{3}J_{HH}$ = 7.6 Hz, H_{pyr}, 1H); 5.40 (dd, $^{2}J_{PH}$ = 25.2 and $^{3}J_{HH}$ = 8.0 Hz, CHP, 1H); 3.87 (d, $^{3}J_{PH}$ = 10.4 Hz, $POCH_3$, 3H); 3.82 (br s, NH, 1H); 3.20 (d, $^{3}J_{PH}$ = 10.3 Hz, $POCH_3$, 3H); 0.99 (s, CH_3, 9H). ^{13}C NMR (150 MHz, $CDCl_3$): δ 131.7 (C_{pyr}); 130.9 (C_{pyr}); 128.5 (C_{pyr}); 128.4 (C_{pyr}); 128.3 (C_{pyr}); 127.8 (C_{pyr}); 127.6 (C_{pyr}); 126.5 (C_{pyr}); 126.2 (C_{pyr}); 125.6 (C_{pyr}); 125.2 (C_{pyr}); 125.1 (C_{pyr}); 125.1 (C_{pyr}); 122.3 (C_{pyr}); 54.7 (d, $^{2}J_{CP}$ = 7.2 Hz, PO*C*); 53.4 (PCN*C*); 50.4 (d, $^{1}J_{CP}$ = 158.2 Hz, P*C*); 30.1 (C_{aliph}). ^{31}P NMR (243 MHz, $CDCl_3$): δ 26.34. IR (KBr): 3424 (νNH); 3279 (νCH); 2969 (CH_3); 1603, 1444 (C=C); 1244 (P=O);1062; 1035 (P-O); 847; 788; 618. Anal. Calcd for $C_{23}H_{26}NO_3P$x$^{1}/_{9}$ $CHCl_3$: C, 67.92; H, 6.44; N, 3.43. Found: C, 67.87, H, 6.32, N, 3.32.

1.4. Preparation of aminophosphonic acids 4a and 4c

The compounds **2a** or **2c** (1 mmol) were dissolved in a small amount of a dry methylene chloride and (3 mmol) of trimethylsilyl bromide was added. The mixture was stirred for 24 h at room temperature. Then the solvent was evaporated, a small amount of dry methanol was added and stirring was continued for 24 h at room temperature. The resulting precipitate was filtered and washed with a small amount of water.

*1.4.1. N-benzylamino(pyren-1-yl)methylphosphonic acid (**4a**)*

Y = (172 mg, 43%), white solid, m.p. 235-236°C. ^{1}H NMR ($CDCl_3$, 600 MHz): δ 8.50-8.46 (m, H_{pyr}, 1H); 8.41 (d, $^3J_{HH}$ = 8.0 Hz, H_{pyr}, 1H); 8.36 (d, $^3J_{HH}$ = 7.6 Hz, H_{pyr}, 1H); 8.34 (d, $^3J_{HH}$ = 7.5 Hz, H_{pyr}, 1H); 8.27 (d, $^3J_{HH}$ = 8.9 Hz, H_{pyr}, 1H); 8.23 (d, $^3J_{HH}$ = 9.0 Hz, H_{pyr}, 1H); 8.21 (d, $^3J_{HH}$ = 9.4 Hz, H_{pyr}, 1H); 8.13 (t, $^3J_{HH}$ = 7.6 Hz, H_{pyr}, 1H); 8.03-7.97 (m, H_{pyr}, 1H); 7.40-7.37 (m, PhH, 2H); 7.33-7.32 (m, PhH, 3H); 5.45 (d, $^2J_{PH}$ = 17.8 Hz, CHP, 1H); 4.37 (d, $^2J_{HH}$ = 13.3 Hz, CH_2Ph, 1H); 3.57 (d, $^2J_{HH}$ = 13.3 Hz, CH_2Ph, 1H). ^{13}C NMR (150 MHz, $CDCl_3$): δ 130.8 (C_{pyr}); 130.1 (C_{pyr}); 129.9 (C_{pyr}); 128.8 (C_{pyr}); 127.3 (C_{pyr}); 127.2 (C_{pyr}); 127.0 (C_{pyr}); 126.8 (C_{pyr}); 126.1 (C_{pyr}); 125.2 (C_{pyr}); 124.9 (C_{ar}); 124.7 (C_{ar}); 123.9 (C_{arr}); 123.8 (C_{ar}); 54.6 (d, $^1J_{CP}$ = 135.0 Hz, PC); 49.2 (PCN*C*). ^{31}P NMR (243 MHz, $CDCl_3$): δ 11.42. IR (KBr): 3377 (νNH); 3040; 2980 (CH_2); 1618, 1458, 1453 (C=C); 1231 (P=O); 1103 (P-O); 849; 742; 615. Anal. Calcd for $C_{24}H_{20}NO_3P$ $^4/_3$ H_2O: C, 67.76; H, 5.37; N, 3.29. Found: C, 67.87; H, 5.52; N, 3.13.

*1.4.2. N-(p-methylphenyl)amino(pyren-1-yl)methylphosphonic Acid (**4c**)*

Y = (96 mg, 24%), red solid, m.p. 210-211°C. ^{1}H NMR ($CDCl_3$, 600 MHz): δ 8.64 (d, $^3J_{HH}$ = 9.4 Hz, H_{pyr}, 1H); 8.49 (d, $^3J_{HH}$ = 7.9 Hz, H_{pyr}, 1H); 8.29-8.25 (m, H_{pyr}, 3H); 8.19 (d, $^3J_{HH}$ = 8.3 Hz, H_{pyr}, 1H); 8.14 (d, $^3J_{HH}$ = 8.8 Hz, H_{pyr}, 1H); 8.10 (d, $^3J_{HH}$ = 8.8 Hz, H_{pyr}, 1H); 8.07 (t, $^3J_{HH}$ = 7.6 Hz, H_{pyr}, 1H); 6.68 (app. d, $^3J_{HH}$ = 8.2 Hz, PhH, 2H); 6.52 (app. d, $^3J_{HH}$ = 8.3 Hz, PhH, 2H); 5.70 (d, $^2J_{PH}$ = 23.7 Hz, CHP, 1H); 1.98 (s, CH_3, 3H). ^{13}C NMR (150 MHz, $CDCl_3$): δ 145.2 (d, $^2J_{CP}$ = 14.6 Hz, C_{Ar}); 134.9 (C_{Ar}); 133.4 (C_{Ar}); 130.9 (C_{Ar}); 130.8 (C_{Ar}); 130.6 (d, $^3J_{CP}$ = 7.0 Hz, C_{Ar}); 130.5 (C_{Ar}); 130.2 (C_{Ar}); 129.7 (d, $^3J_{CP}$ = 7.5 Hz, C_{Ar}); 129.0 (C_{Ar}); 128.9 (d, $^3J_{CP}$ = 5.6 Hz, C_{Ar}); 127.3 (C_{Ar}); 127.2 (d, $^4J_{CP}$ = 3.1 Hz, C_{Ar}); 127.0 (C_{Ar}); 126.9 (d, $^3J_{CP}$ = 7.7 Hz, C_{Ar}); 126.7 (C_{Ar}); 126.0 (C_{Ar}); 125.6 (C_{Ar}); 124.9 (d, $^4J_{CP}$ = 5.1 Hz, C_{Ar}); 124.8 (C_{Ar}); 124.7 (C_{Ar}); 124.6 (C_{Ar}); 123.9 (C_{Ar}); 123.8 (C_{Ar}); 123.7 (C_{Ar}); 123.5 (C_{Ar}); 122.5 (C_{Ar}); 121.3 (C_{Ar}); 113.2 (C_{Ar}); 52.5 (d, $^1J_{CP}$ = 145.2 Hz, P*C*); 19.8 (Ar-*C*). ^{31}P NMR (243 MHz, $CDCl_3$): δ 17.52. IR (KBr): 3393 (νNH); 3040; 2919 (CH_2); 1616, 1515; 1457 (C=C); 1183 (P=O); 1062 (P-O); 848; 612. Anal. Calcd for $C_{24}H_{20}NO_3P$: C, 71.81; H, 5.02; N, 3.49. Found: C, 71.56; H, 5.09; N, 3.21.

1.5. Preparation of dimethyl hydroxy(pyren-1-yl)methylphosphonate (5A) via modified Pudovik reaction

Equimolar quantities (1 mmol) of pyrene-1-carboxaldehyde, *tert*-butylamine and dimethyl phosphite were dissolved in a small amount of dichloromethane and stirred at reflux for 24 h. After this time the solvent was removed under reduced pressure. The crude reaction mixture was dissolved in dichloromethane and washed once with a saturated aqueous sodium bicarbonate solution. The organic layer was dried and solvent was evaporated in vacuo yielding crude hydroxymethylphosphonate as a yellow solid which was further purified by column chromatography on silica gel with chloroform as eluent to give 163 mg (48%) of **5A** as a yellow solid, m.p. 193°C. ^{1}H NMR ($CDCl_3$, 600 MHz): δ 8.40 (dd, $^3J_{HH}$ = 8.0 Hz, $^4J_{HH}$ = 2.2 Hz, H_{pyr}, 1H); 8.32 (d, $^3J_{HH}$ = 9.3 Hz, H_{pyr}, 1H); 8.25 (d, $^3J_{HH}$ = 8.0 Hz, H_{pyr}, 1H); 8.22-8.21 (m, H_{pyr}, 2H); 8.15 (d, $^3J_{HH}$ = 9.3 Hz, H_{pyr}, 1H); 8.10 (d, $^3J_{HH}$ = 8.9 Hz, H_{pyr}, 1H); 8.07 (d,

$^3J_{HH}$ = 8.9 Hz, H_{pyr}, 1H); 8.03 (t, $^3J_{HH}$ = 7.6 Hz, H_{pyr}, 2H); 6.19 (d, $^2J_{PH}$ = 11.3 Hz, CHP, 1H); 3.71 (d, $^3J_{PH}$ = 10.4 Hz, $POCH_3$, 3H); 3.52 (d, $^3J_{PH}$ = 10.4 Hz, $POCH_3$, 3H). ^{13}C NMR (150 MHz, $CDCl_3$): δ 131.5 (C_{pyr}); 130.8 (C_{pyr}); 129.9 (C_{pyr}); 128.6 (C_{pyr}); 128.5 (C_{pyr}); 128.1 (C_{pyr}); 128.0 (C_{pyr}); 127.6 (d, $^4J_{CP}$ = 1.4 Hz, C_{pyr}); 126.3 (C_{pyr}); 125.7 (C_{pyr}); 125.5 (C_{pyr}); 125.3 (d, $^4J_{CP}$ = 5.1 Hz, C_{pyr}); 125.2 (d, $^4J_{CP}$ = 3.0 Hz, C_{pyr}); 124.9 (C_{pyr}); 124.9 (C_{pyr}); 122.8 (C_{pyr}); 66.4 (d, $^1J_{CP}$ = 160.1 Hz, P*C*); 54.1 (d, $^2J_{CP}$ = 6.8 Hz, PO*C*); 53.9 (d, $^2J_{CP}$ = 7.1 Hz, PO*C*). ^{31}P NMR (243 MHz, $CDCl_3$): δ 23.65. IR (KBr): 3236 (νOH); 2950 (CH); 1585, 1454 (C=C); 1213 (P=O);1059 (C-O); 1039 (P-O); 848; 831; 764; 617. Anal. Calctd for $C_{19}H_{17}O_4P$ $^1/_{20}$ CH_2Cl_2: C, 65.46; H, 4.95. Found: C, 65.47; H, 4.93.

2. Biological studies

2.1. Materials and methods

MTT assay (3-(4,5-dimethylthiazol-2-yl)-2,5-diphenyltetrazolium bromide) facilitates the determination of the level of cell survival after the exposure of cells to potentially toxic substances. The cytotoxicity level of studied compounds was evaluated by determining the dose or concentration that induces a standard biological response. The assessment of the cytotoxic activity of the tested compounds was carried out based on IC_{50} values – the concentration of the compound which causes inhibition of cell proliferation by 50% as compared to the negative control. To determine the above concentration, was created graphs of cell viability expressed in percentages, depending on the concentrations of studied compounds.

2.2. Chemicals

MTT (3(4,5-dimethylthiazol-2-yl)-2,5-diphenyltetrazolium bromide), lauryl sulfate (SLS), *N,N*-dimethylformamide (DMF), dimethyl sulfoxide (DMSO), penicillin-streptomycin solution stabilized, MEM non-essential amino acids solution, Histopaque 1077, and buffered saline (PBS) were purchased from Sigma Chemical Co. Foetal bovine serum (FBS), phytohemagglutinin (PHA), RPMI 1640 medium and Trypsin-EDTA were supplied by CytoGen (Poland).

All other chemicals (chemical reagents, substrates, and solvents) were of the highest commercial grade available and were purchased from CytoGen and Sigma–Aldrich.

2.3. Cell culture

Lymphocytes were isolated from the peripheral blood of healthy, non-smoking donors of both sexes by centrifugation in a density gradient of Histopaque 1077 (15 min., 300 × g) (blood was obtained from the Blood Donation Center in Lodz, Poland). The cells were suspended in the culture medium containing RPMI 1640, inactivated FBS (15%), penicillin and streptomycin (1%) and PHA (1%).

The human colorectal carcinoma cell line (HCT116) and the human colorectal adenocarcinoma cell line (HT29) were obtained from the American Type Culture Collection (ATCC, Rockville, USA). The HCT116 cells were cultured in RPMI 1640 supplemented with FBS (10%) and antibiotics (1%): penicillin and streptomycin. The HT29 cells were cultured in RPMI 1640 medium supplemented with FBS (10%), antibiotics (1%): penicillin and streptomycin, and MEM non-essential amino acids solution (1%). The lymphocytes, HCT116 and HT29 cells were cultured at 37 °C in a humidified atmosphere of 95% air and 5% CO_2.

2.4. MTT assay

A MTT assay was used to assess cell survival after treatment with studied compounds. The MTT assay is a quantitative colorimetric method to determine cell proliferation after treatment with the tested compounds. It is widely used to estimate the cytotoxic effect of chemicals on different types of cells [2]. MTT (3(4,5-dimethyl-2-thiazolyl)-2,5-diphenyl-2*H*-tetrazolium bromide) is a water-soluble tetrazolium salt, which is converted into an insoluble purple formazan by cleavage of the tertazolium ring by succinate dehydrogenase within the mitochondria. The formazan product is impermeable to the cell membranes and therefore it accumulates in living cells. The purple crystals were extracted into a solution for homogeneous staining and the absorbance was read on a spectrophotometer at 595 nm. The percent reduction of MTT was compared to controls (cells not exposed to tested compounds), which represented 100% MTT reduction [3]. The assay was optimized for the cell lines and chemical compounds used in the experiments.

The cancer cells and human lymphocytes were grown for 24 h on 96-well plates at a density of 6–8 × 10^3 cells/well and 8 × 10^5 cells/well, respectively. Then the cells were treated with the tested compounds for 24 h incubation periods in a volume of 50 μl per well for the lymphocytes and 100 μl per well for the HCT116, HT29 cells. Subsequently, a fresh MTT solution (lymphocytes, HCT116 and HT29: 20 μl, 5 mg/ml in sterile PBS) was added to each well for the next 4 h. Purple crystals forming in the cancer cells after the reduction of MTT were dissolved by DMSO (100 μl/well). In the case of lymphocytes this was achieved by adding a 100 μl mixture of 20% SDS and 50% DMF to each well for 24 h. The absorbance was measured with a PowerWave XS spectrophotometer (BioTek Instruments, Inc.). The antiproliferative effect was expressed as the % of cells surviving. All of the results were presented as the mean ± SEM of the replicates from six independent experiments.

3. Fluorescence studies

3.1. Materials and methods

Electronic absorption spectra were run on a PerkinElmer Lambda 45 UV–vis Spectrometer and emission spectra on a PerkinElmer LS55 Fluorescence Spectrometer. Emission quantum yields were measured using quinine sulfate in 0.5 M sulfuric acid as a reference (Φ_F = 0.55) [4].

4. References to the experimental part

1. Jayaprakash S.H.; Uma Maheswara Rao K.; Satheesh Krishna B.; Siva Prasad S.; Syama Sundar C.; Suresh Reddy C. *Phosphorus Sulfur Silicon Relat. Elem.* **2015**, *190*, 449-460. doi: 10.1080/10426507.2014.948621
2. Abe, K.; Matsuki, N. *Neurosci. Res.* **2000**, *38*, 325-329. doi:10.1016/S0168-0102(00)00188-7
3. Fotakis, G., Timbrell, J.A. *Toxicology Lett.* **2006**, *160*, 171–177. doi:10.1016/j.toxlet.2005.07.001
4. Brouwer A.M. *Pure Appl Chem.* **2011**, *83(12)*, 2213-2228. doi:10.1351/PAC-REP-10-09-31

^{1}H NMR spectra of studied compounds 3, 4 and 5A

Dimethyl *N*-benzylamino(pyren-1-yl)methylphosphonate (**3Aa**)

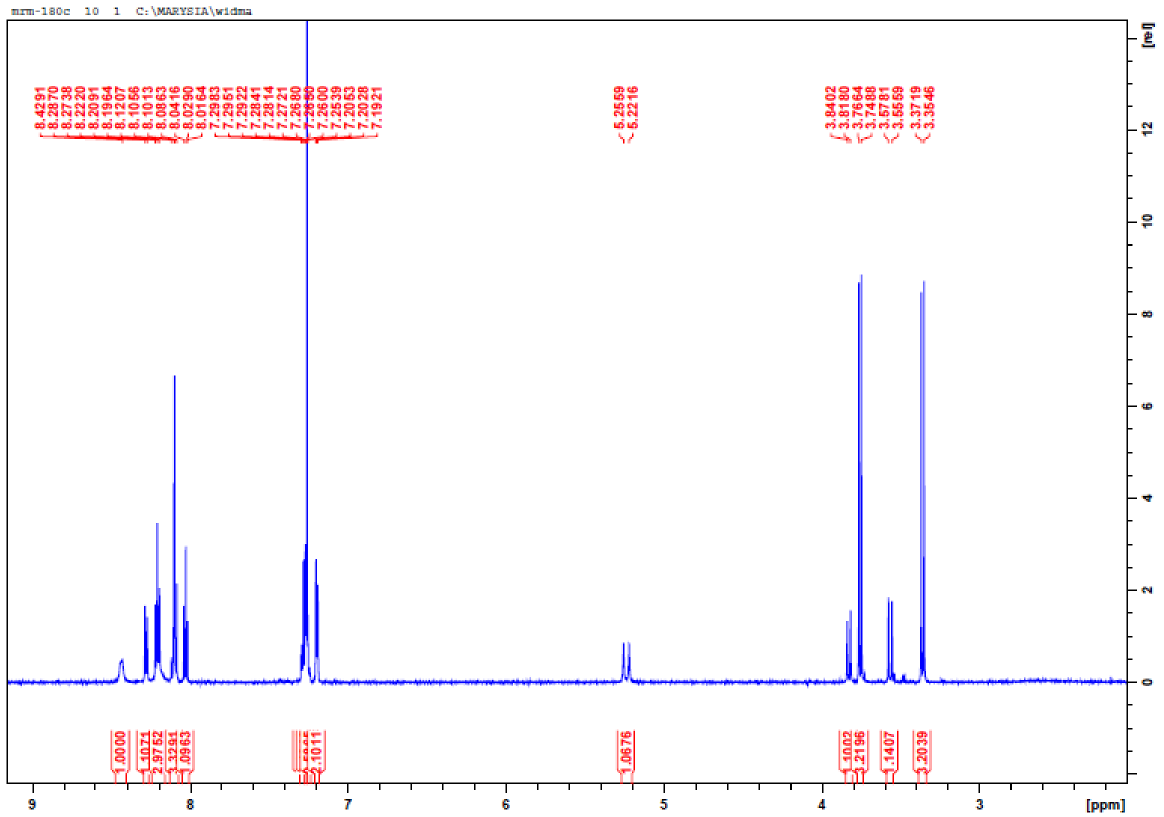

Dimethyl *N*-phenylamino(pyren-1-yl)methylphosphonate (**3Ab**)

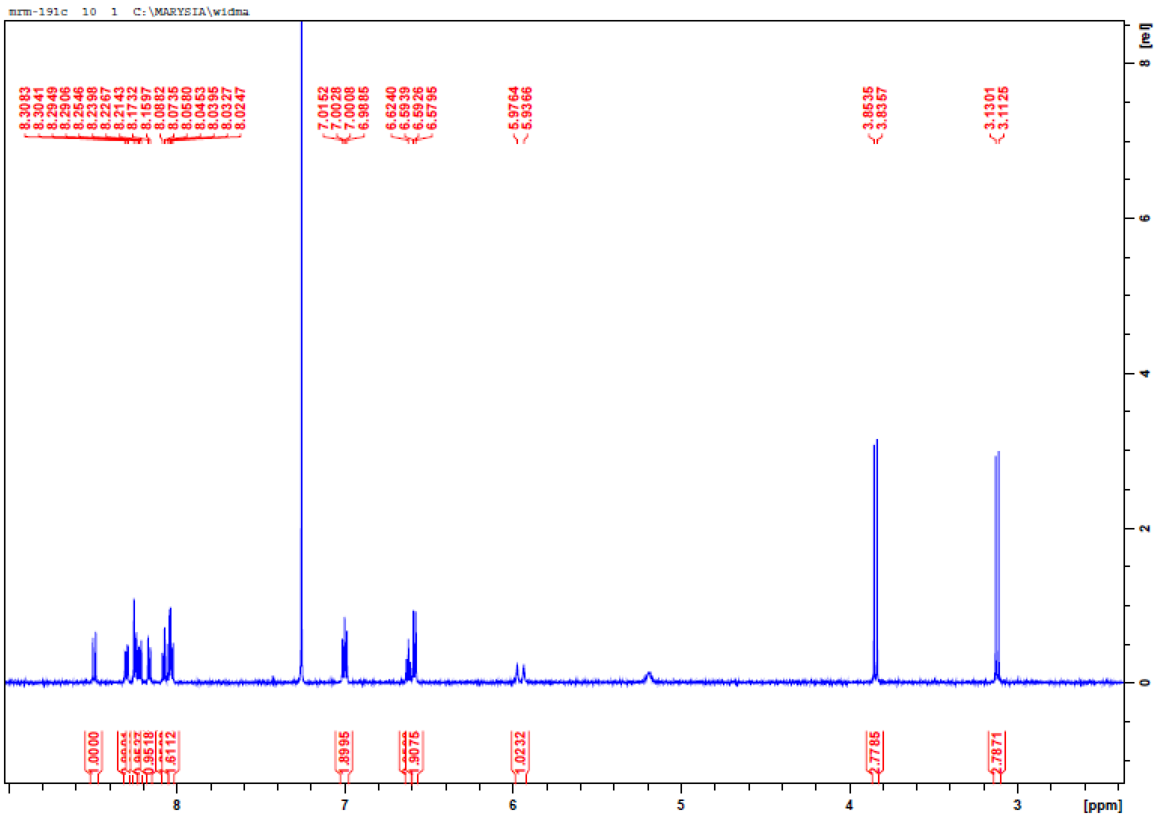

Dimethyl *N*-(4-methylphenyl)amino(pyren-1-yl)methylphosphonate (**3Ac**)

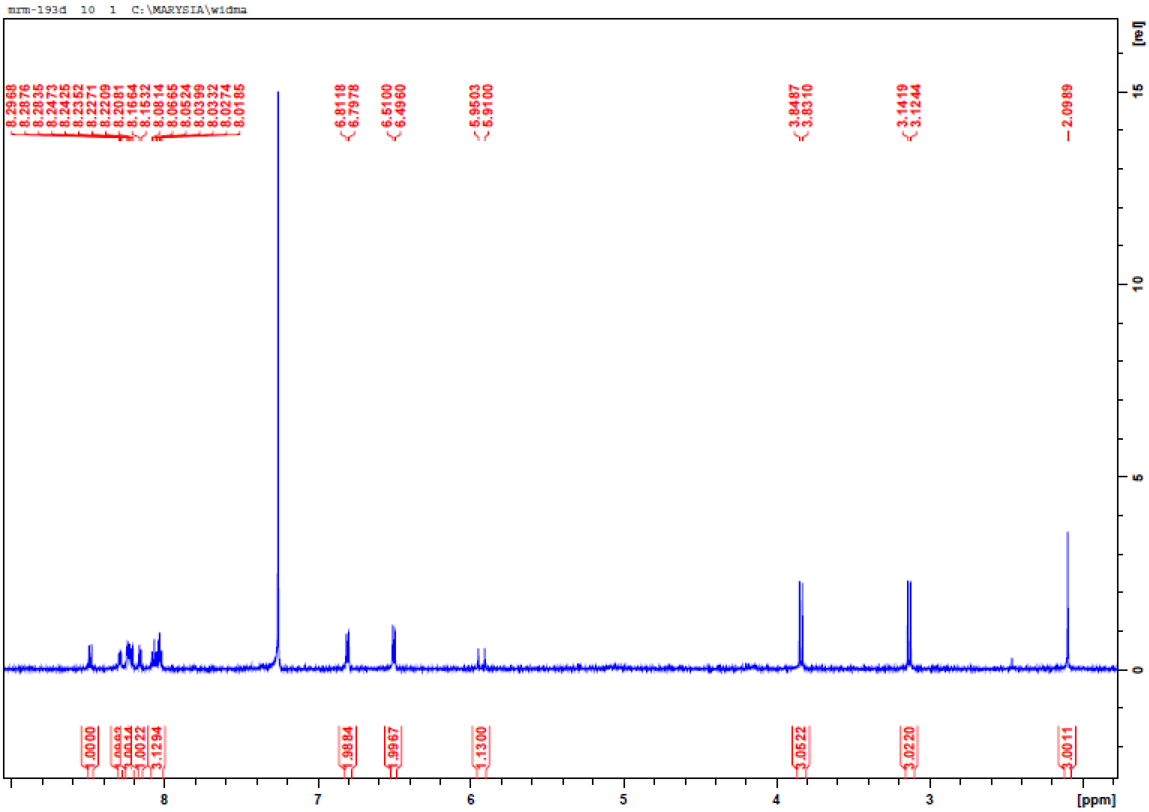

Dimethyl *N*-(4-methoxyphenyl)amino(pyren-1-yl)methylphosphonate (**3Ad**)

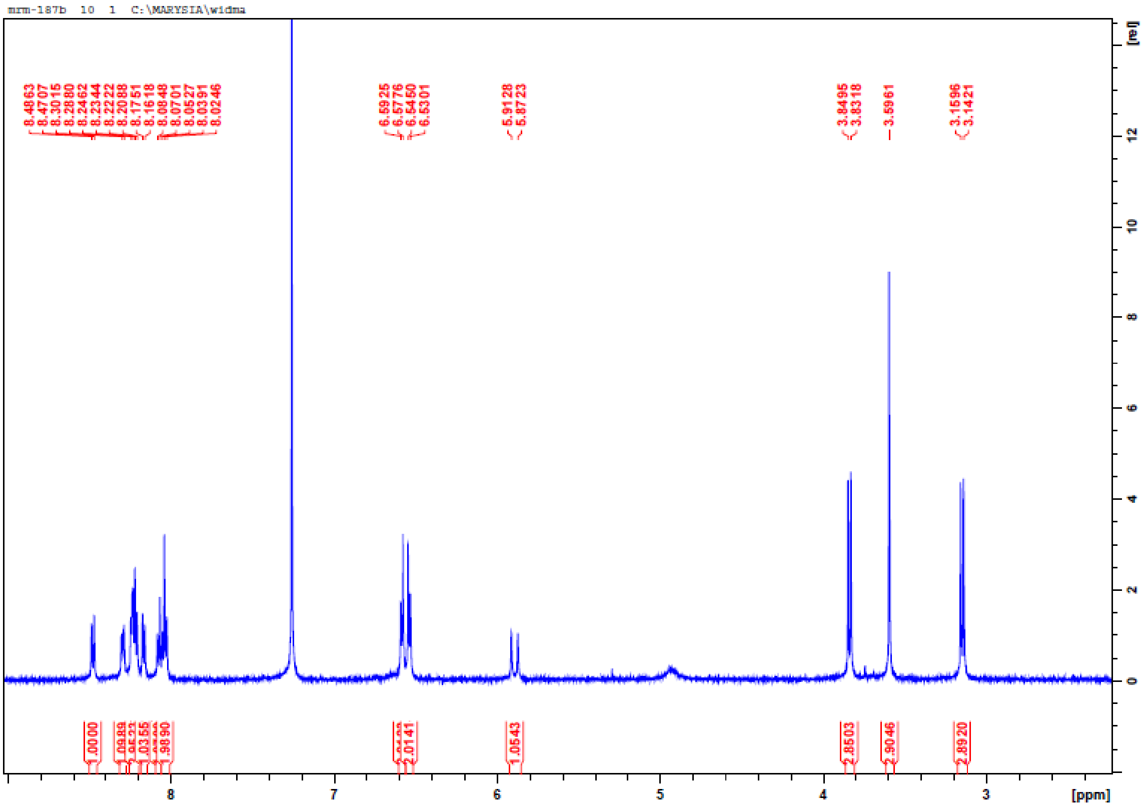

Dimethyl *N*-(1-butyl)amino(pyren-1-yl)methylphosphonate (**3Ae**)

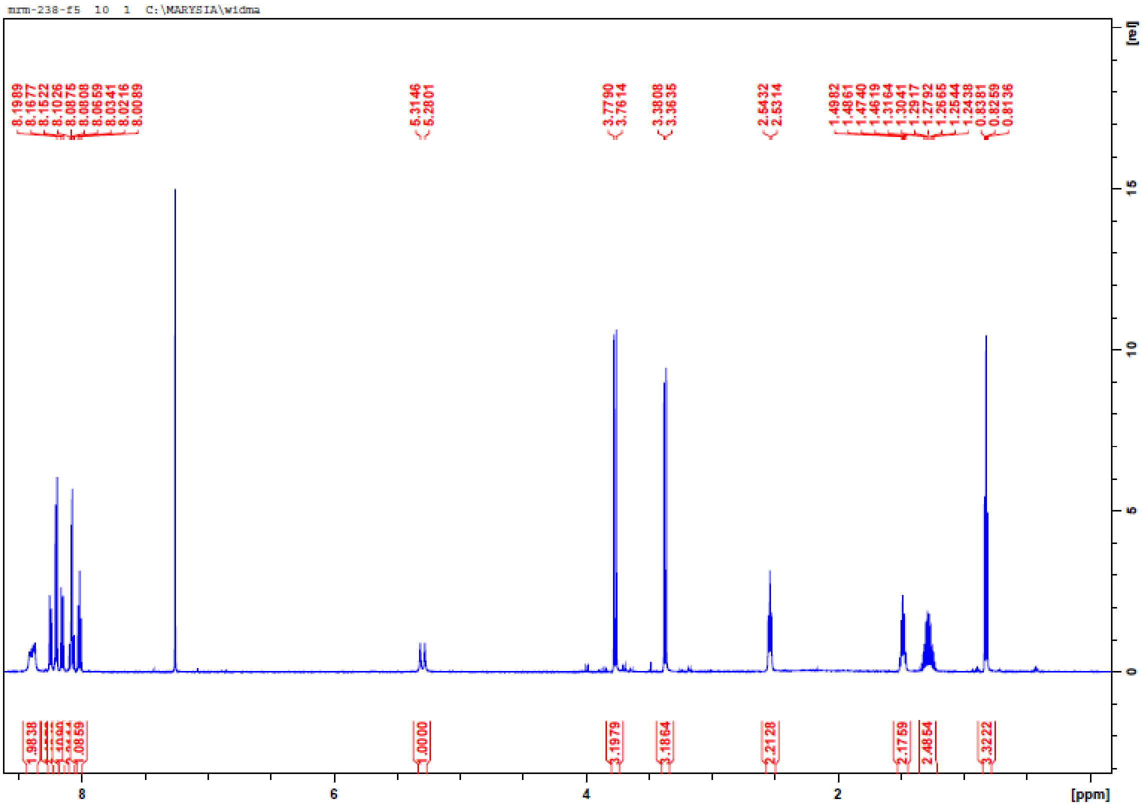

Dimethyl *N*-(1-propyl)amino(pyren-1-yl)methylphosphonate (**3Af)**

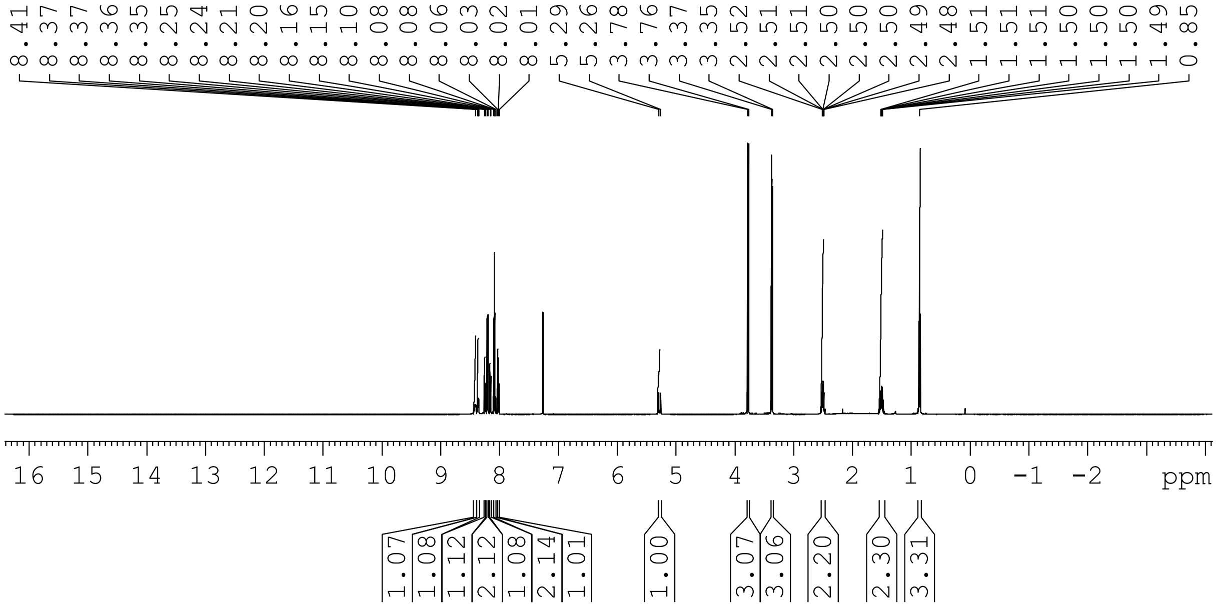

Dimethyl *N*-furfurylamino(pyren-1-yl)methylphosphonate (**3Ag**)

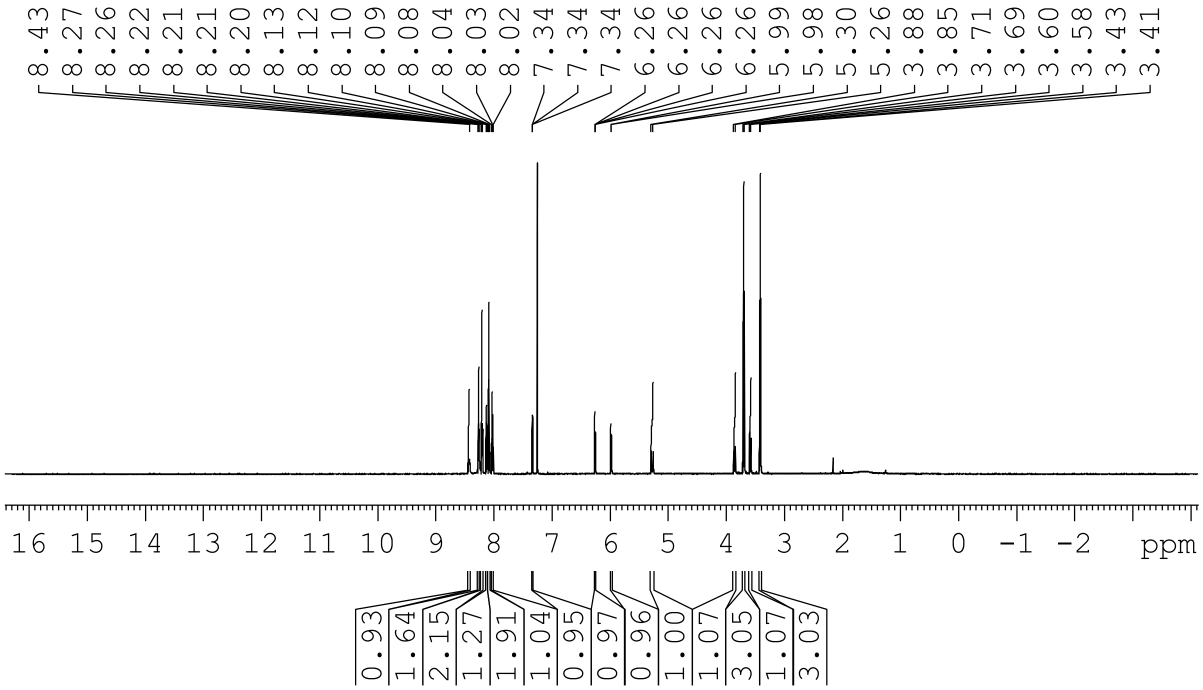

Dimethyl *N*-(3-methylphenyl)amino(pyren-1-yl)methylphosphonate (**3Ah**)

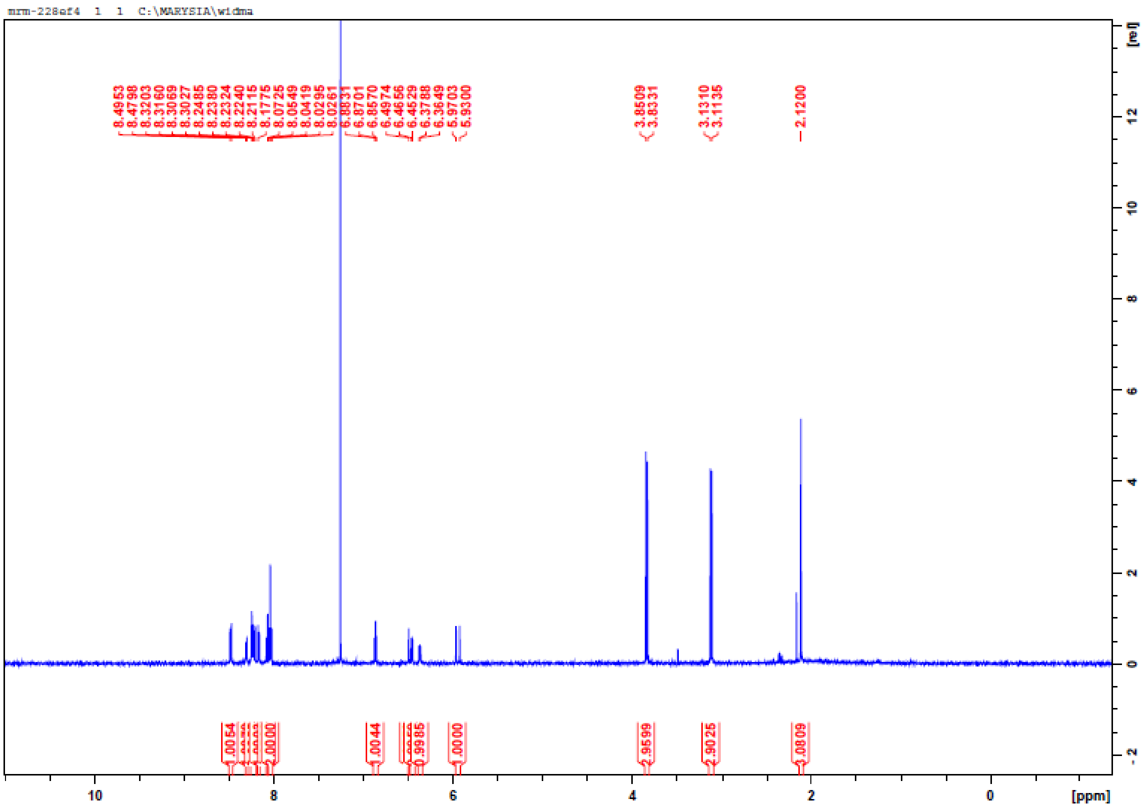

Dimethyl *N*-(*t*-butyl)amino(pyren-1-yl)methylphosphonate (**3Ai**)

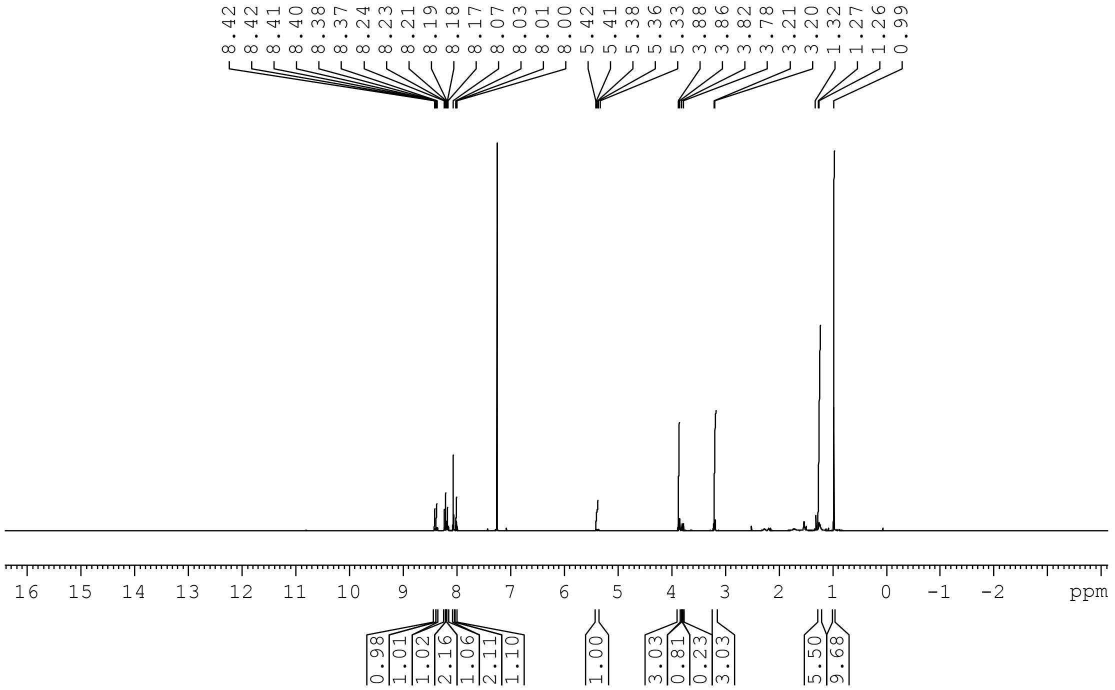

Dimethyl *N*-cyklohexylamino(pyren-1-yl)methylphosphonate (**3Aj**)

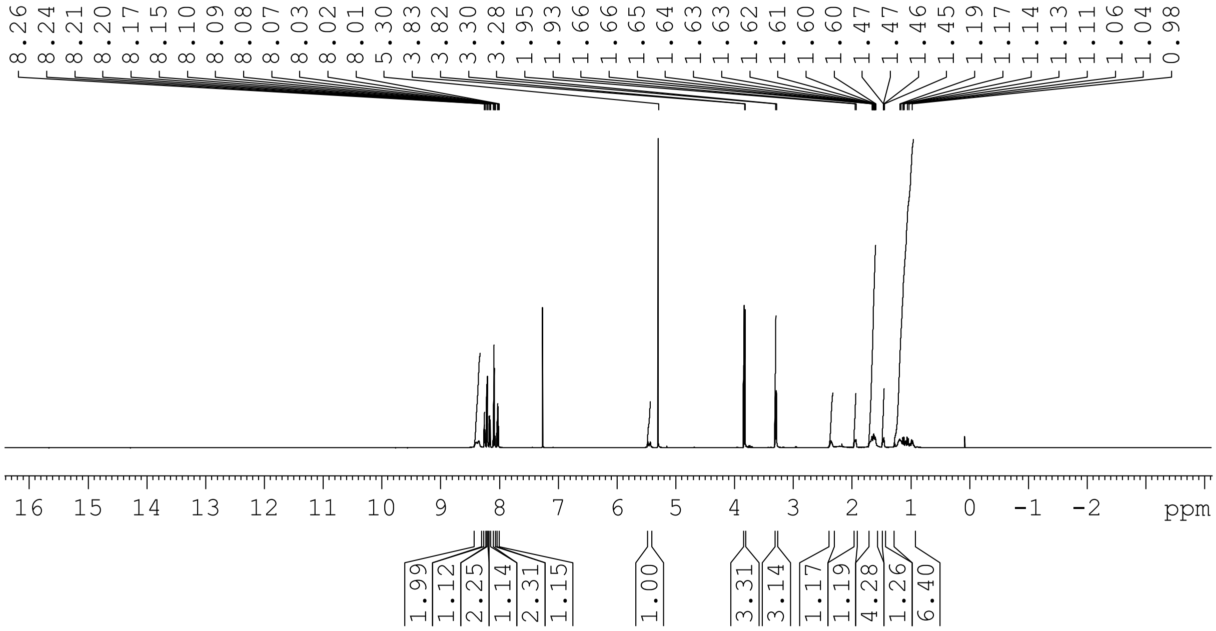

Diethyl *N*-benzylamino(pyren-1-yl)methylphosphonate (**3Ba**)

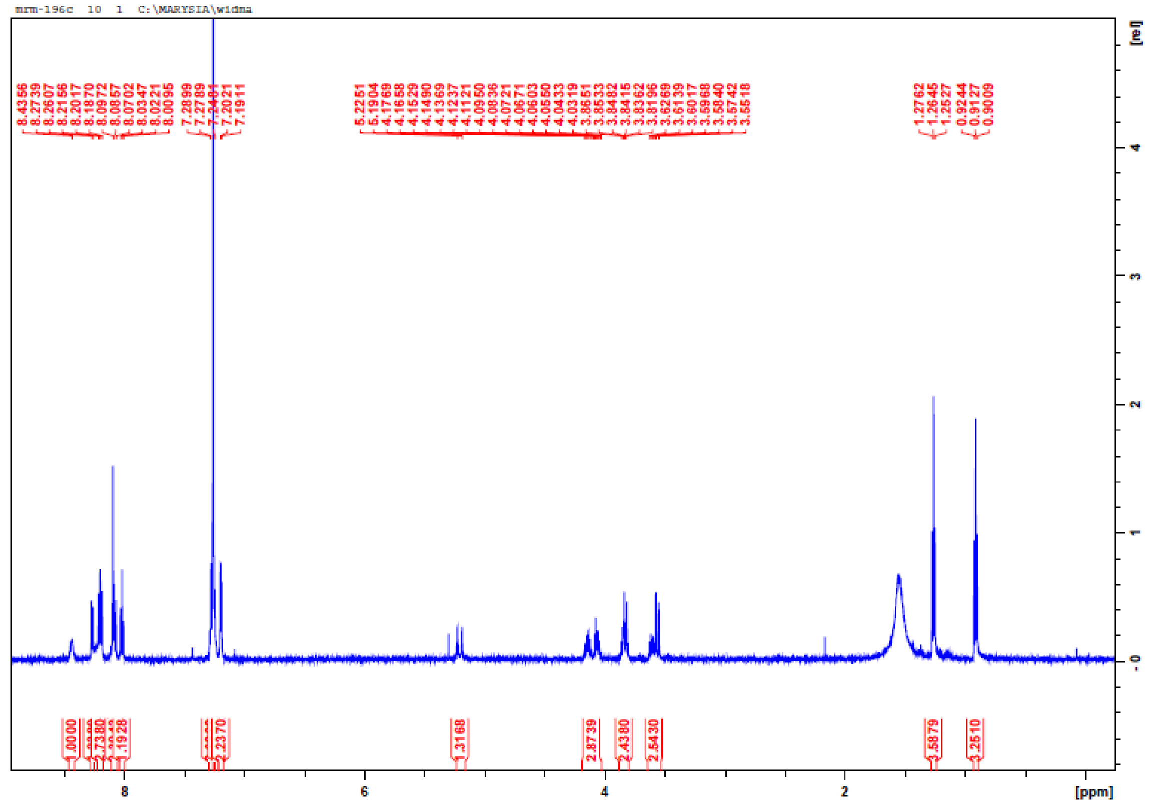

Diethyl *N*-phenylamino(pyren-1-yl)methylphosphonate (**3Bb**)

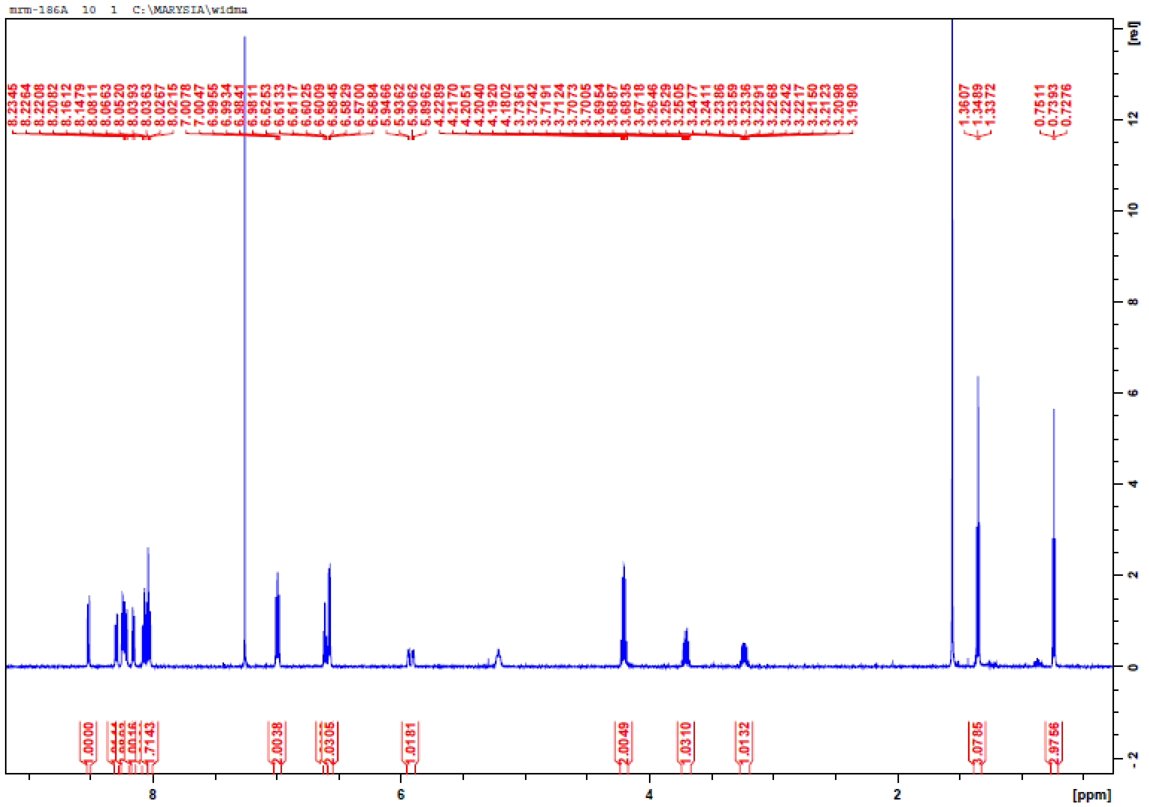

Diethyl *N*-(4-methylphenyl)amino(pyren-1-yl)methylphosphonate (**3Bc**)

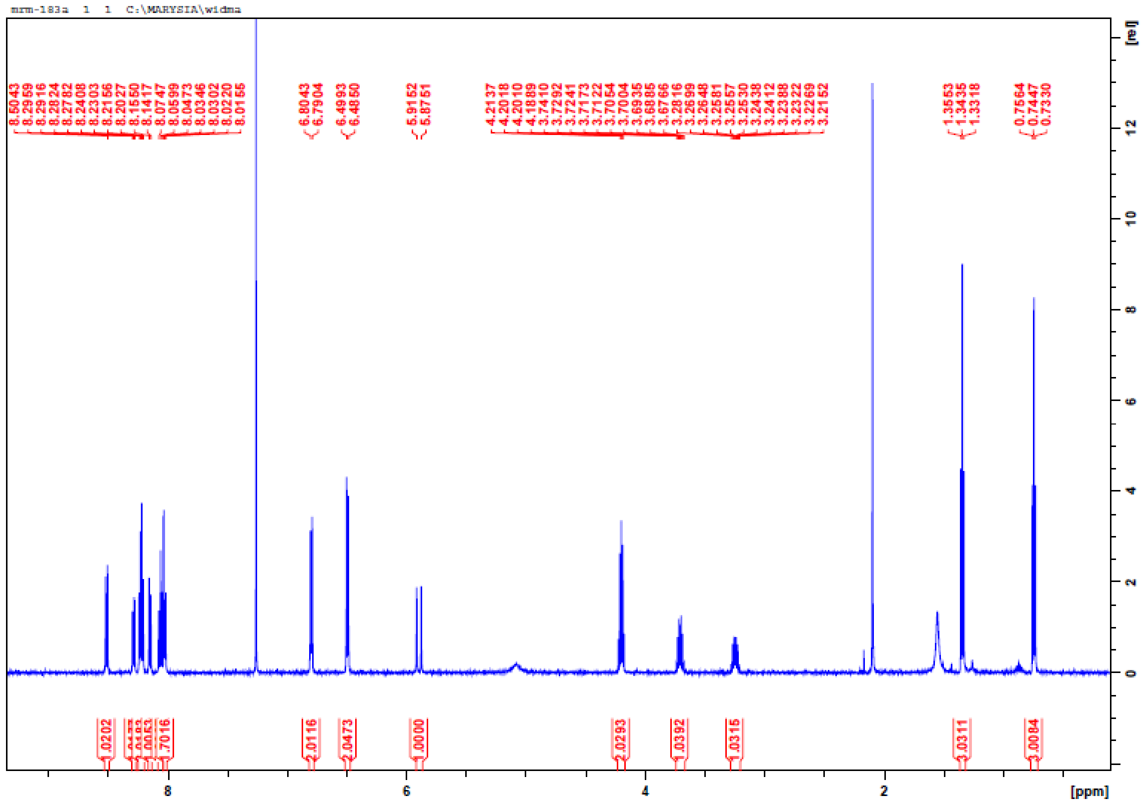

Diethyl *N*-(4-methoxyphenyl)amino(pyren-1-yl)methylphosphonate (**3Bd**)

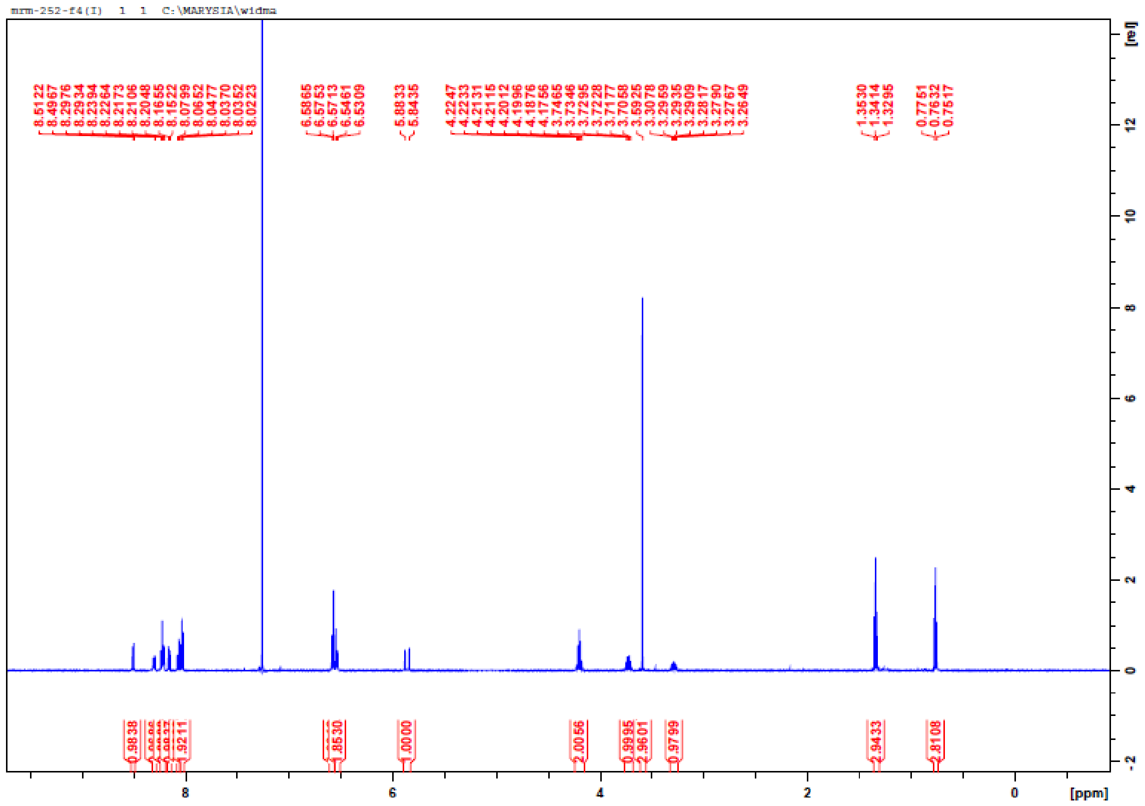

Diethyl *N*-(1-butyl)amino(pyren-1-yl)methylphosphonate (**3Be**)

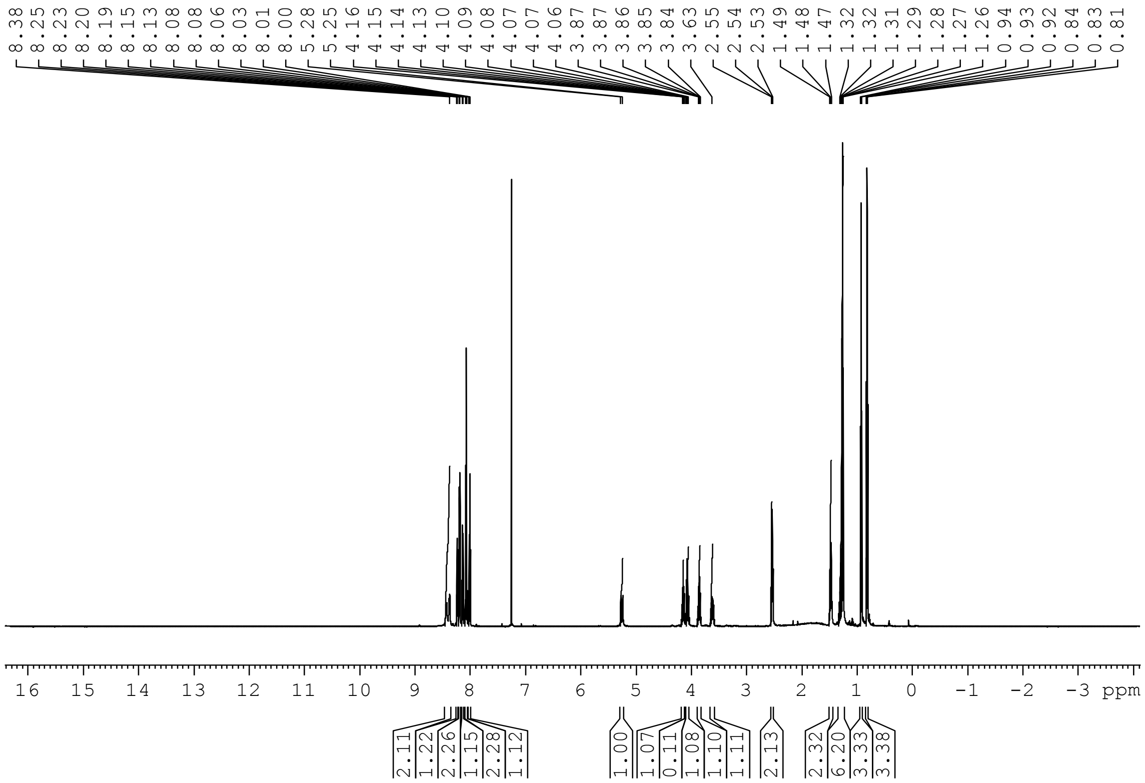

Dibenzyl *N*-benzylamino(pyren-1-yl)methylphosphonate (**3Ca**)

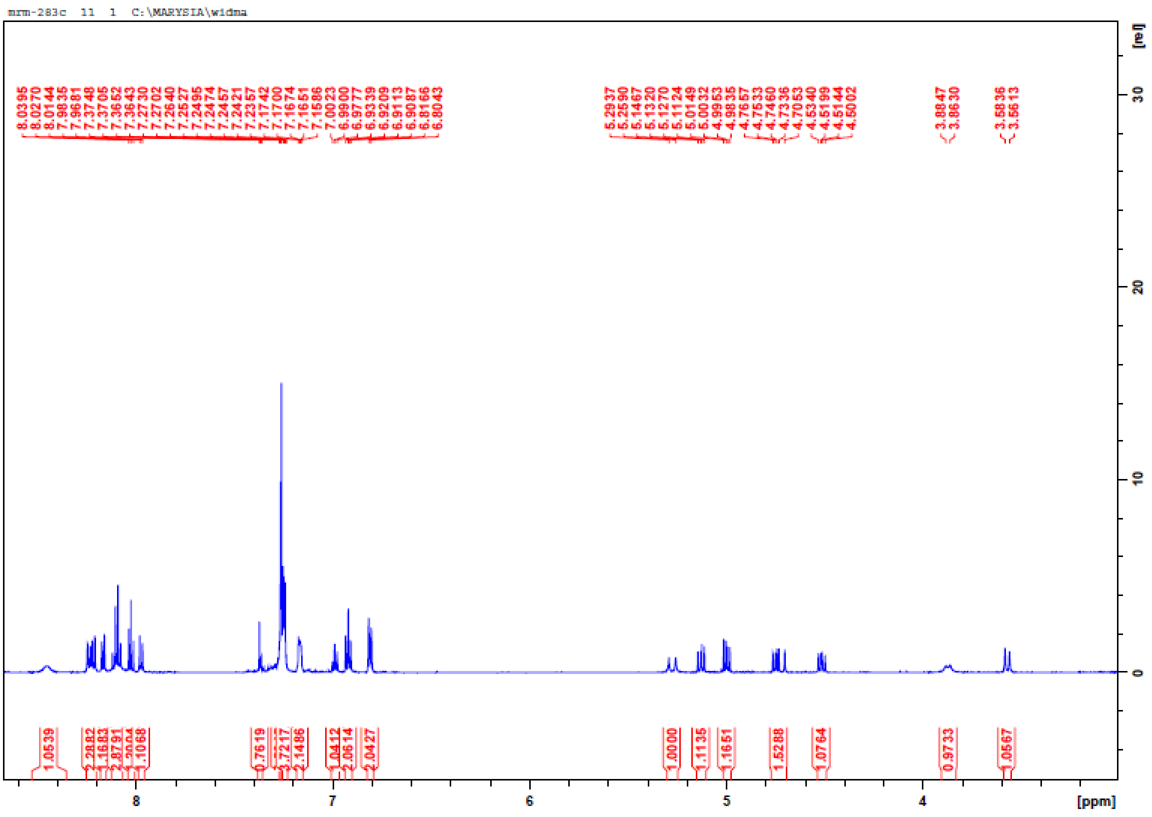

Dibenzyl *N*-phenylamino(pyren-1-yl)methylphosphonate (**3Cb**)

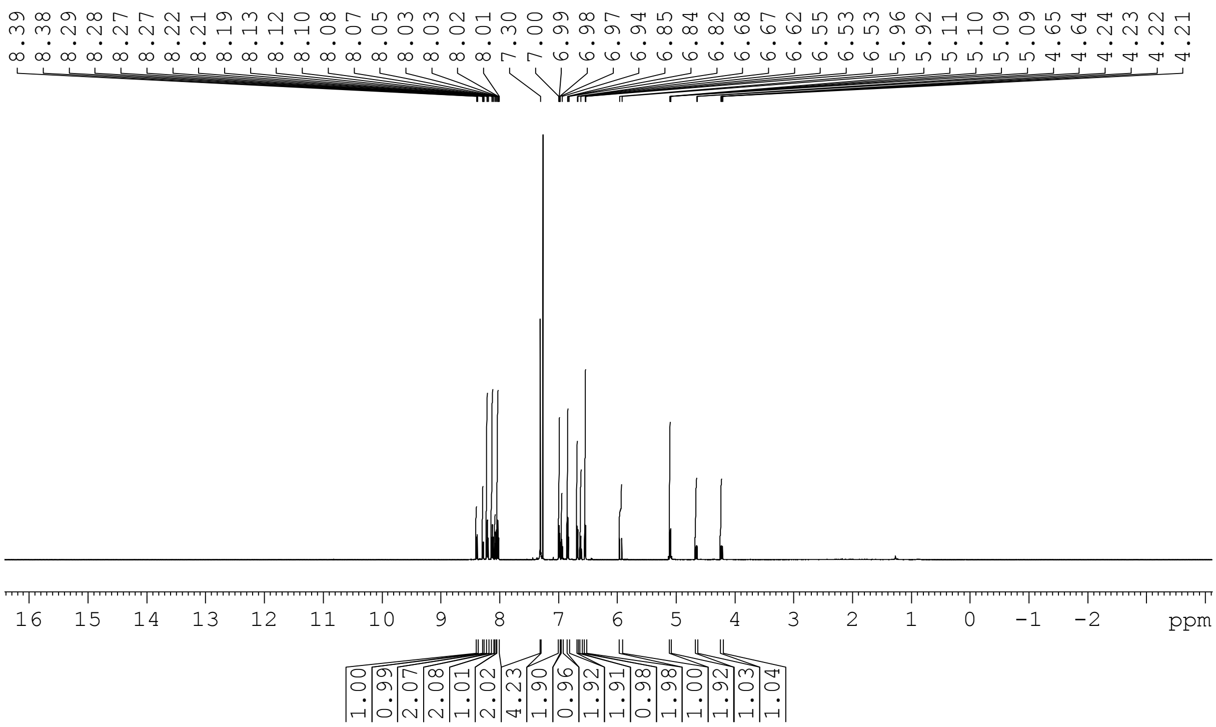

Dibenzyl *N*-(4-methylphenyl)amino(pyren-1-yl)methylphosphonate (**3Cc**)

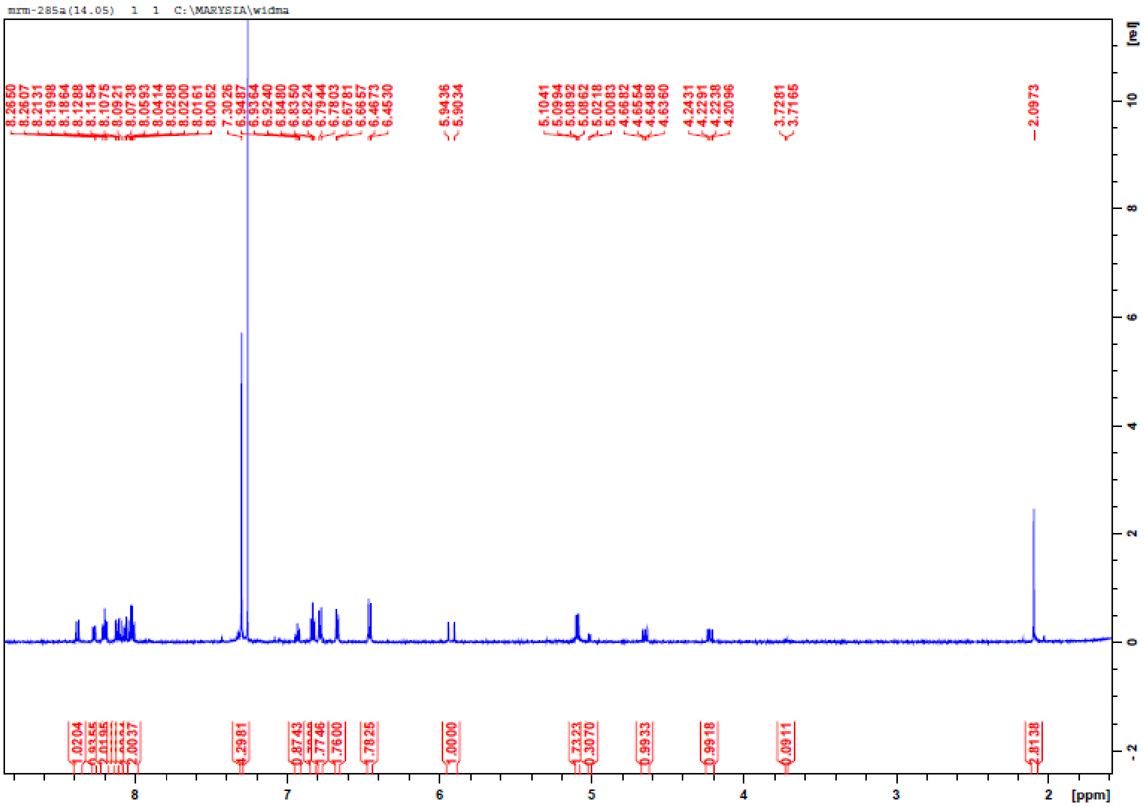

Dibenzyl *N*-(4-methoxyphenyl)amino(pyren-1-yl)methylphosphonate (**3Cd**)

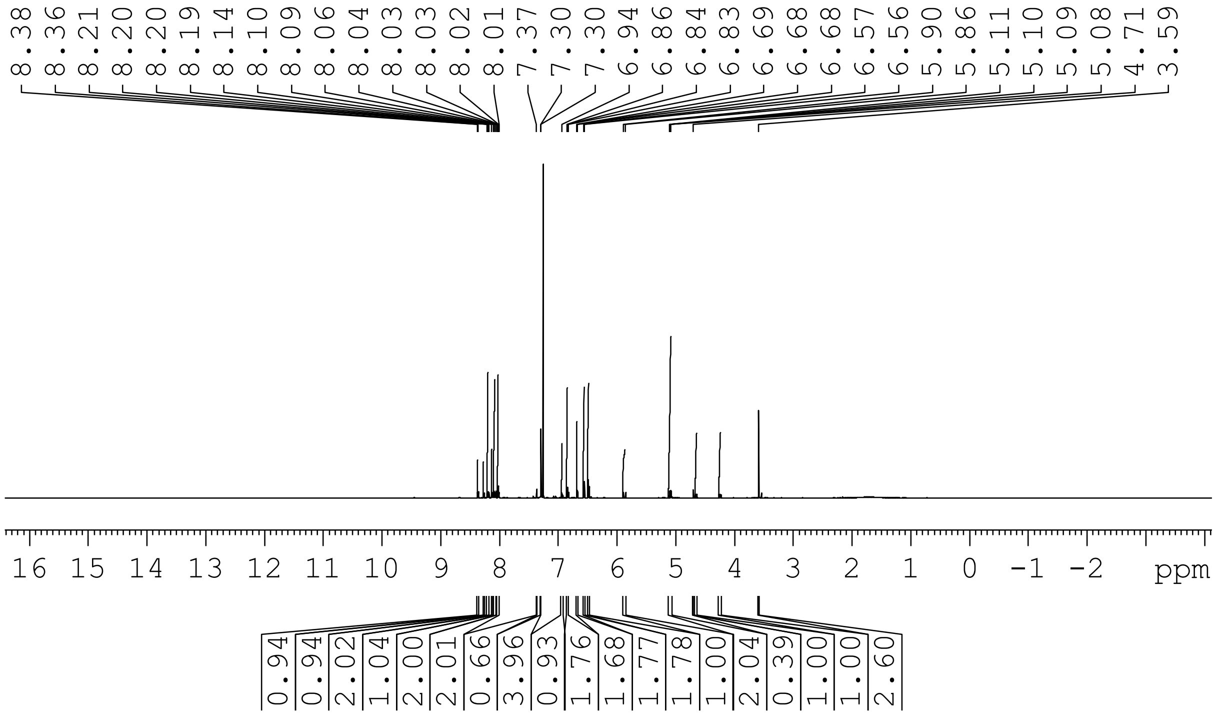

Dibenzyl *N*-furfurylamino(pyren-1-yl)methylphosphonate (**3Cg**)

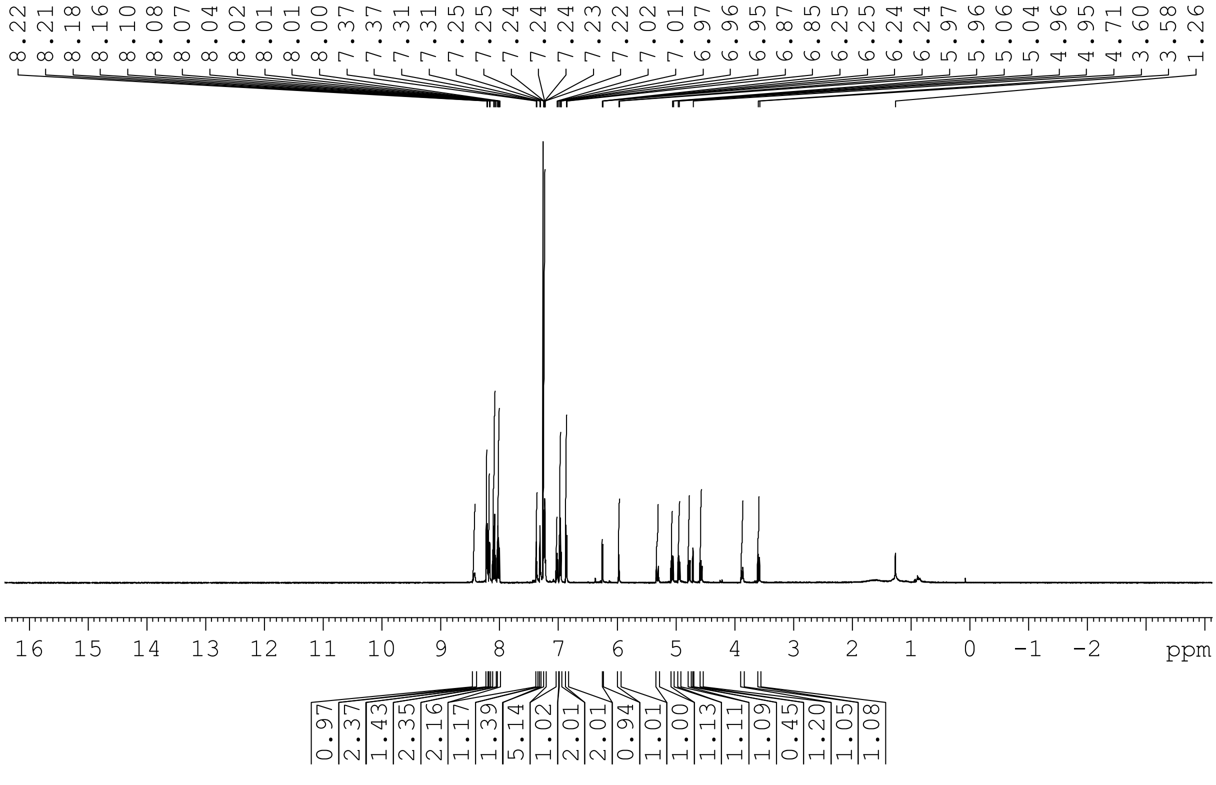

N-benzylamino(pyren-1-yl)methylphosphonic Acid (**4a**)

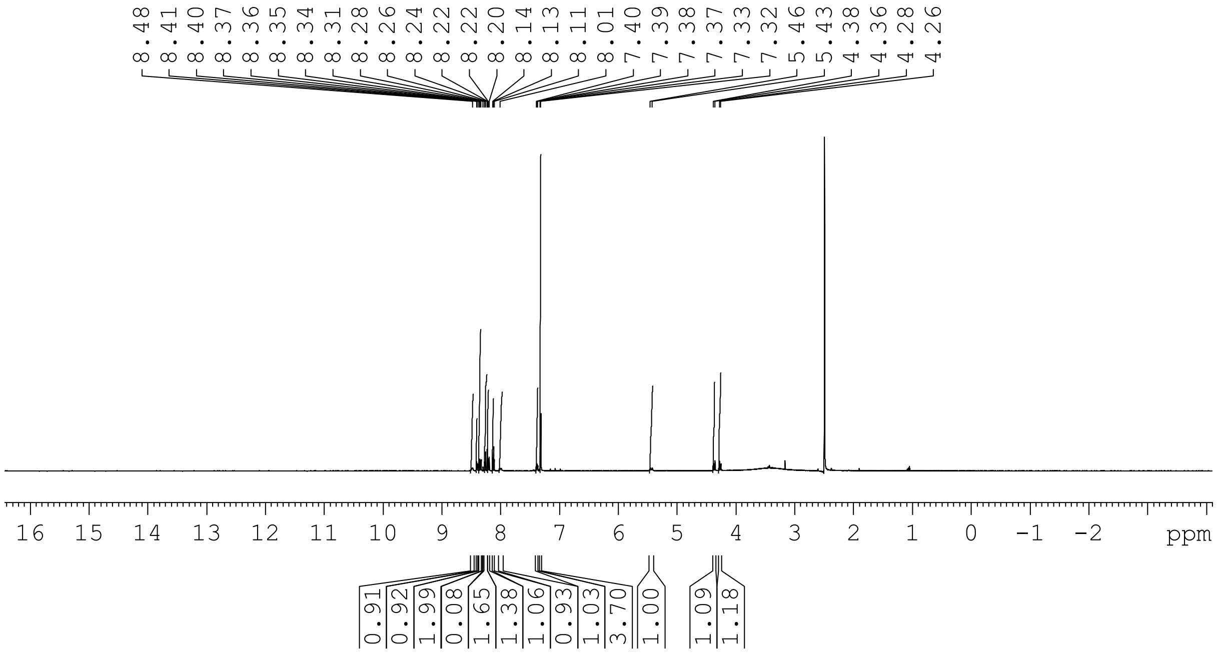

N-(p-methylphenyl)amino(pyren-1-yl)methylphosphonic Acid (**4c**)

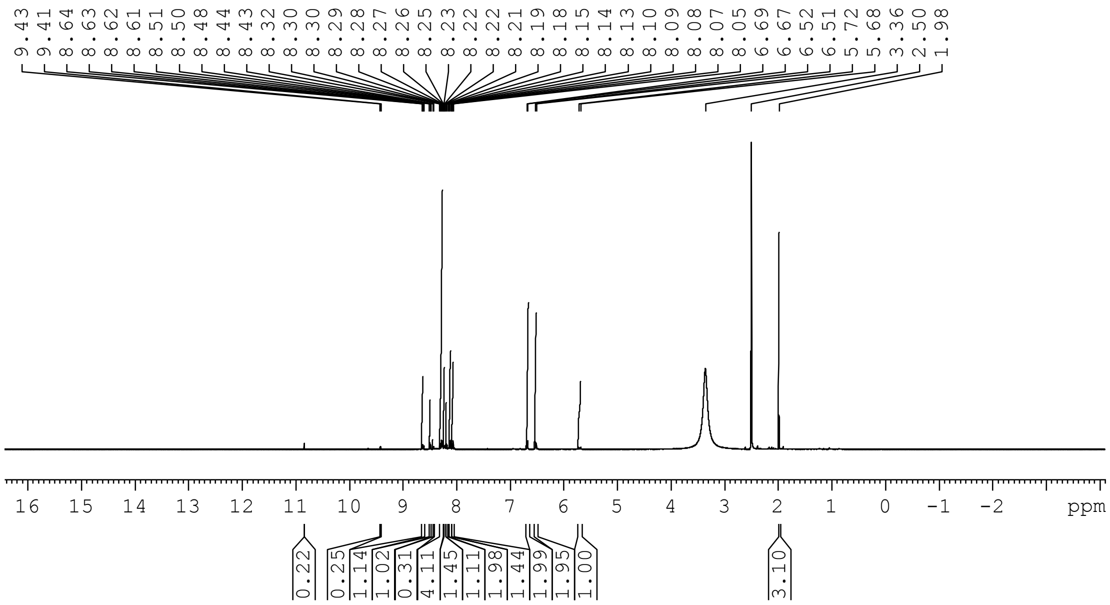

Dimethyl hydroxy(pyren-1-yl)methylphosphonate (**5A**)

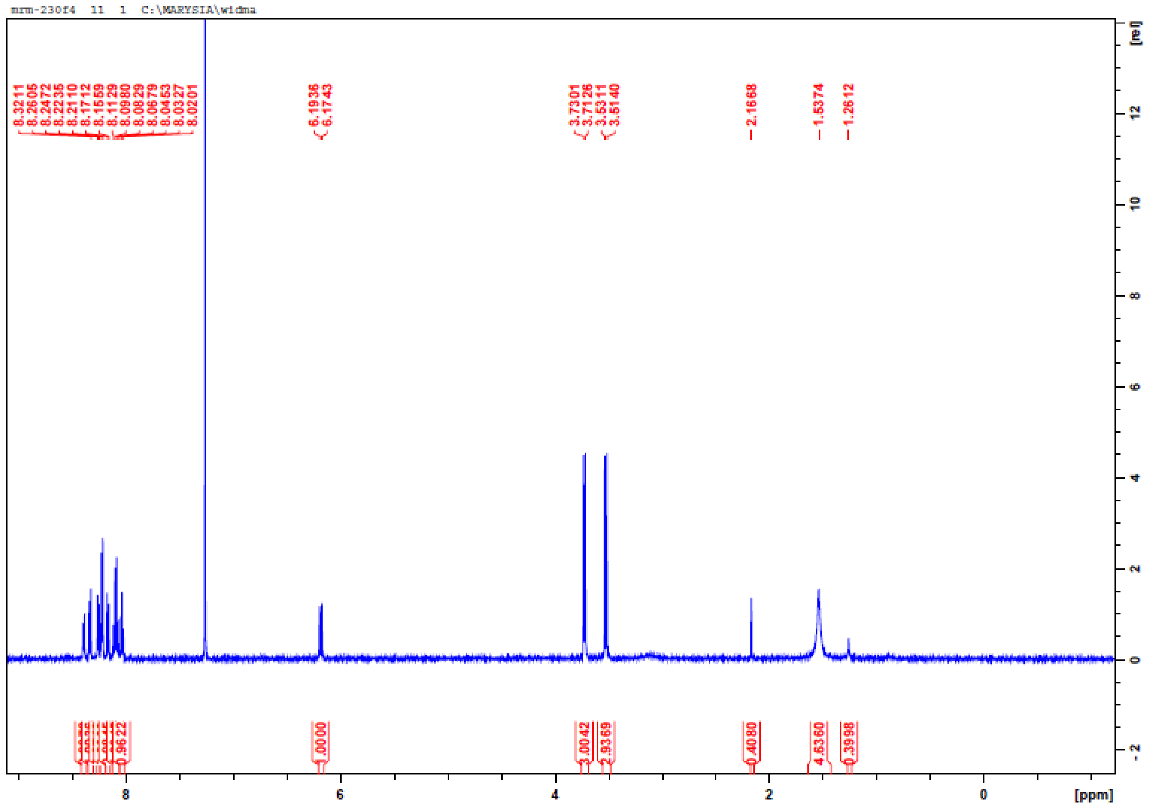

^{13}C NMR spectra of studied compounds 3, 4 and 5A

Dimethyl *N*-benzylamino(pyren-1-yl)methylphosphonate (**3Aa**)

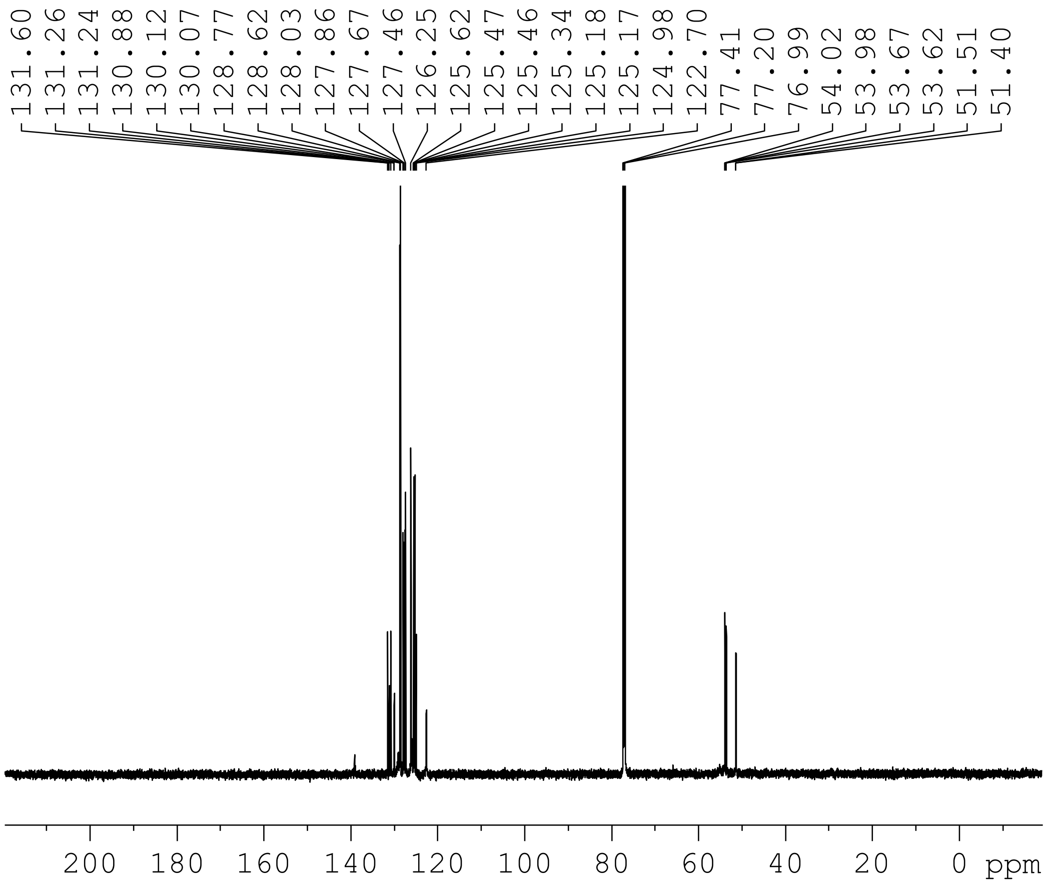

Dimethyl *N*-phenylamino(pyren-1-yl)methylphosphonate (**3Ab**)

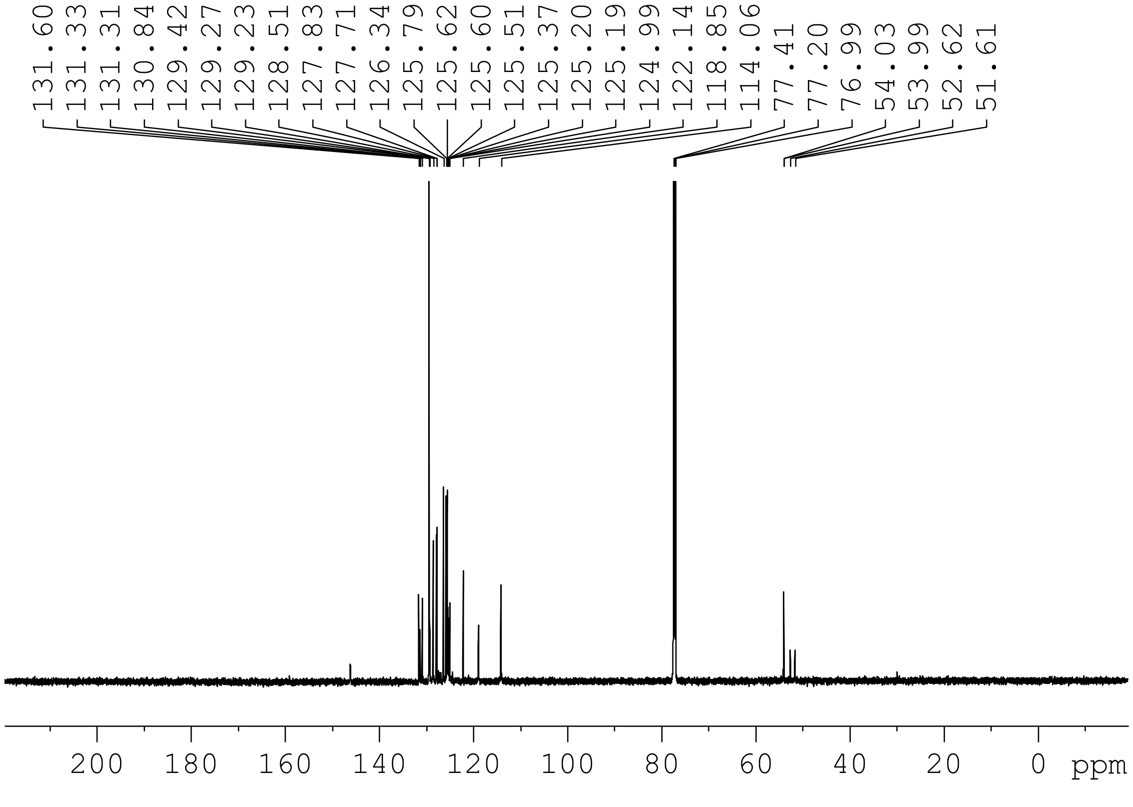

Dimethyl *N*-(4-methylphenyl)amino(pyren-1-yl)methylphosphonate (**3Ac**)

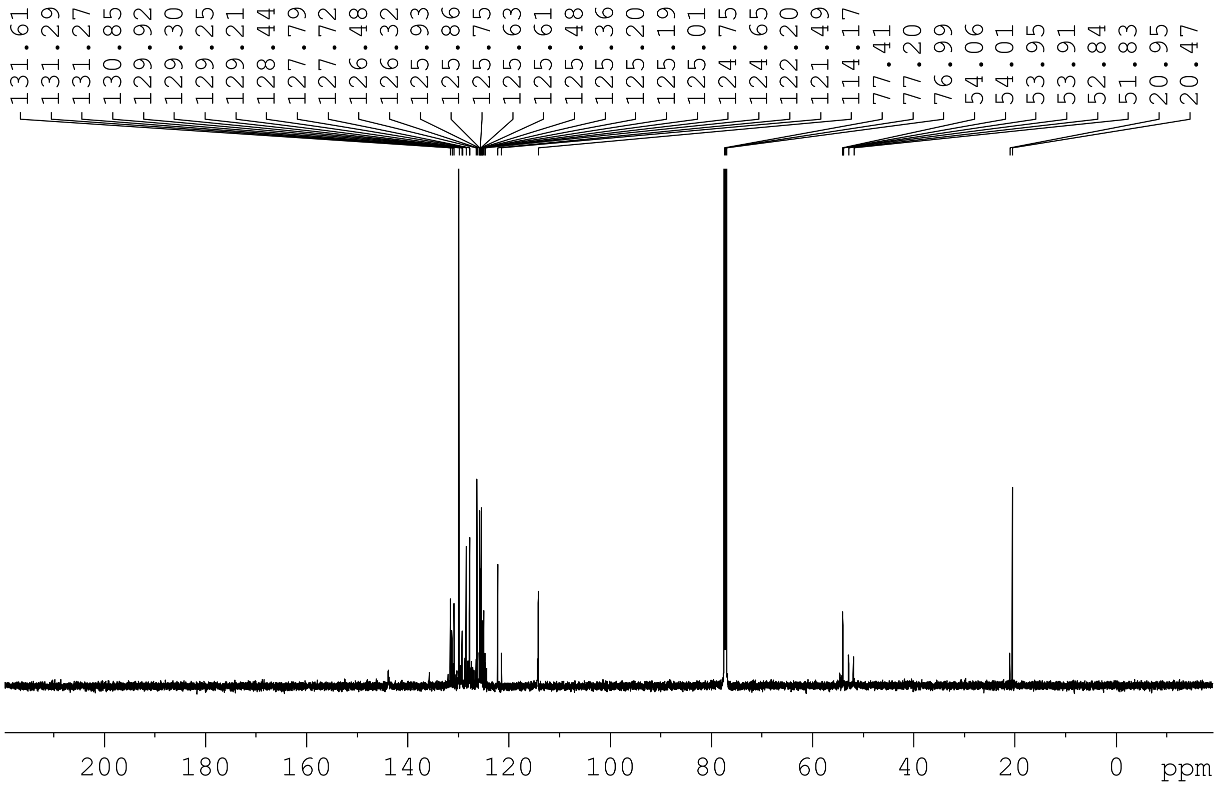

Dimethyl *N*-(4-methoxyphenyl)amino(pyren-1-yl)methylphosphonate (**3Ad**)

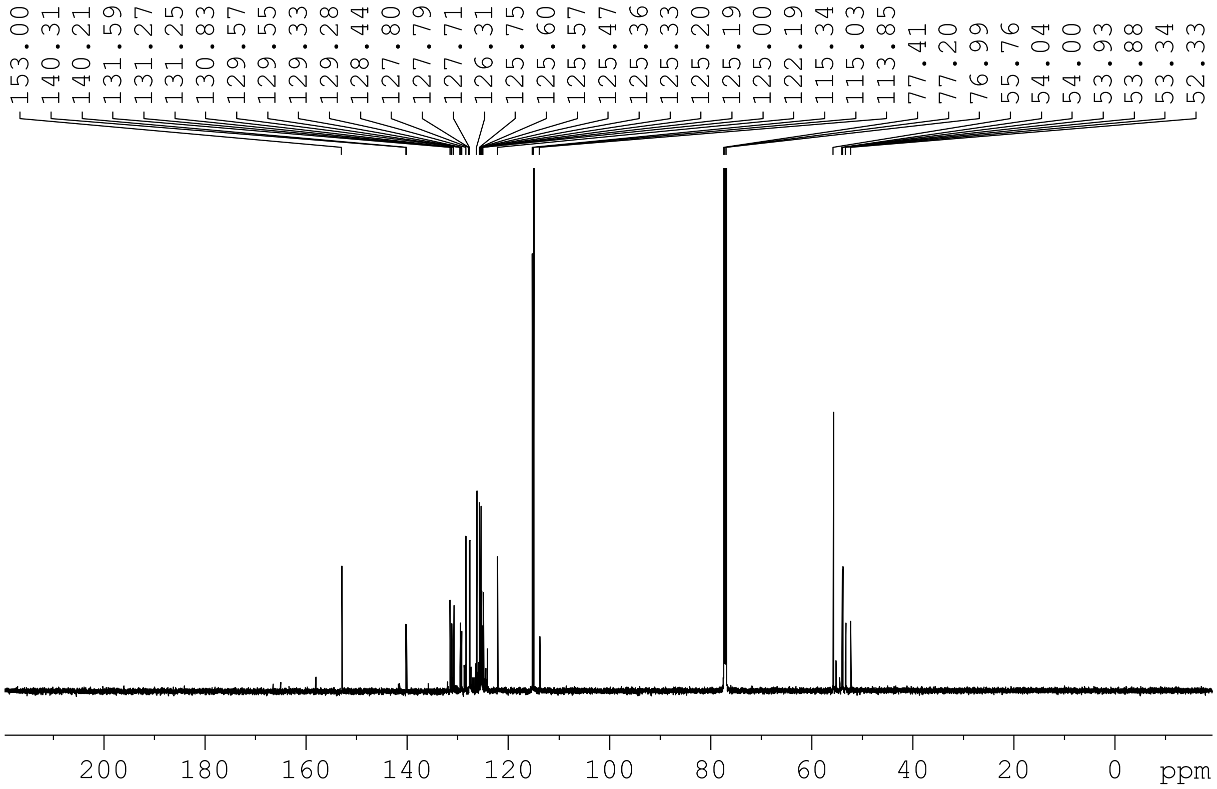

Dimethyl *N*-(1-butyl)amino(pyren-1-yl)methylphosphonate (**3Ae**)

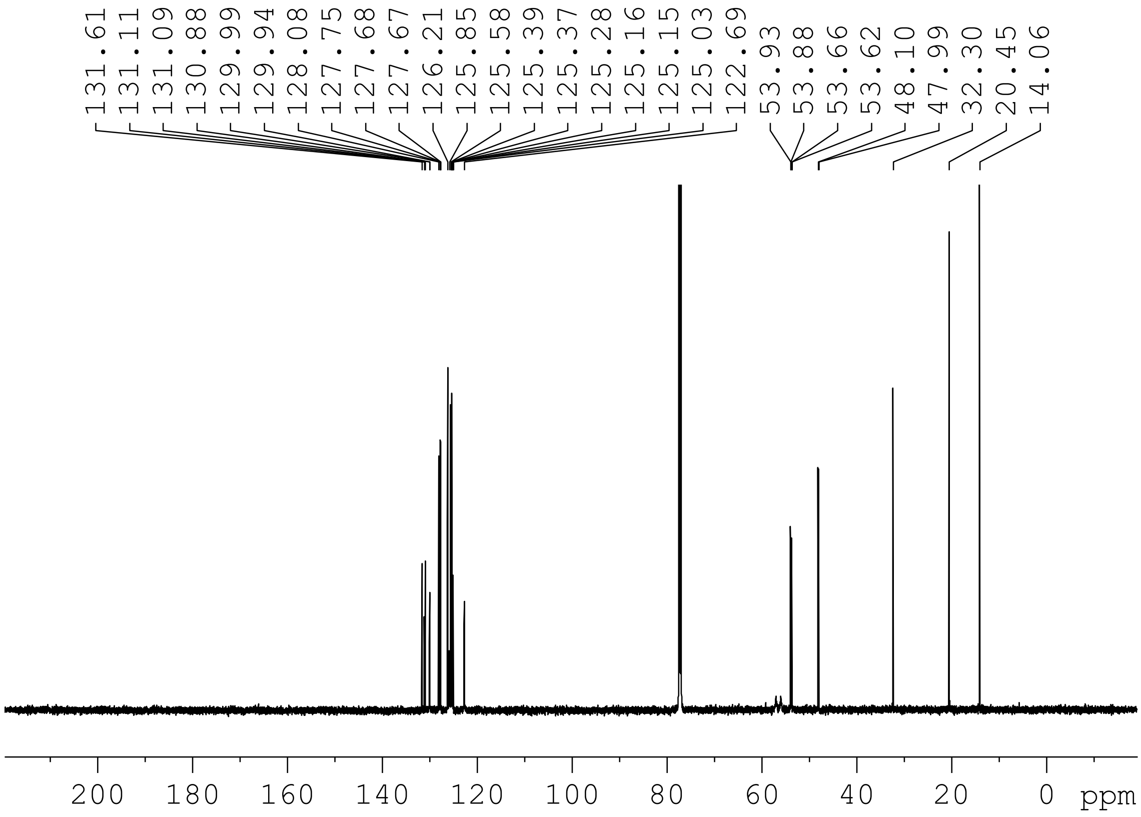

Dimethyl *N*-(1-propyl)amino(pyren-1-yl)methylphosphonate (**3Af**)

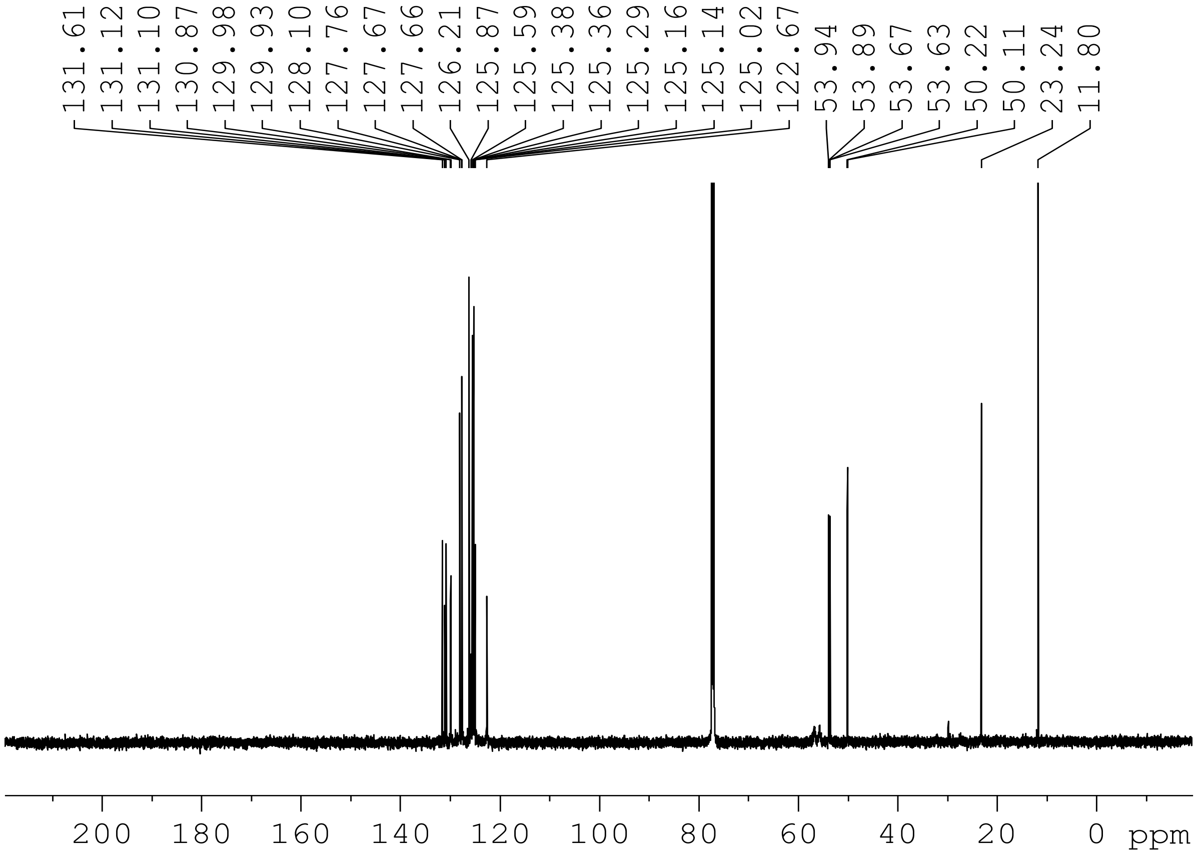

Dimethyl *N*-furfurylamino(pyren-1-yl)methylphosphonate (**3Ag**)

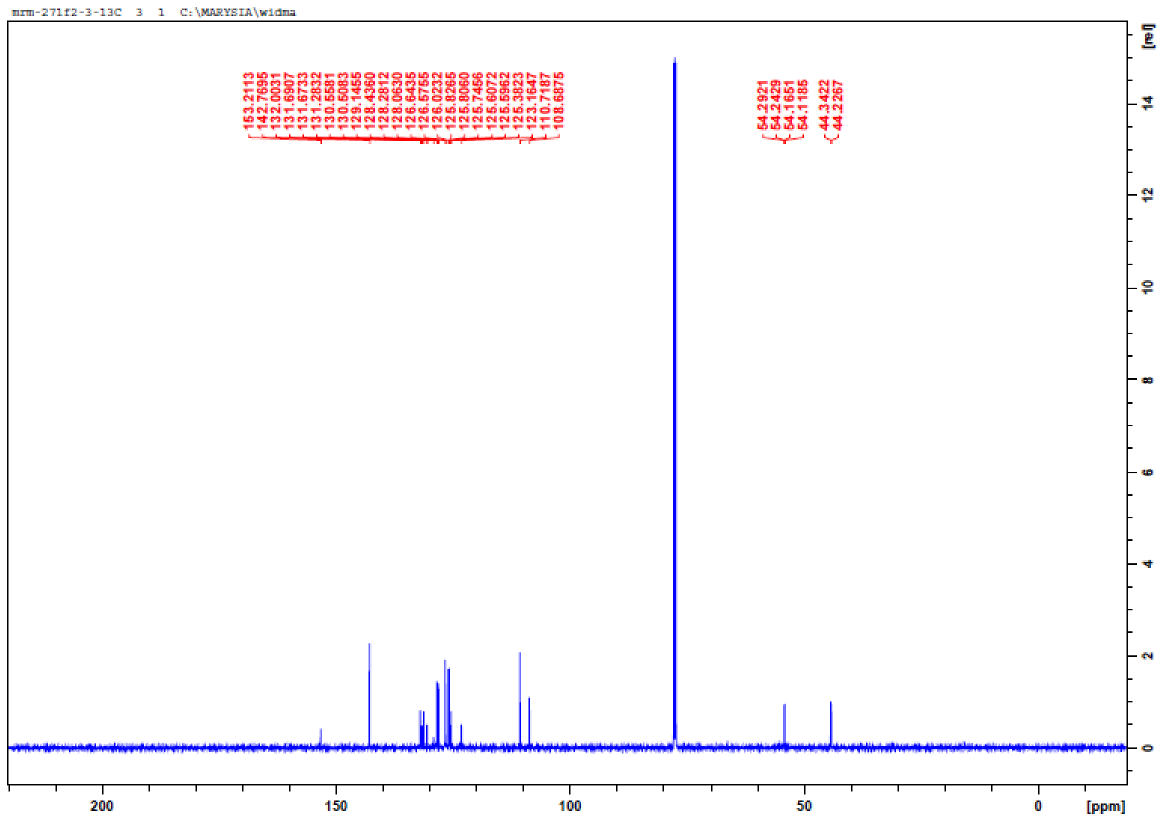

Dimethyl *N*-(3-methylphenyl)amino(pyren-1-yl)methylphosphonate (**3Ah**)

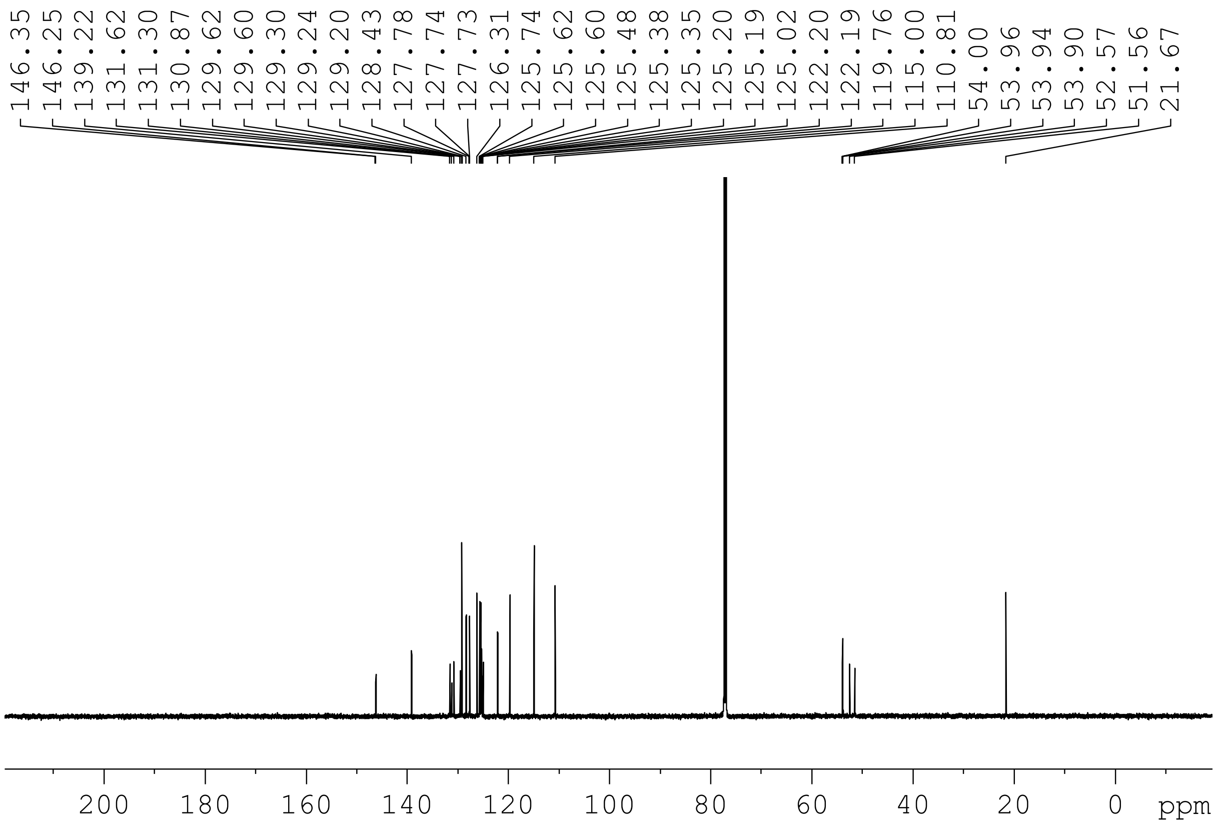

Dimethyl *N*-(*t*-butyl)amino(pyren-1-yl)methylphosphonate (**3Ai**)

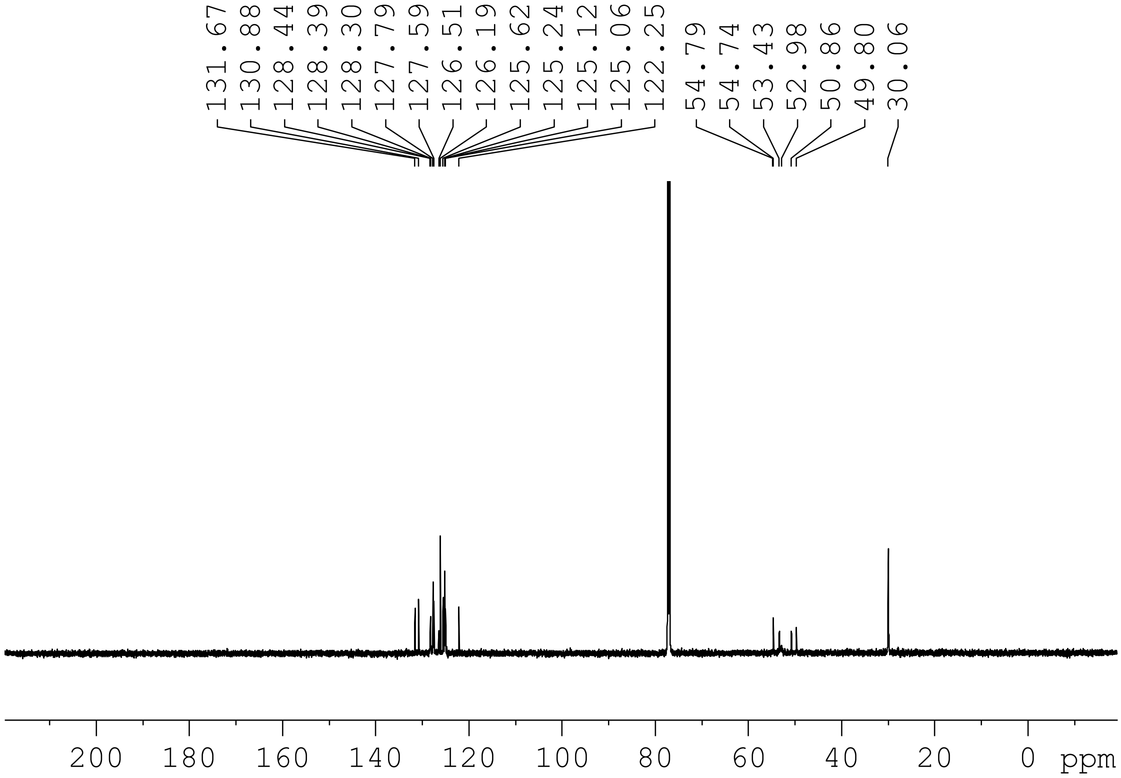

Dimethyl *N*-cyklohexylamino(pyren-1-yl)methylphosphonate (**3Aj**)

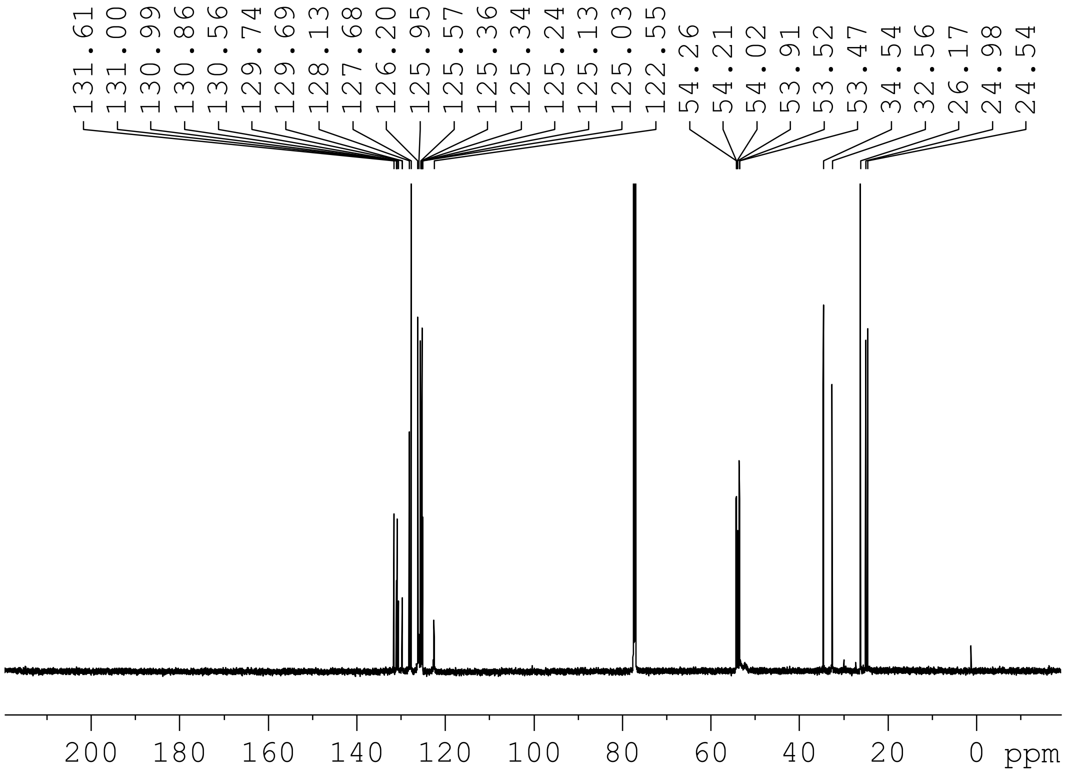

Diethyl *N*-benzylamino(pyren-1-yl)methylphosphonate (**3Ba**)

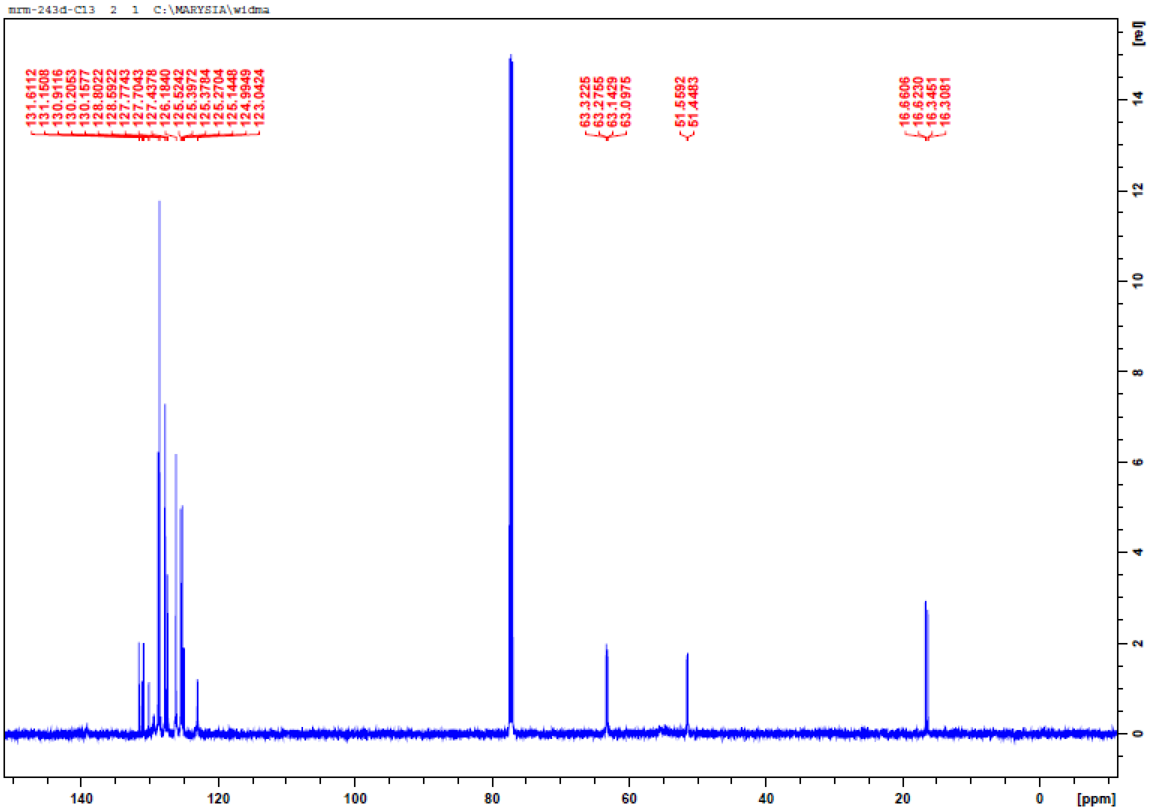

Diethyl *N*-phenylamino(pyren-1-yl)methylphosphonate (**3Bb**)

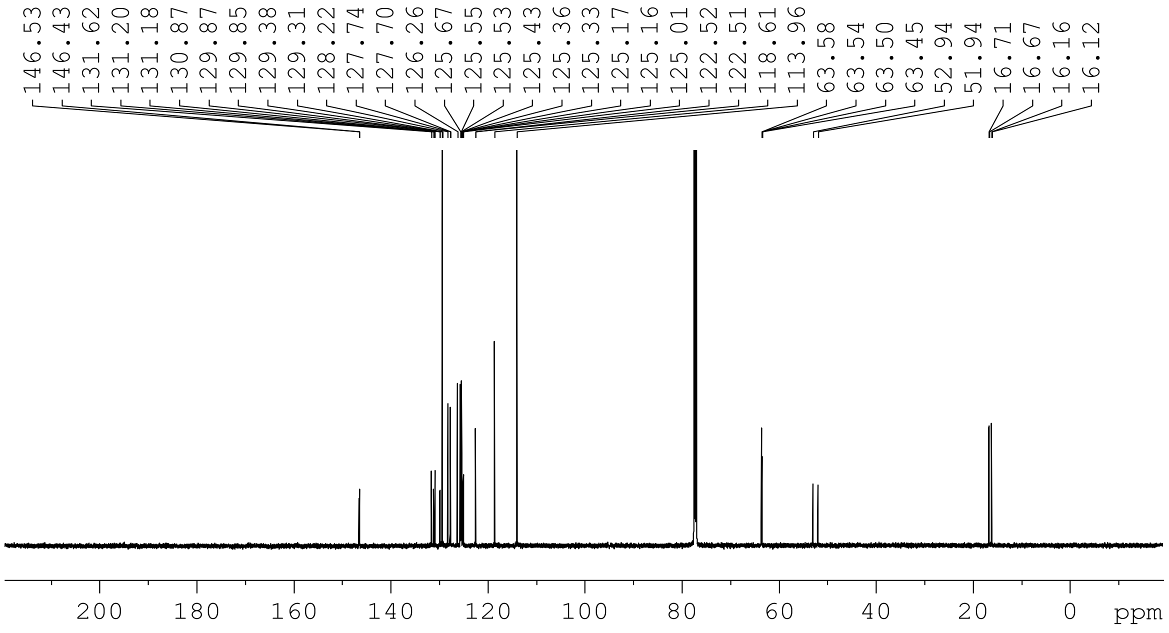

Diethyl *N*-(4-methylphenyl)amino(pyren-1-yl)methylphosphonate (**3Bc**)

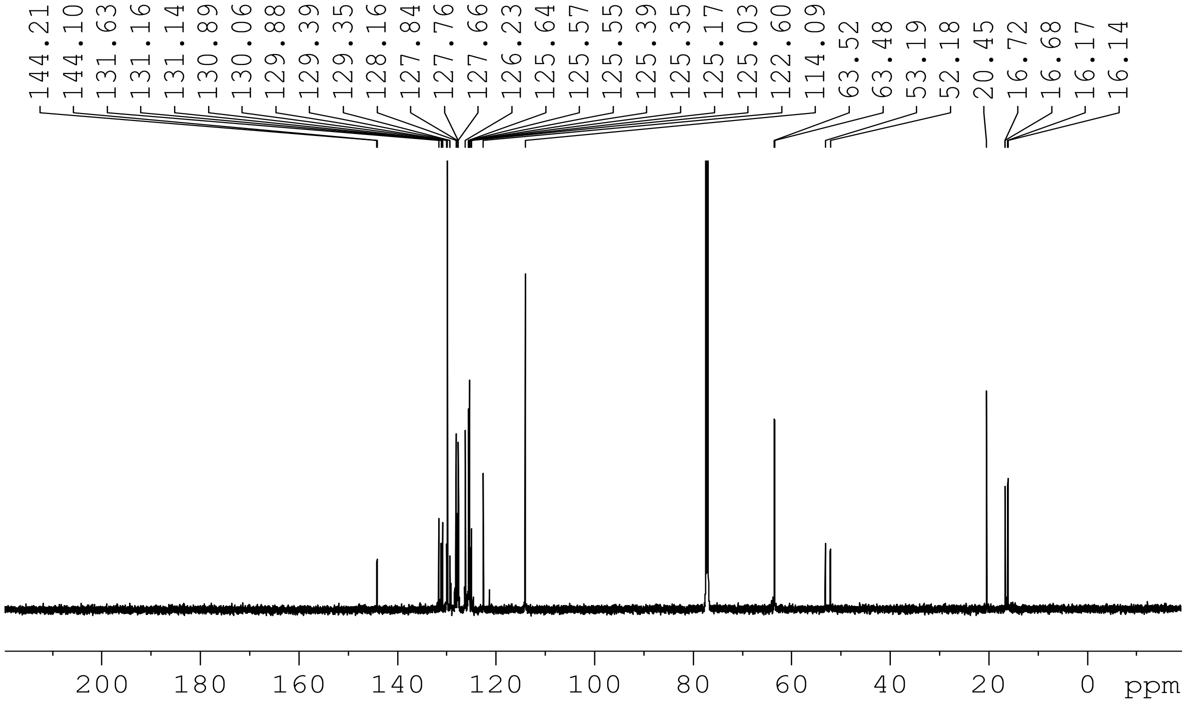

Diethyl *N*-(4-methoxyphenyl)amino(pyren-1-yl)methylphosphonate (**3Bd**)

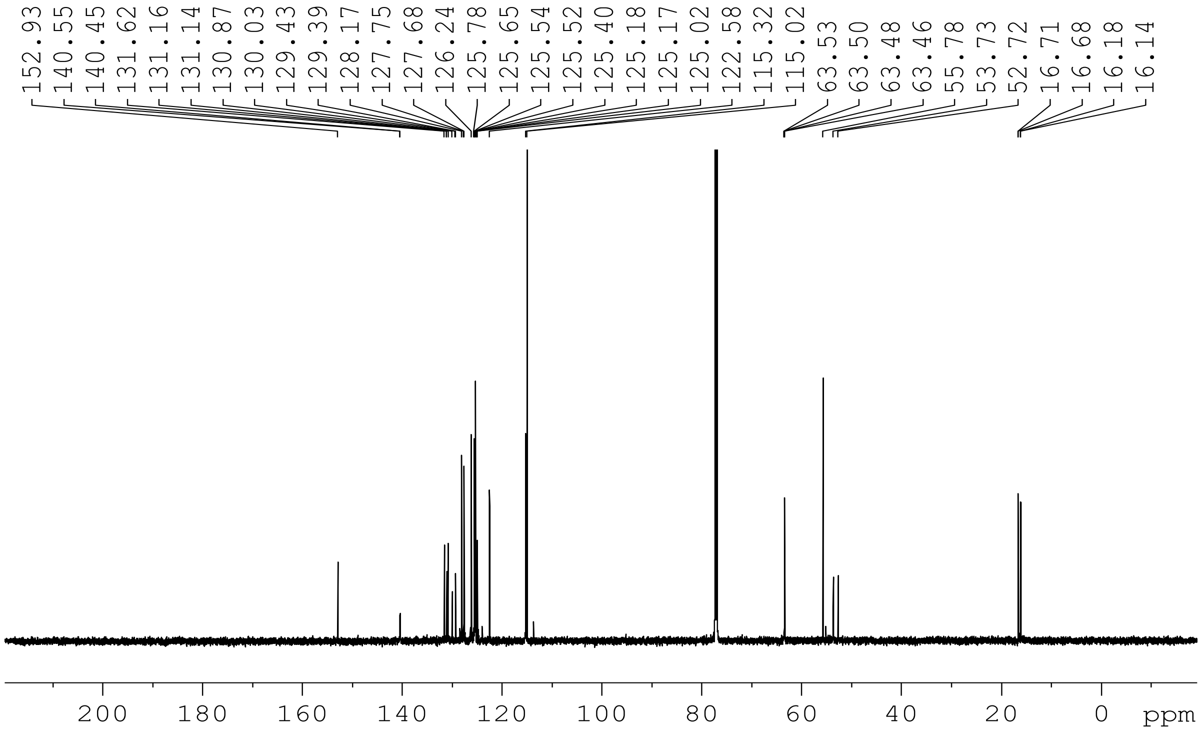

Diethyl *N*-(1-butyl)amino(pyren-1-yl)methylphosphonate (**3Be**)

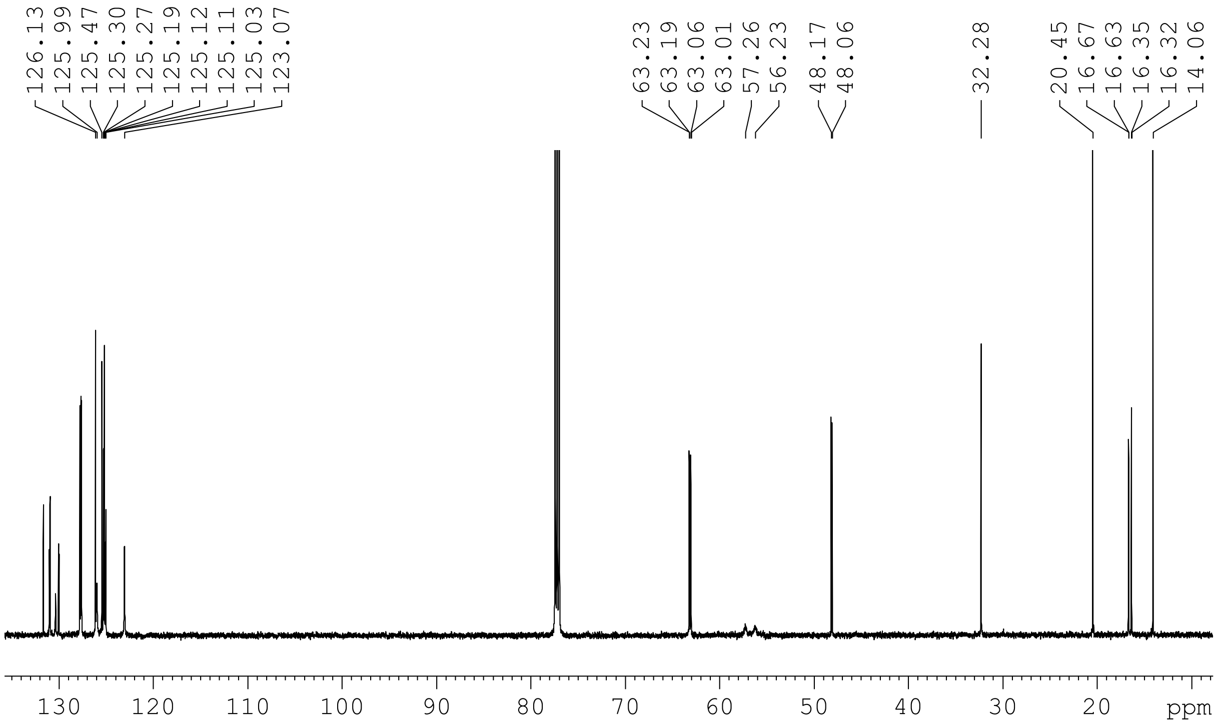

Dibenzyl *N*-benzylamino(pyren-1-yl)methylphosphonate (**3Ca**)

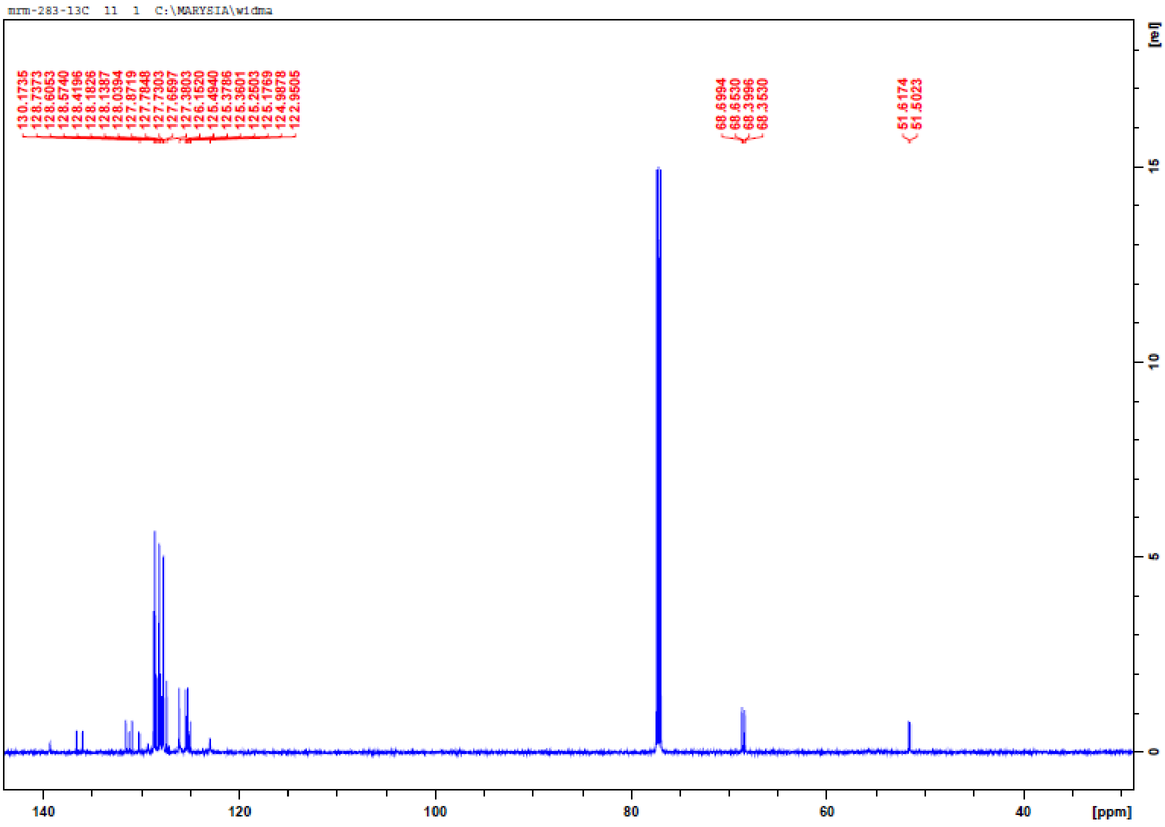

Dibenzyl *N*-phenylamino(pyren-1-yl)methylphosphonate (**3Cb**)

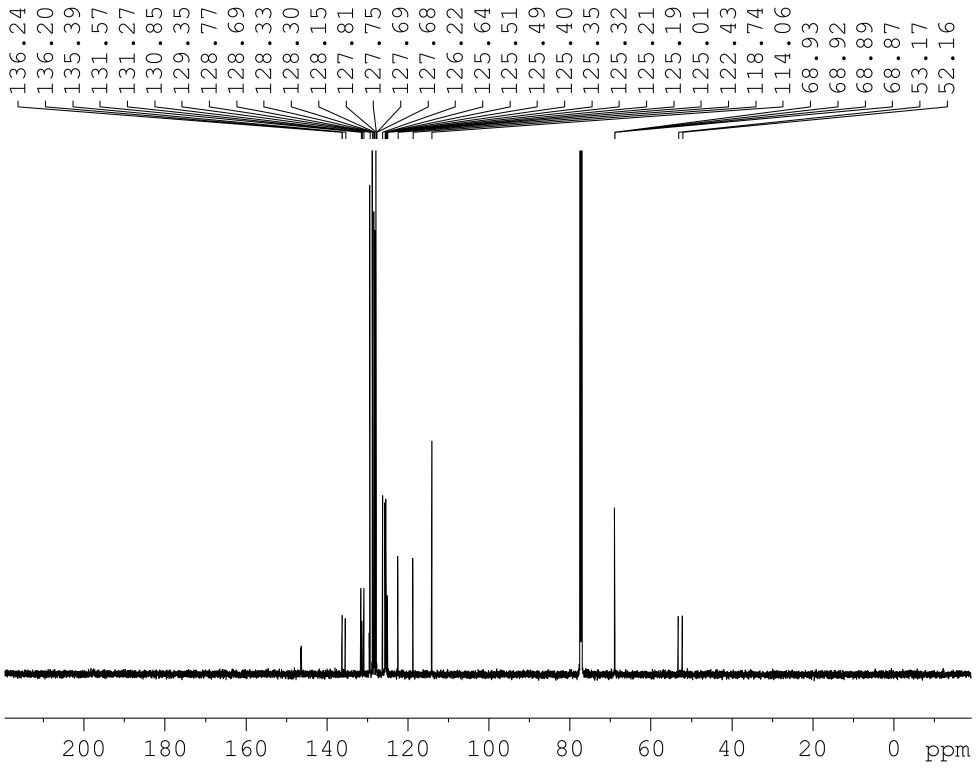

Dibenzyl *N*-(4-methylphenyl)amino(pyren-1-yl)methylphosphonate (**3Cc**)

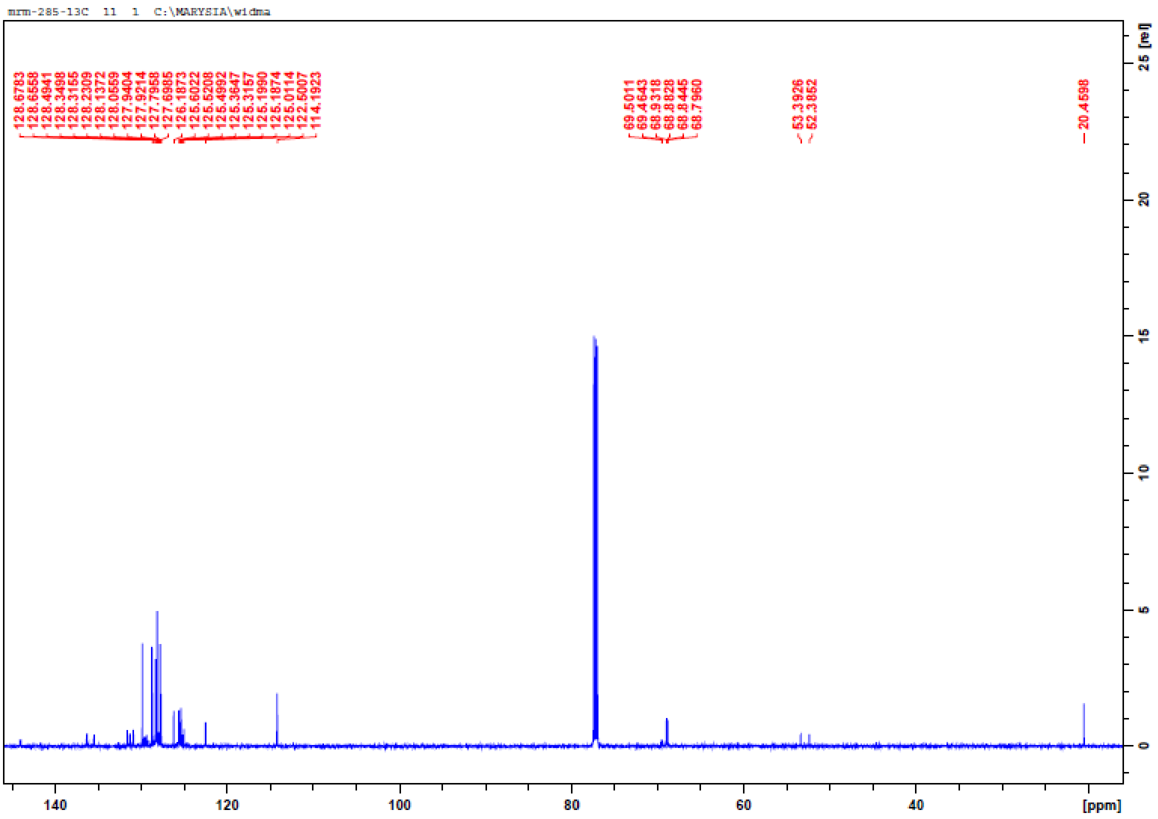

Dibenzyl *N*-(4-methoxyphenyl)amino(pyren-1-yl)methylphosphonate (**3Cd**)

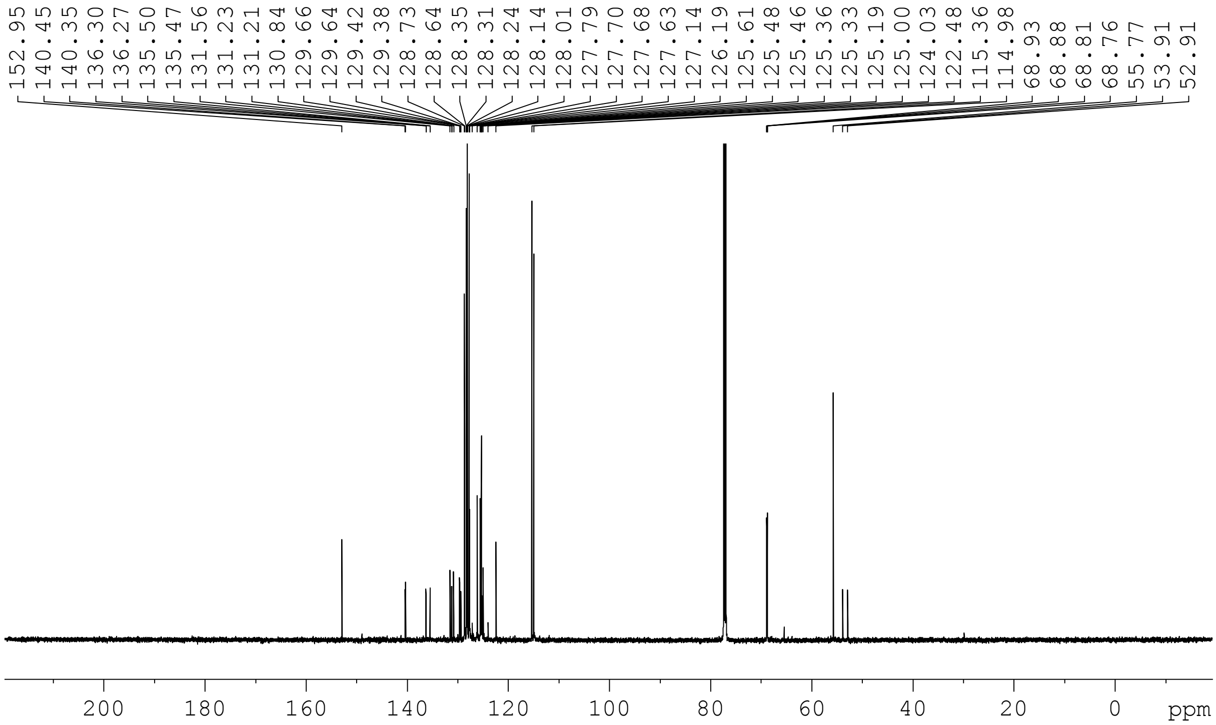

Dibenzyl *N*-furfurylamino(pyren-1-yl)methylphosphonate (**3Cg**)

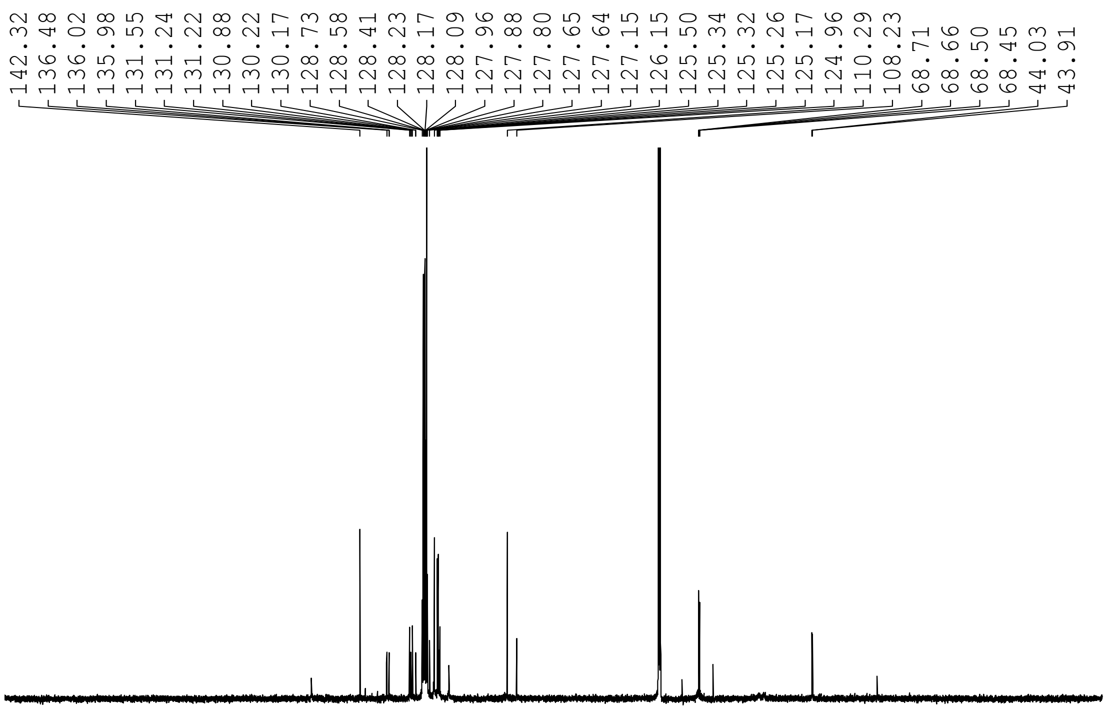

N-(p-methylphenyl)amino(pyren-1-yl)methylphosphonic Acid (**4c**)

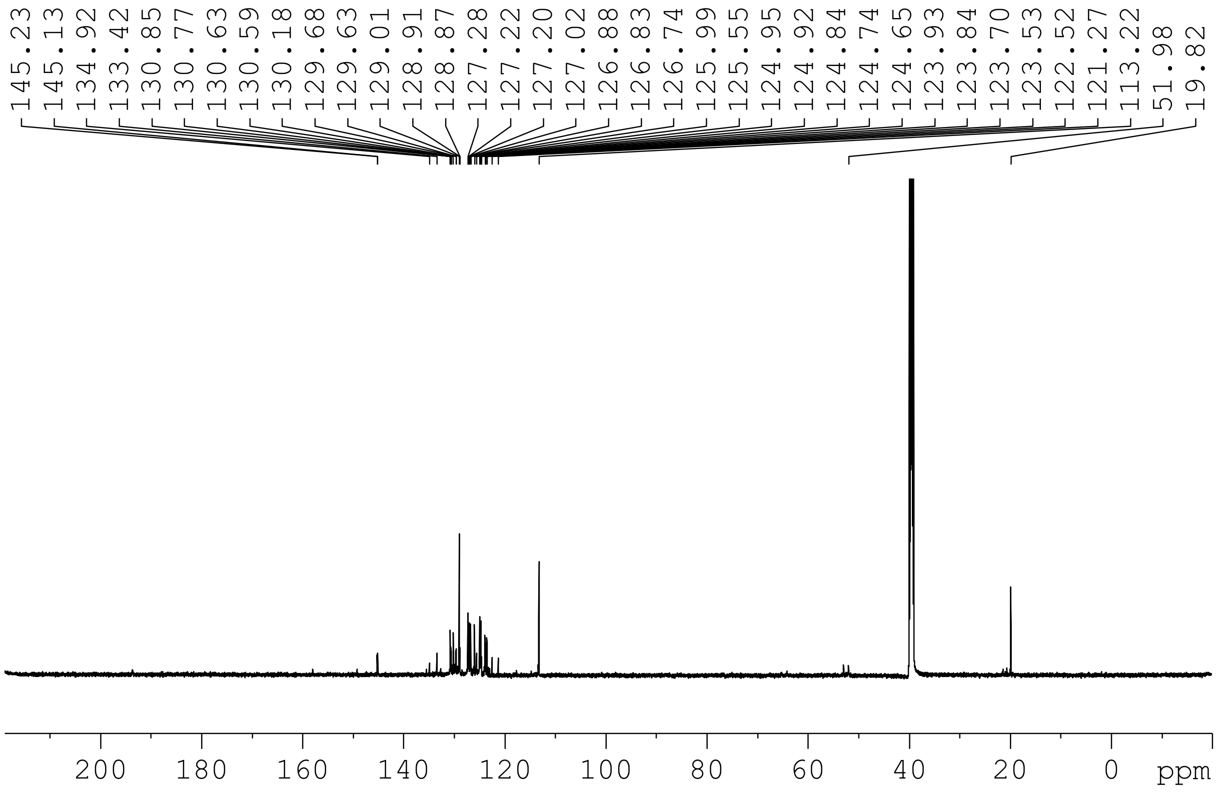

N-benzylamino(pyren-1-yl)methylphosphonic Acid (**4a**)

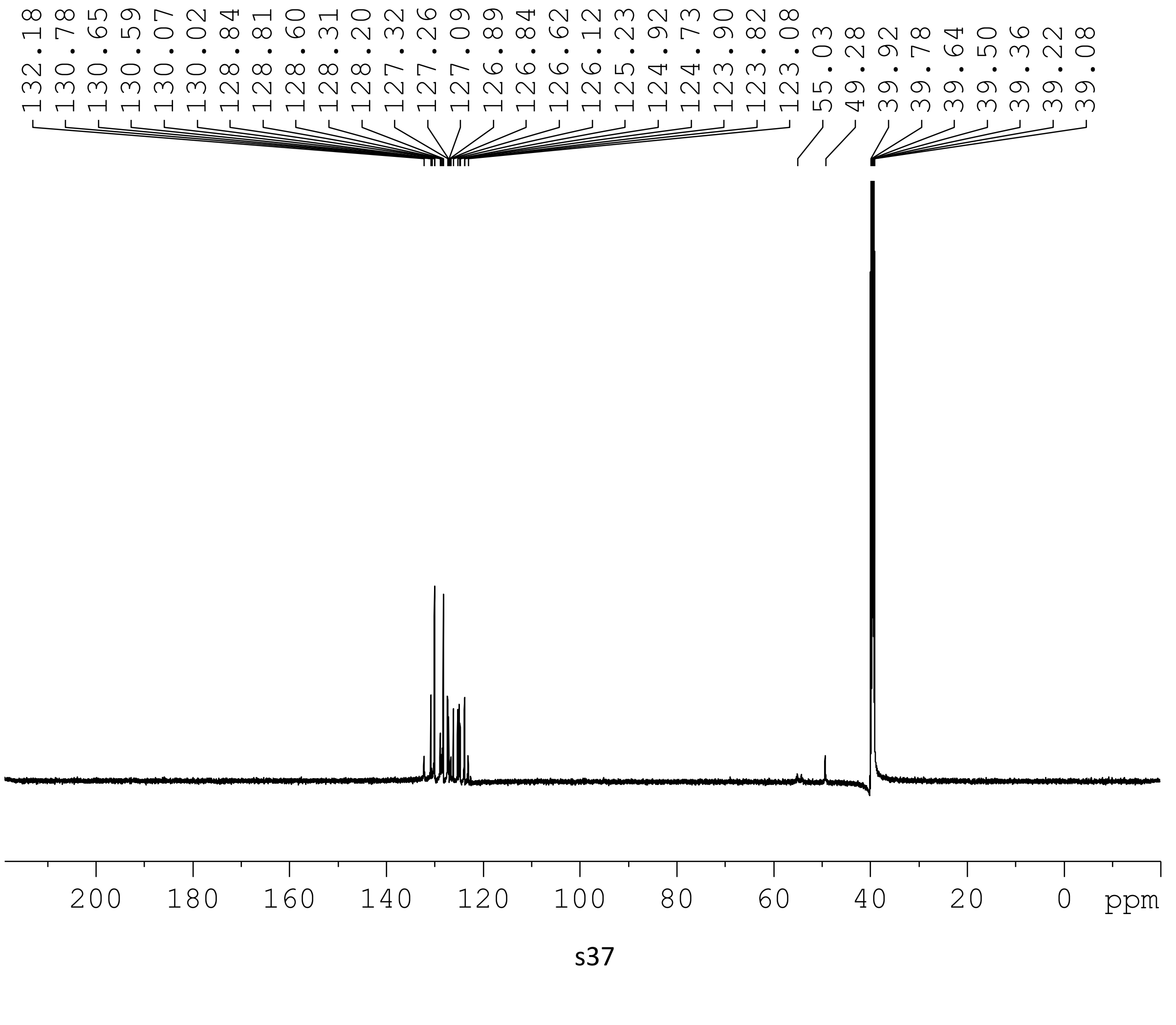

Dimethyl hydroxy(pyren-1-yl)methylphosphonate (**5A**)

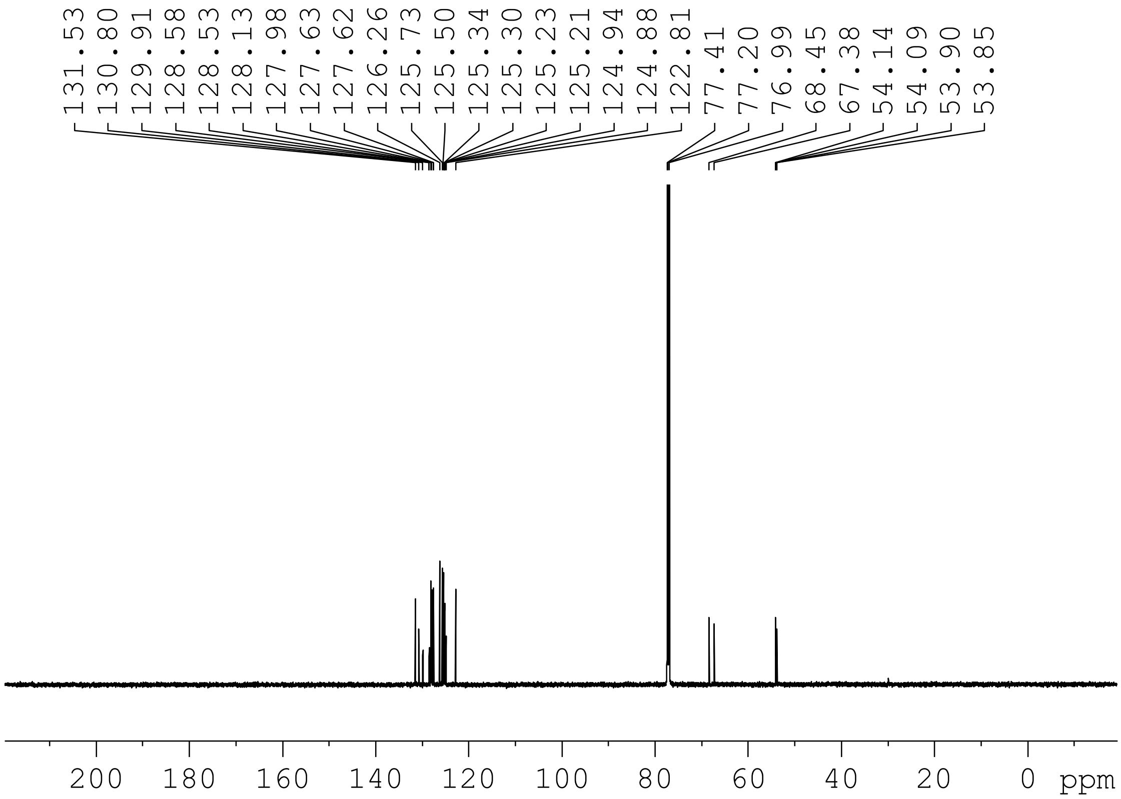

^{31}P NMR spectra of studied compounds 3, 4 and 5A

Dimethyl *N*-benzylamino(pyren-1-yl)methylphosphonate (**3Aa**)

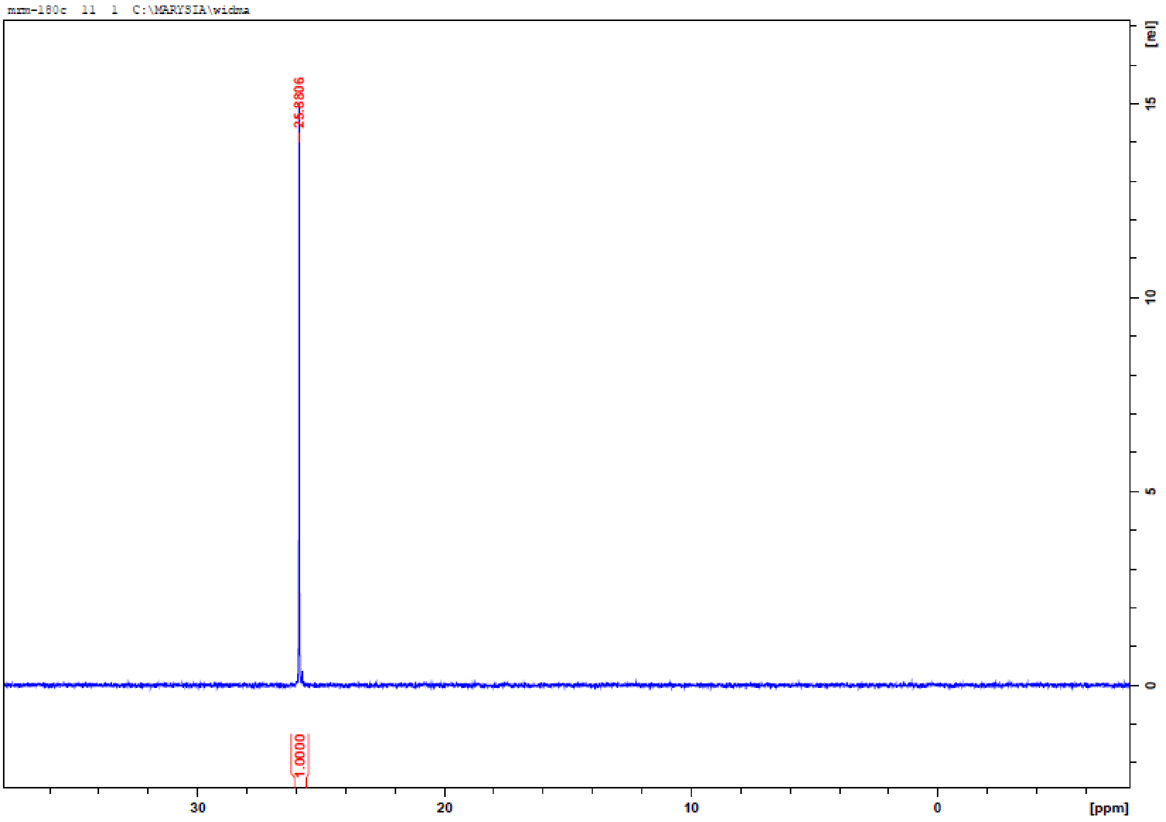

Dimethyl *N*-phenylamino(pyren-1-yl)methylphosphonate (**3Ab**)

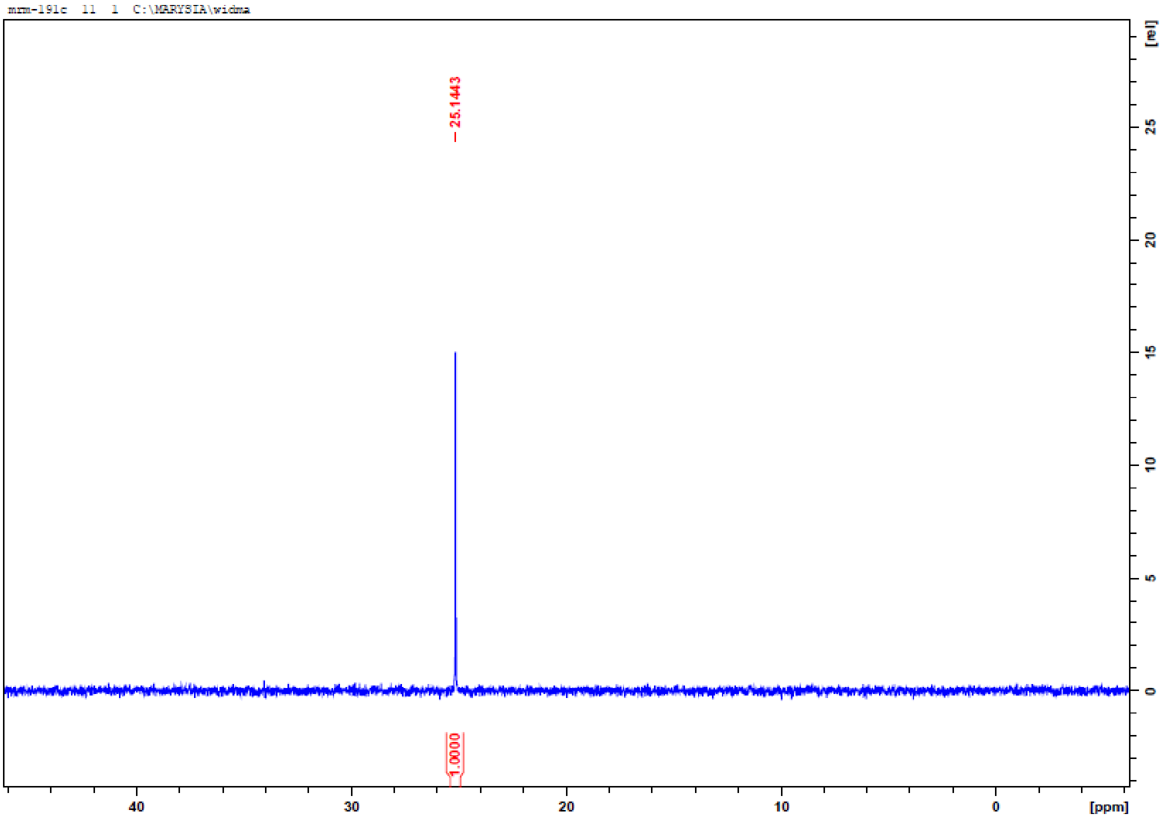

Dimethyl *N*-(4-methylphenyl)amino(pyren-1-yl)methylphosphonate (**3Ac**)

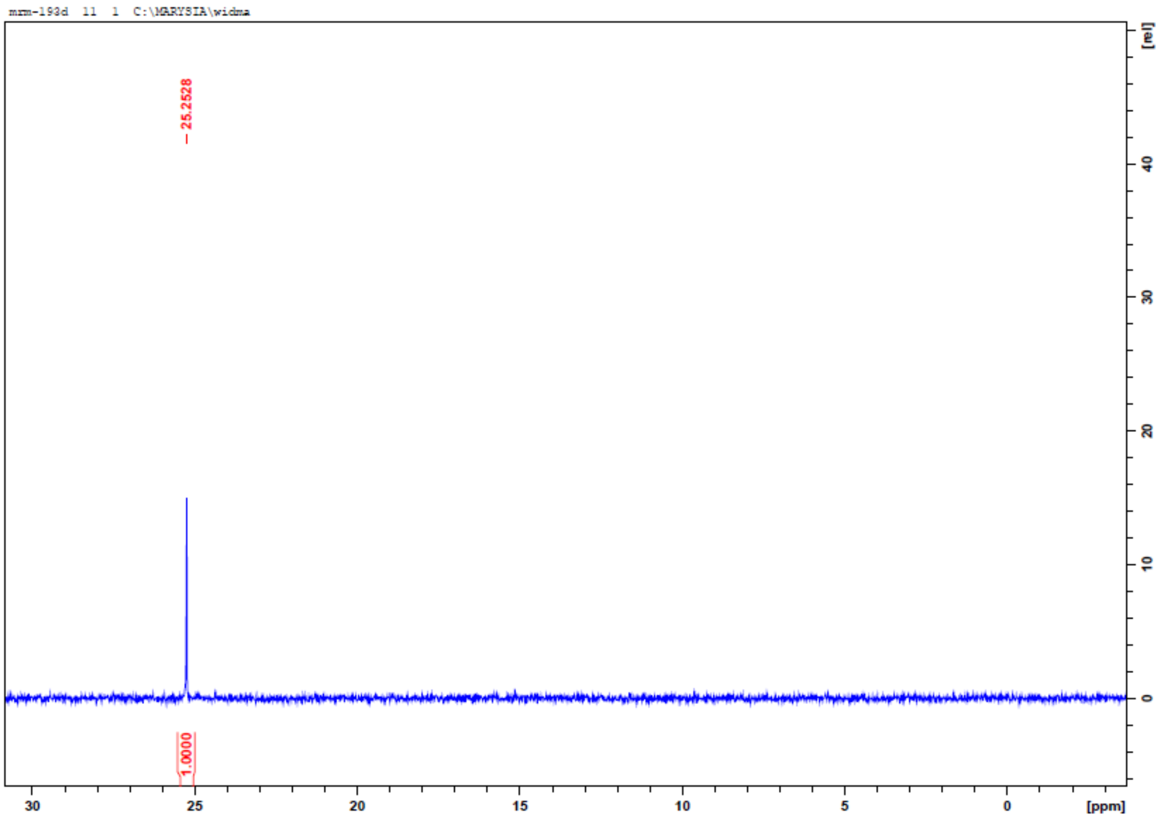

Dimethyl *N*-(4-methoxyphenyl)amino(pyren-1-yl)methylphosphonate (**3Ad**)

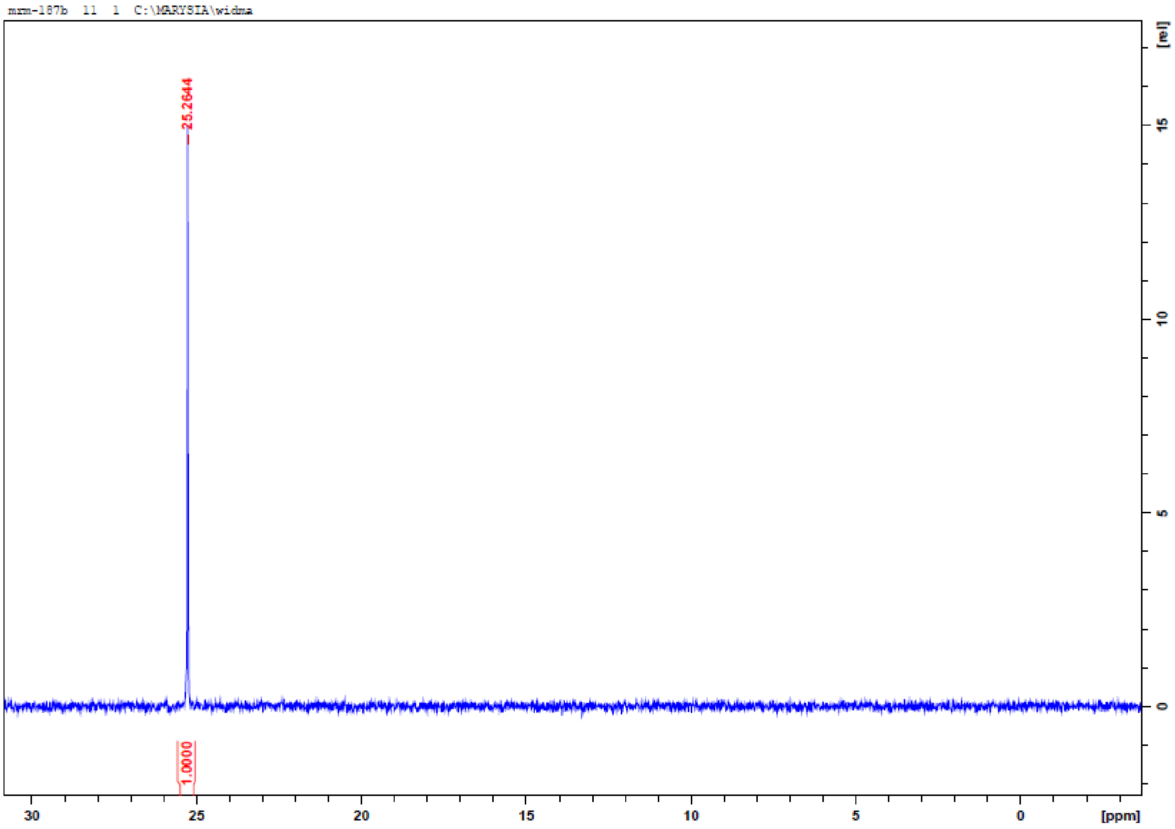

Dimethyl *N*-(1-butyl)amino(pyren-1-yl)methylphosphonate (**3Ae**)

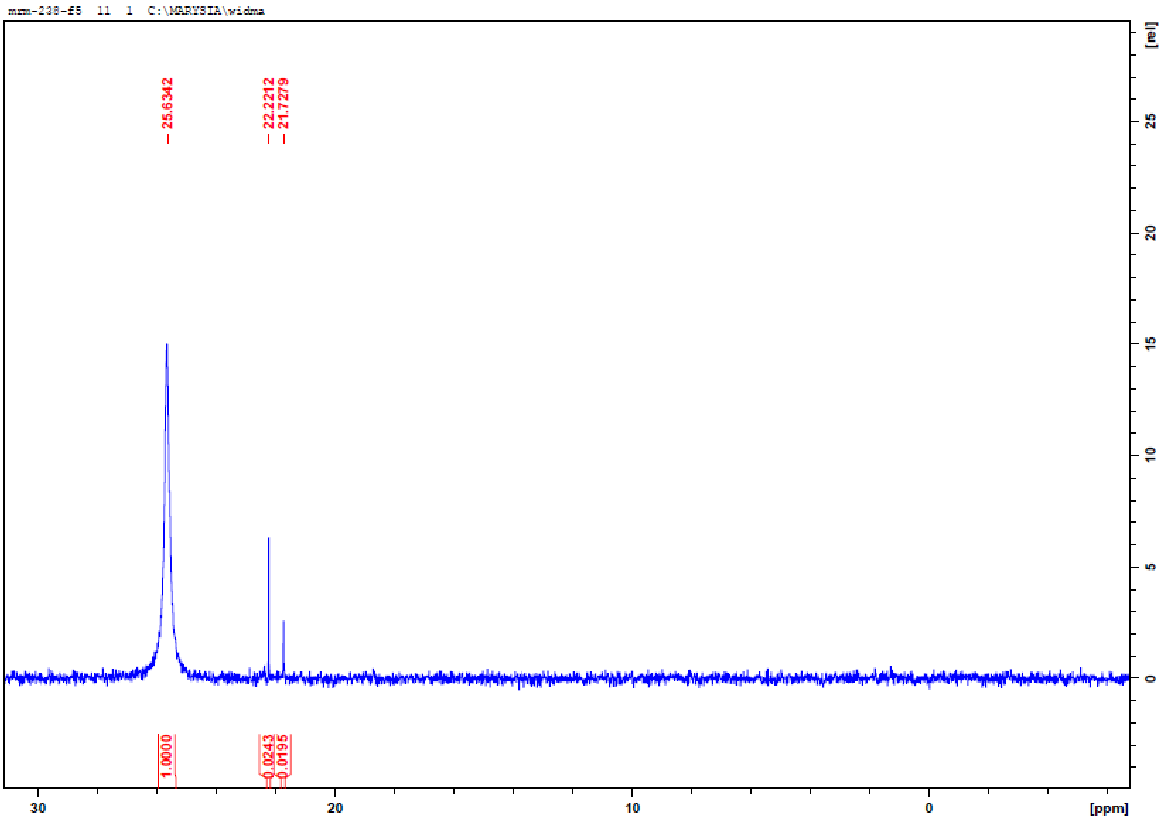

Dimethyl *N*-(1-propyl)amino(pyren-1-yl)methylphosphonate (**3Af**)

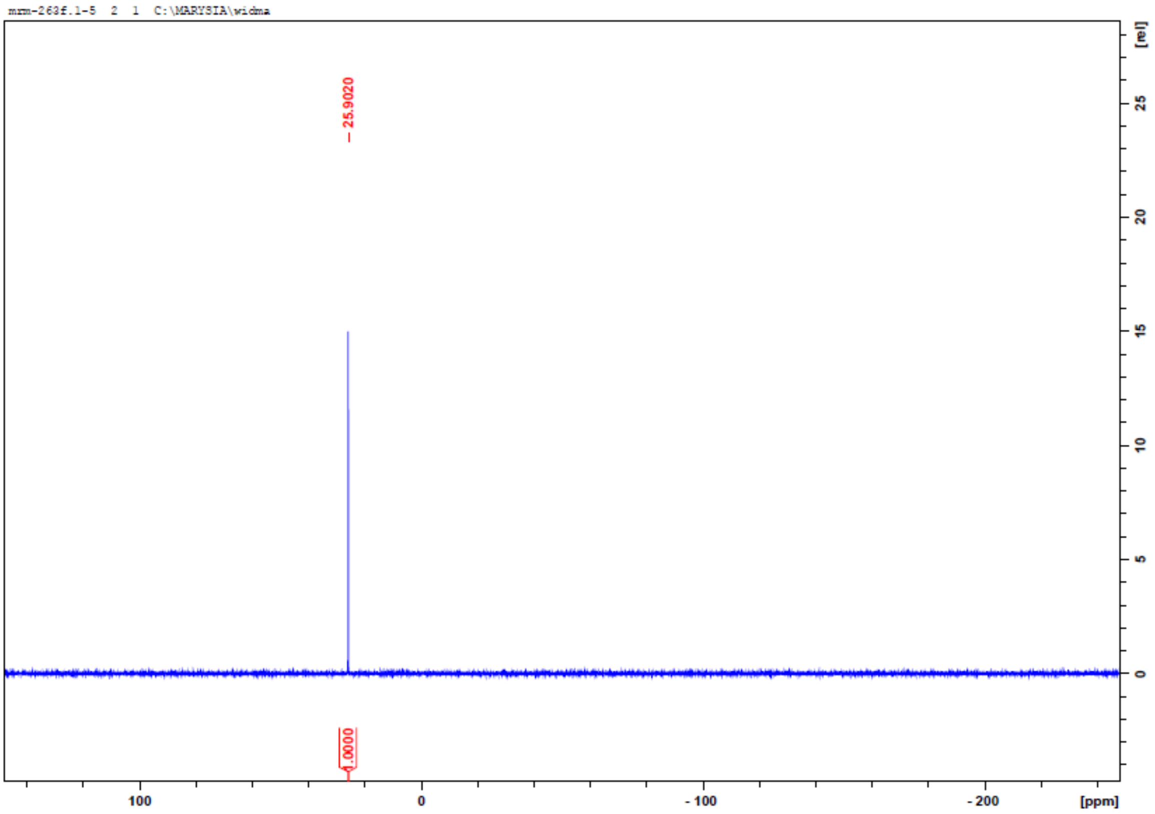

Dimethyl *N*-furfurylamino(pyren-1-yl)methylphosphonate (**3Ag**)

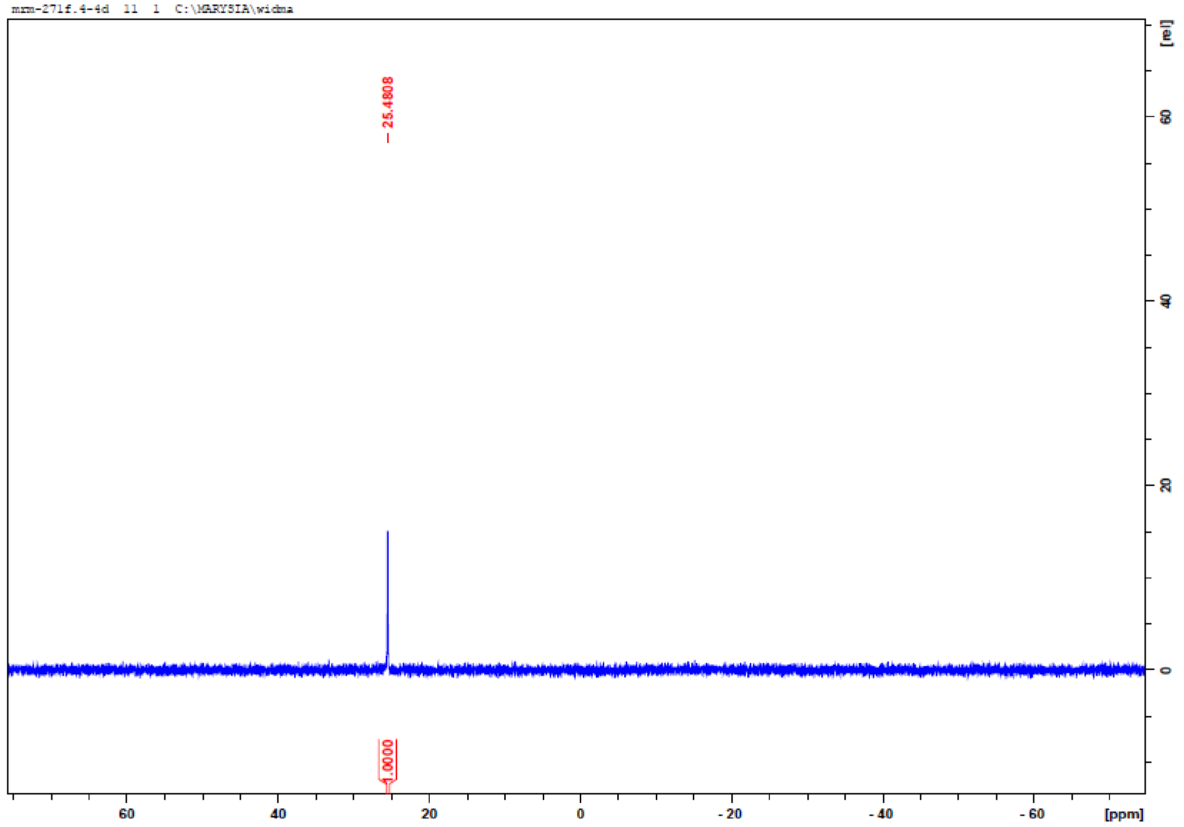

Dimethyl *N*-(3-methylphenyl)amino(pyren-1-yl)methylphosphonate (**3Ah**)

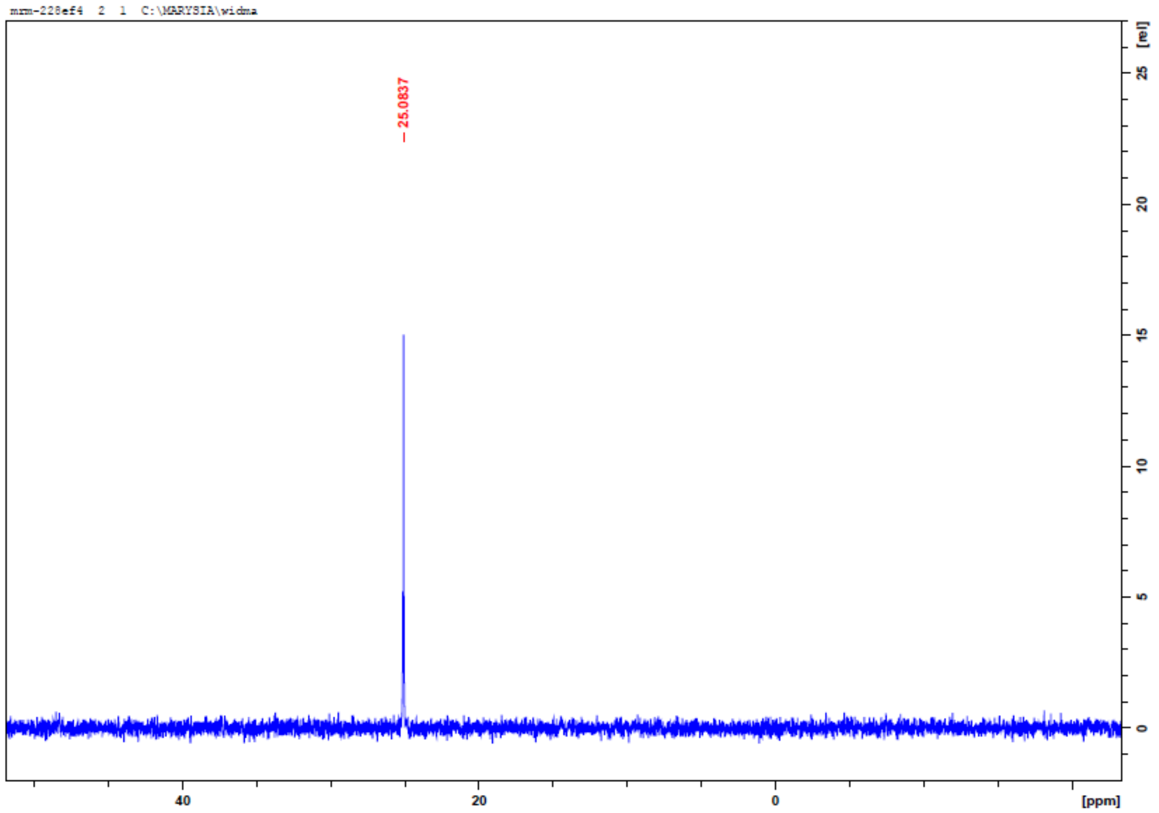

Dimethyl *N*-(*t*-butyl)amino(pyren-1-yl)methylphosphonate (**3Ai**)

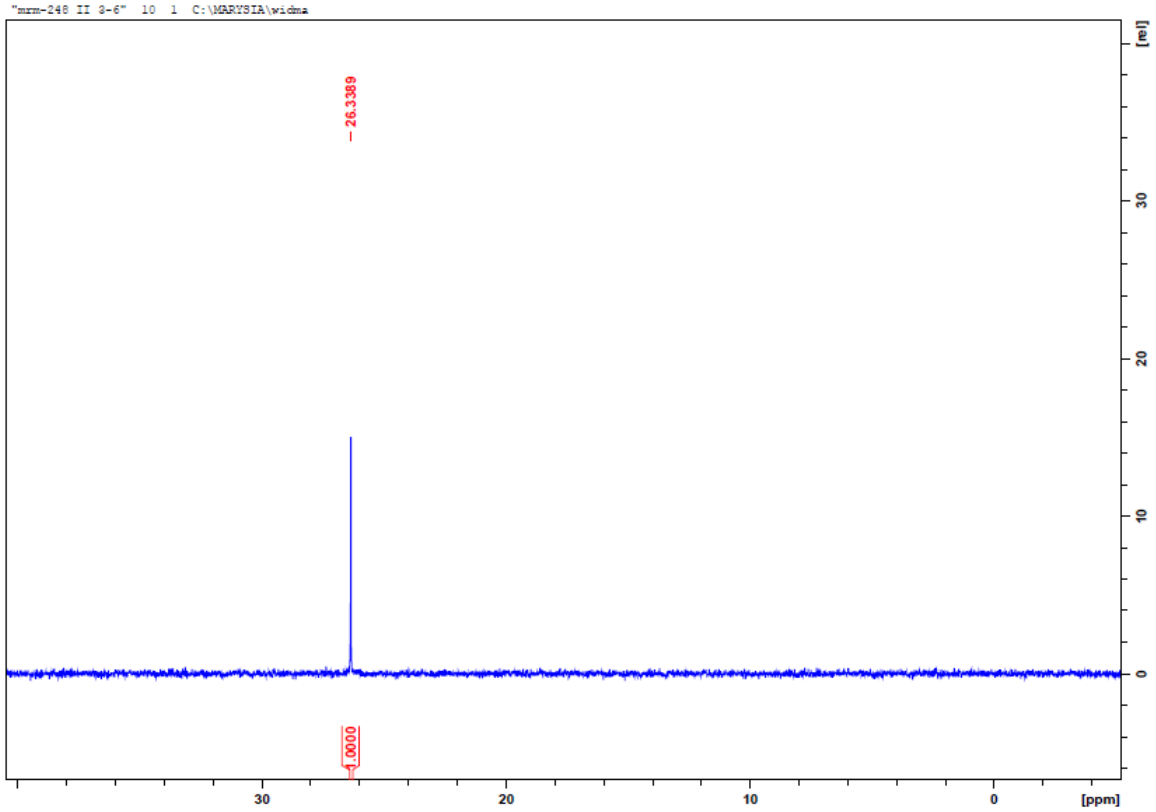

Dimethyl *N*-cyklohexylamino(pyren-1-yl)methylphosphonate (**3Aj**)

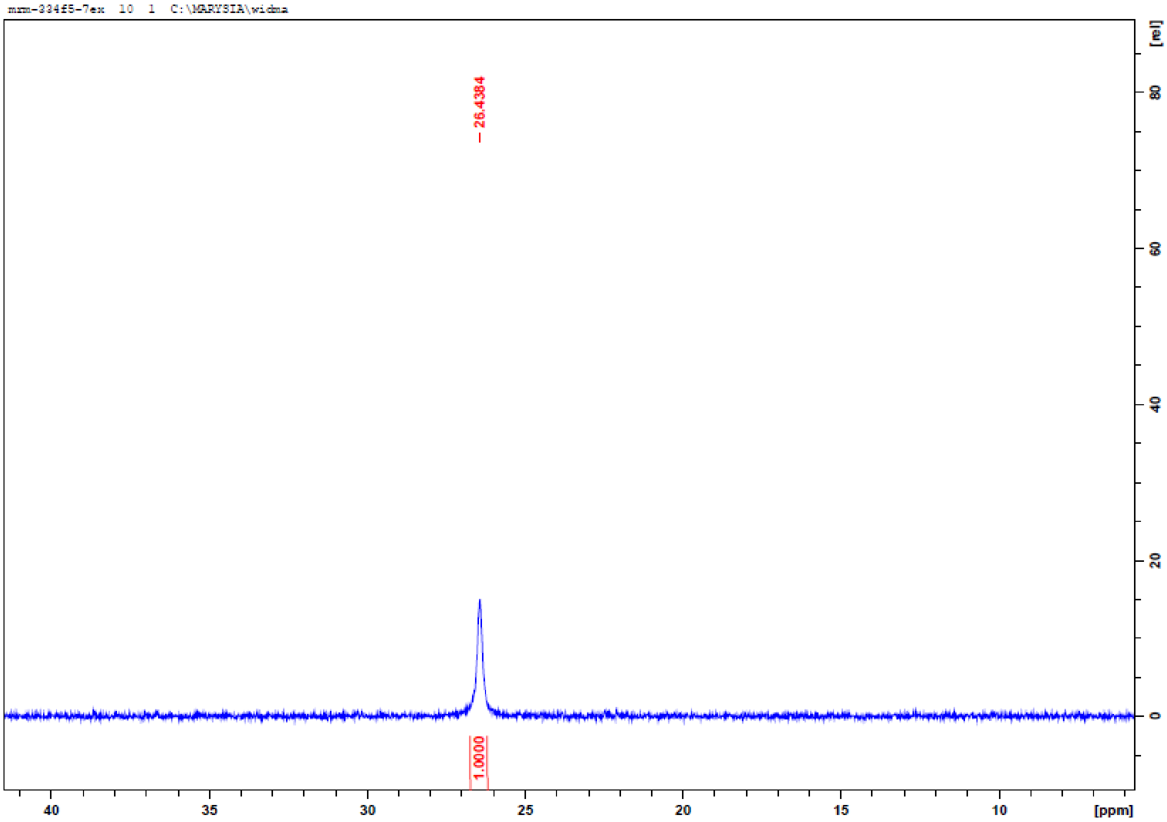

Diethyl *N*-benzylamino(pyren-1-yl)methylphosphonate (**3Ba**)

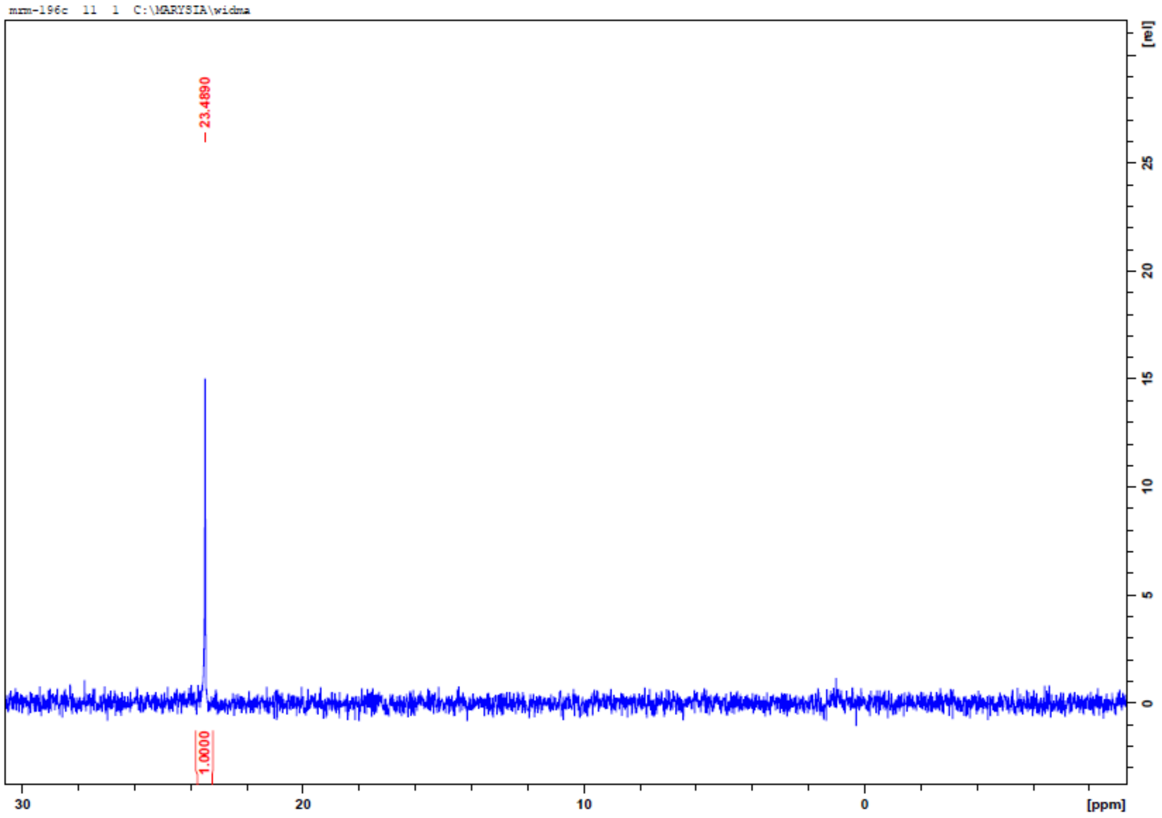

Diethyl *N*-phenylamino(pyren-1-yl)methylphosphonate (**3Bb**)

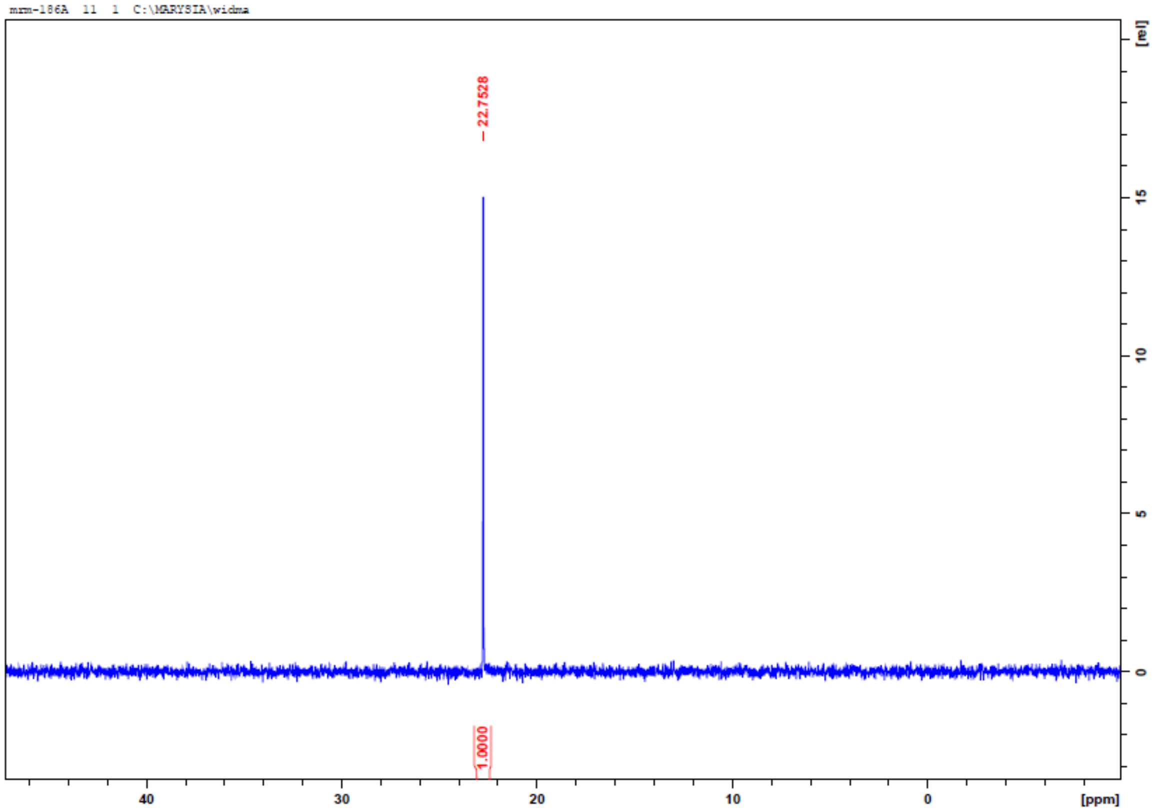

Diethyl *N*-(4-methylphenyl)amino(pyren-1-yl)methylphosphonate (**3Bc**)

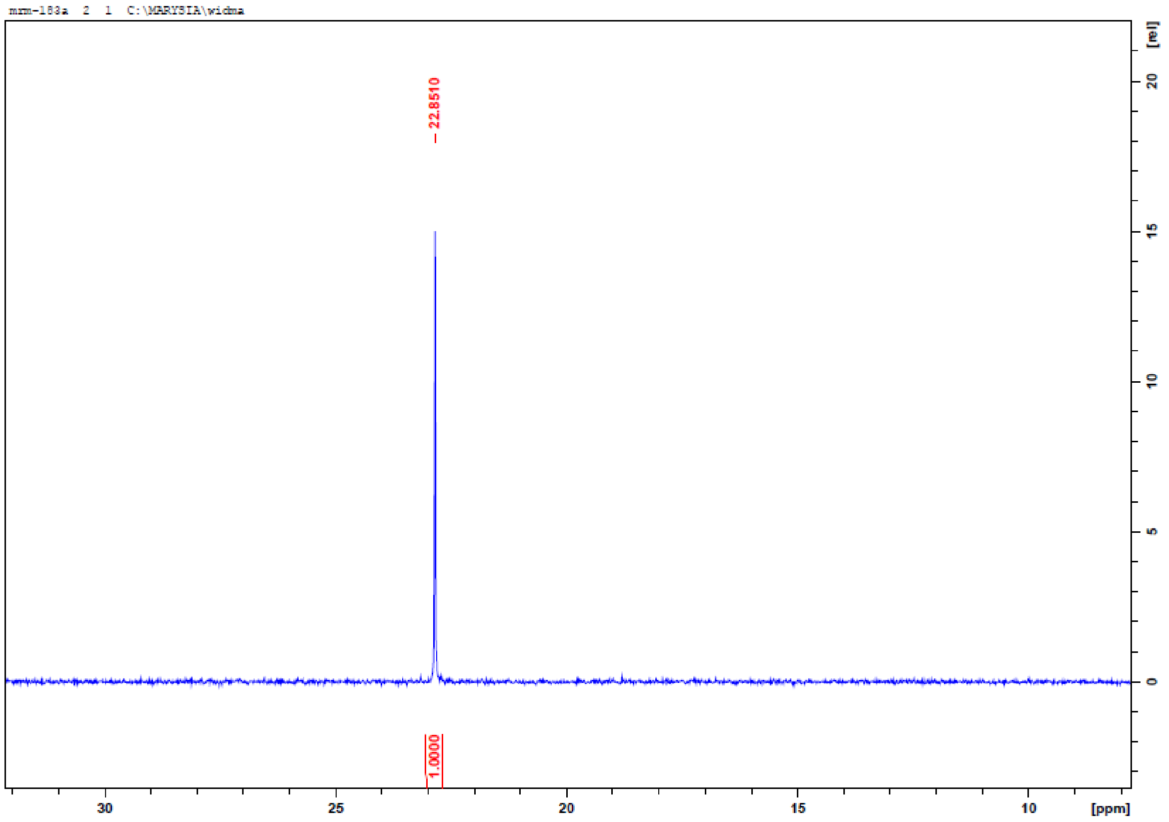

Diethyl *N*-(4-methoxyphenyl)amino(pyren-1-yl)methylphosphonate (**3Bd**)

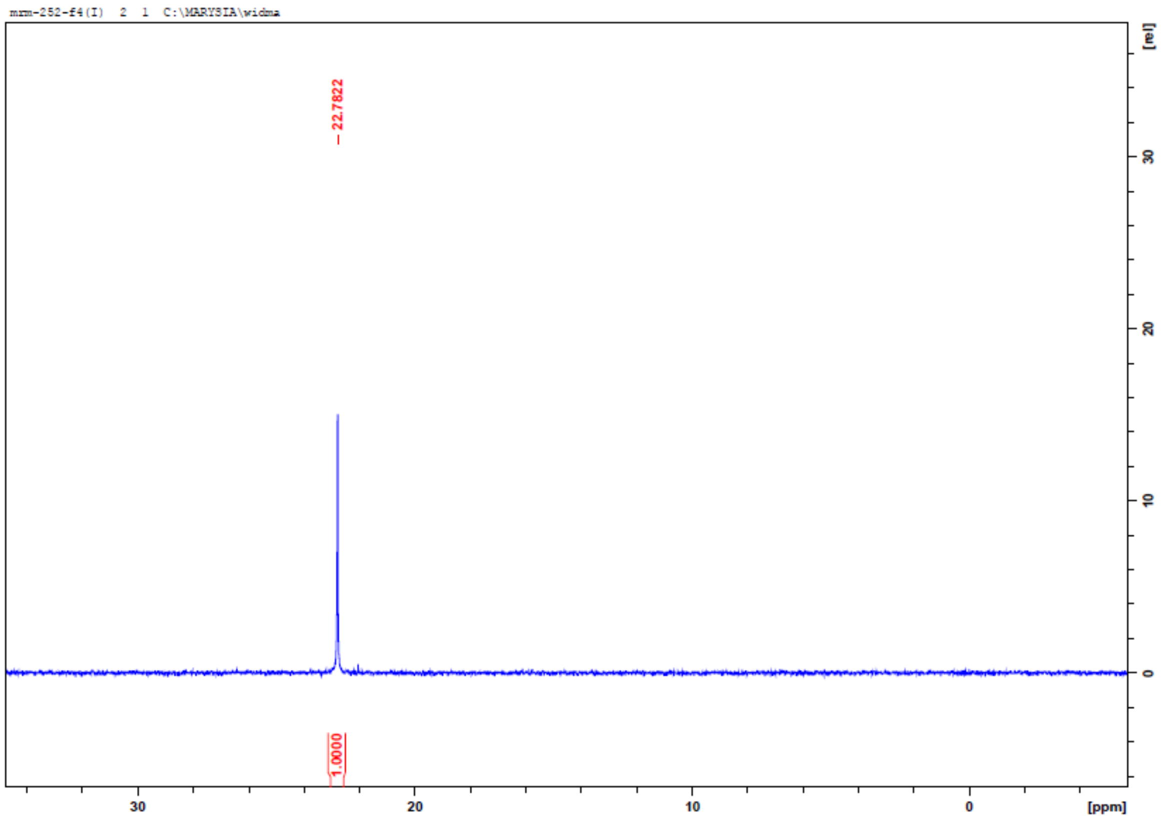

Diethyl *N*-(1-butyl)amino(pyren-1-yl)methylphosphonate (**3Be**)

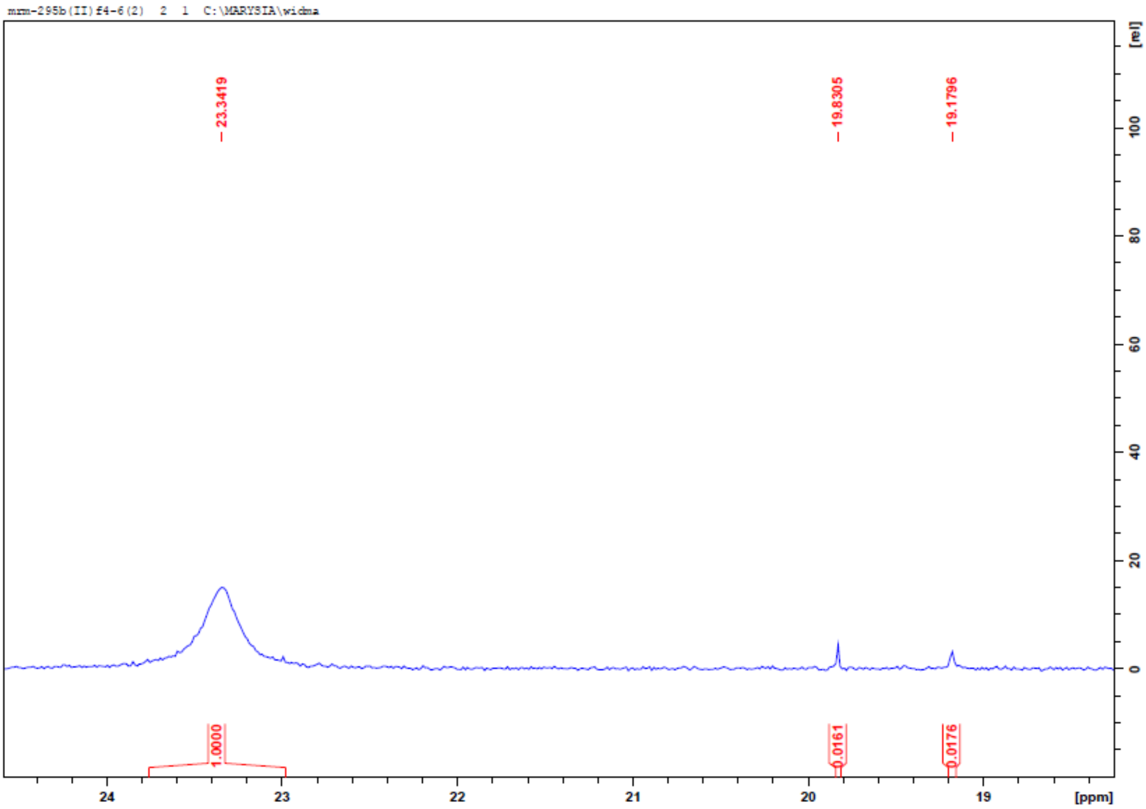

Dibenzyl *N*-benzylamino(pyren-1-yl)methylphosphonate (**3Ca**)

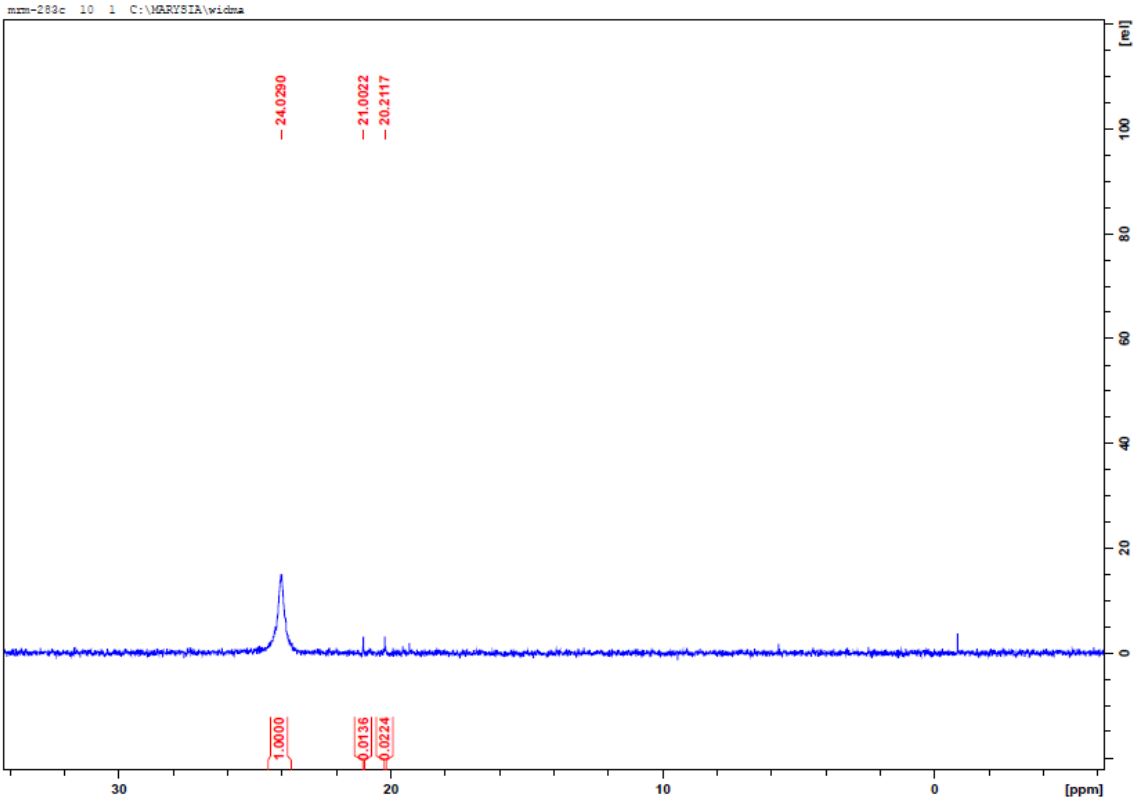

Dibenzyl *N*-phenylamino(pyren-1-yl)methylphosphonate (**3Cb**)

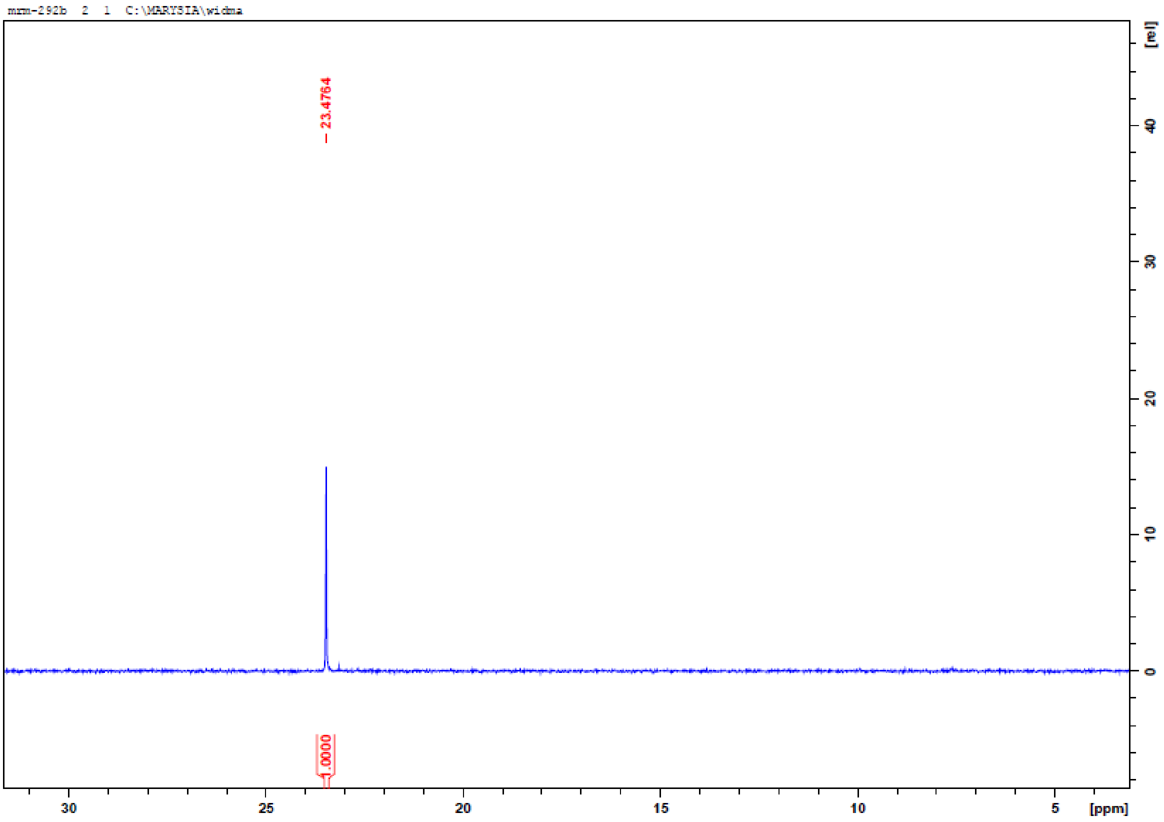

Dibenzyl *N*-(4-methylphenyl)amino(pyren-1-yl)methylphosphonate (**3Cc**)

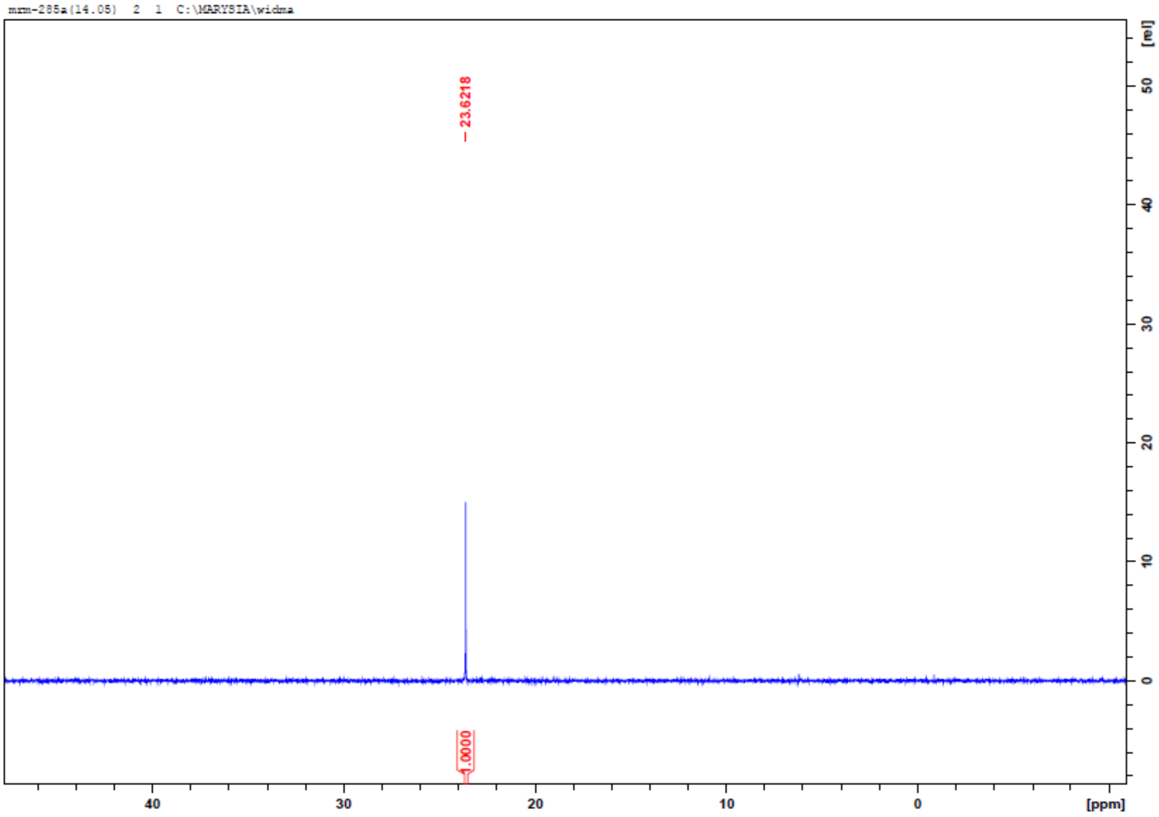

Dibenzyl *N*-(4-methoxyphenyl)amino(pyren-1-yl)methylphosphonate (**3Cd**)

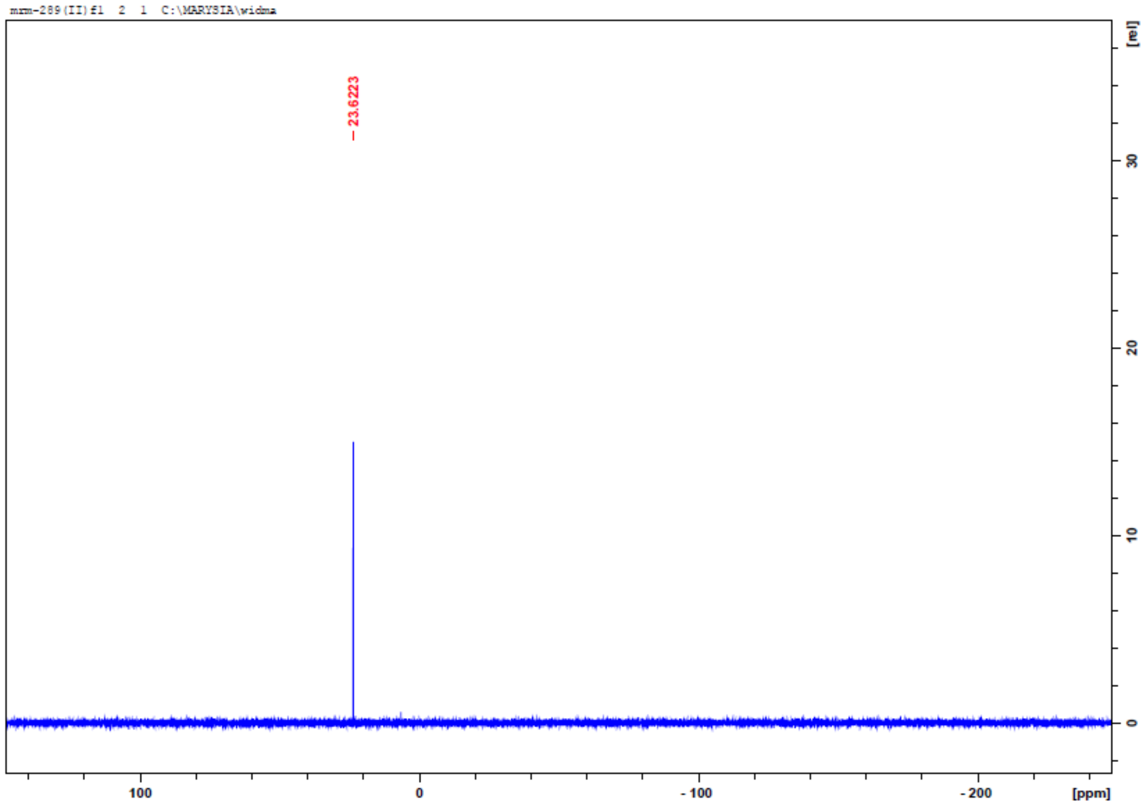

Dibenzyl *N*-furfurylamino(pyren-1-yl)methylphosphonate (**3Cg**)

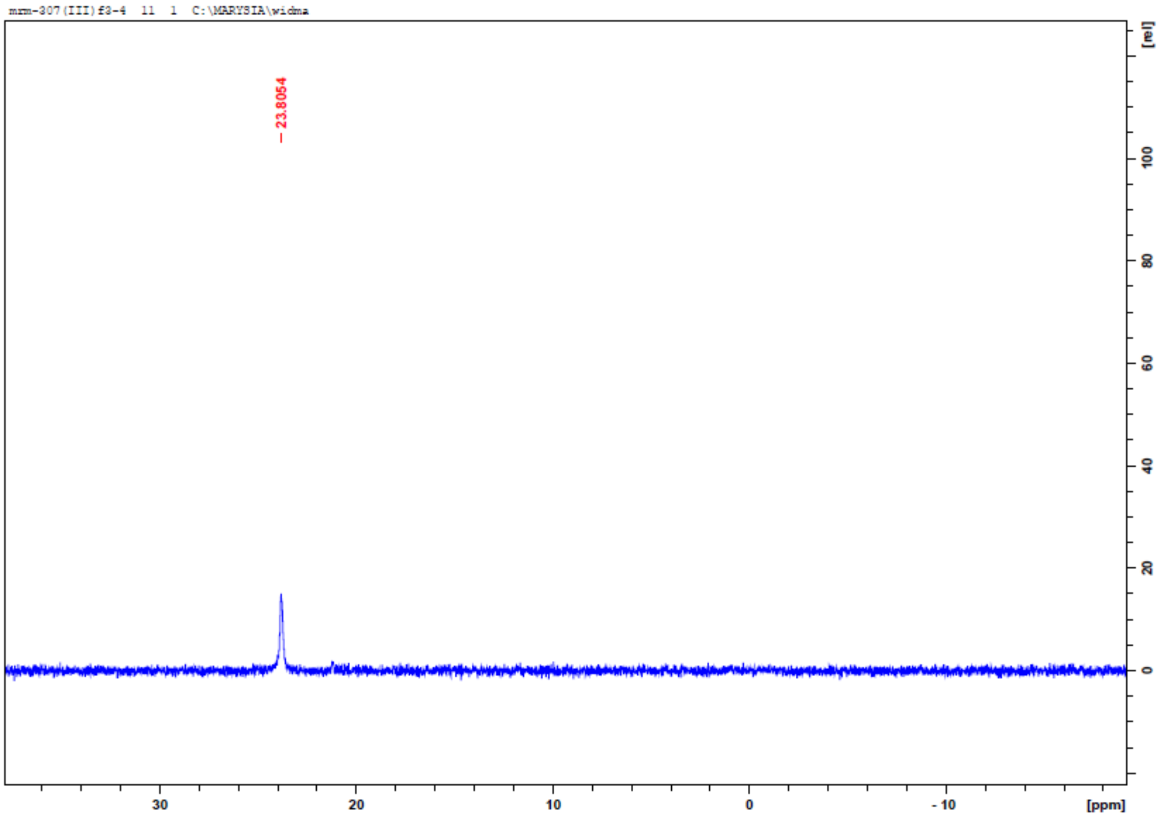

N-benzylamino(pyren-1-yl)methylphosphonic Acid (**4a**)

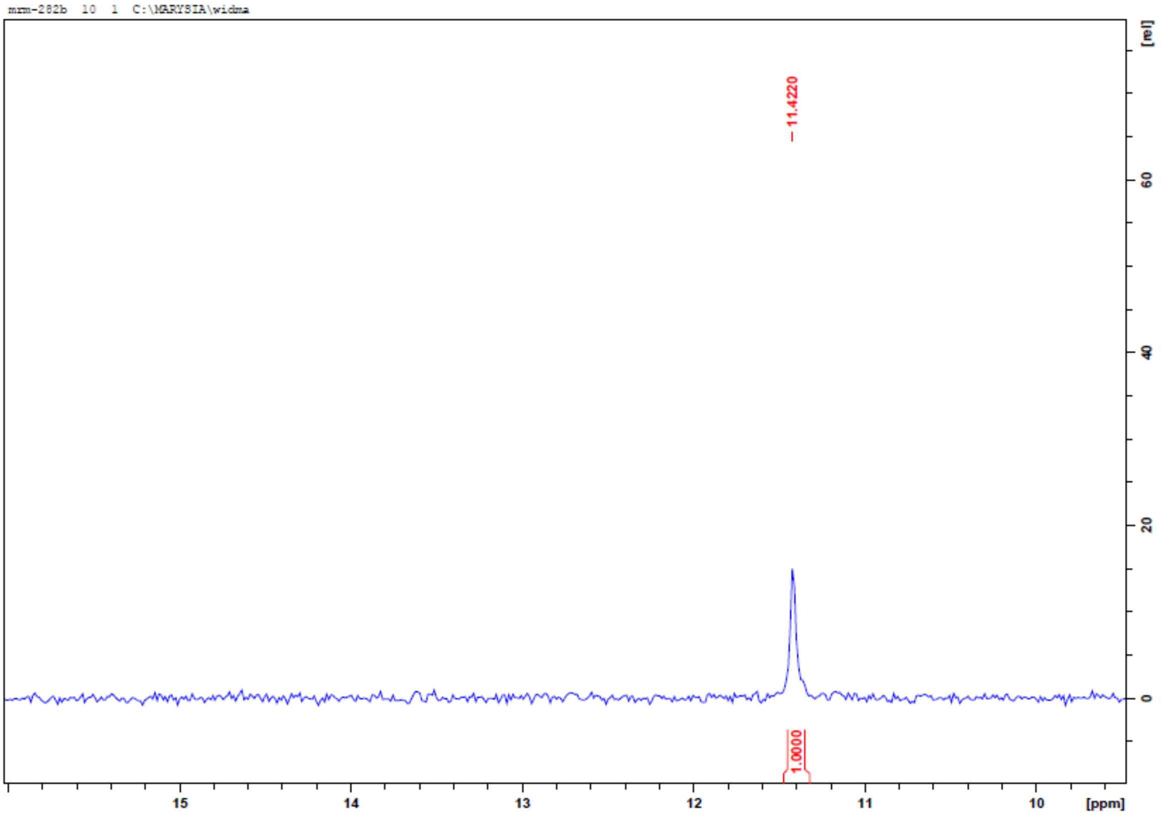

N-(p-methylphenyl)amino(pyren-1-yl)methylphosphonic Acid (**4c**)

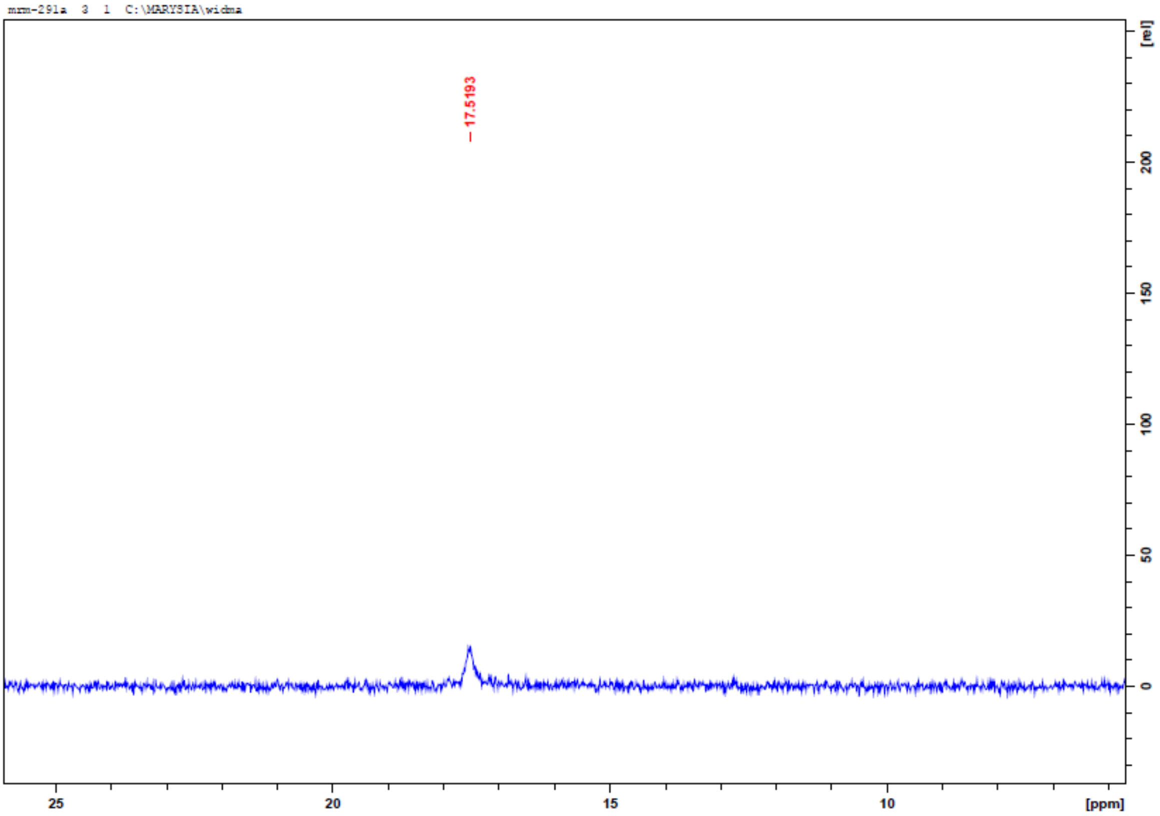

Dimethyl hydroxy(pyren-1-yl)methylphosphonate (**5A**)

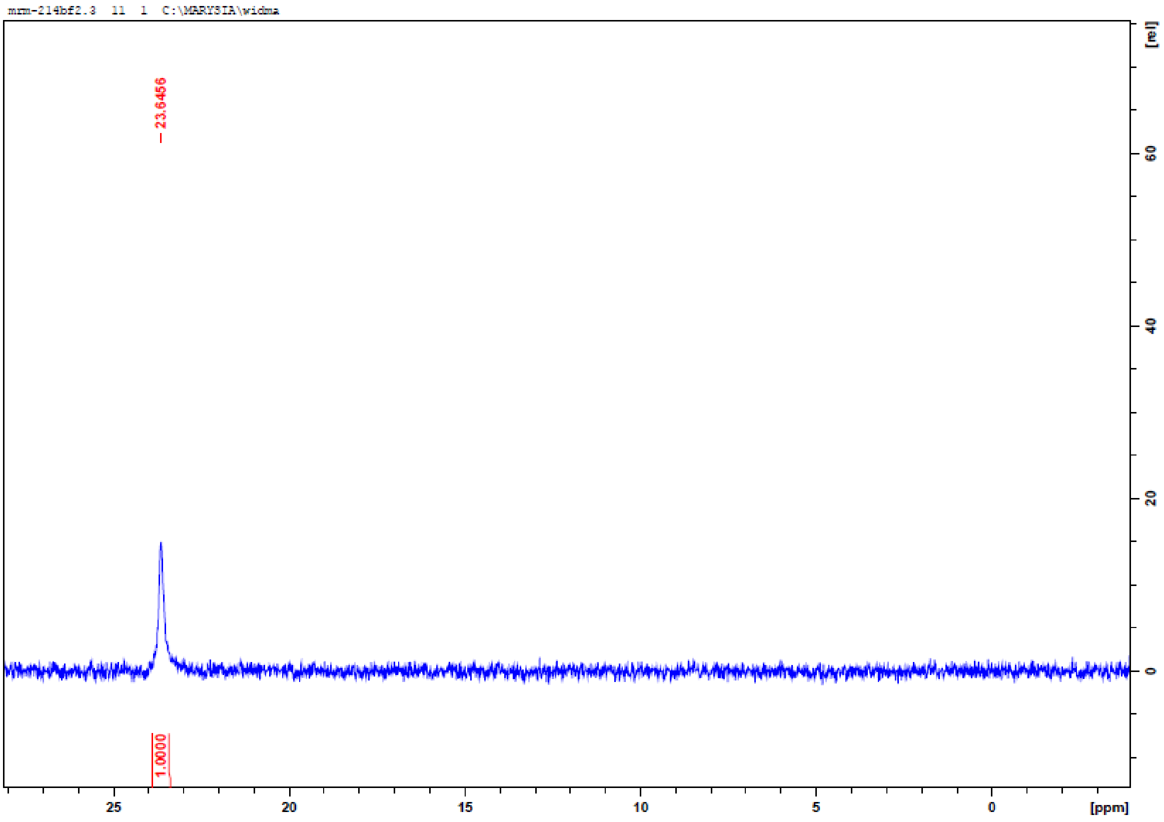

Supplement: File 1 — Experimental procedures, characterization of novel compounds, and details of the biological and photophysical study. Scans of 1H, 13C and 31P NMR spectra of all new synthesized compounds. [file Beilstein_J_Org_Chem-12-1229-s001.pdf]
